# Conformation- and Compound-Dependence of Ribose/2′-Deoxyribose Retention Patterns in Pyrimidine-Nucleoside Phosphorylase from *Bacillus subtilis*: A Four-Structure Molecular Dynamics Study

**DOI:** 10.1021/acsomega.6c08499

**Published:** 2026-09-12

**Authors:** Akihiko Hatano, Kousei Takeshima

**Affiliations:** Course of Chemistry and Biotechnology, Program of Chemistry and Materials, College of Engineering, Shibaura Institute of Technology, 307 Fukasaku, Minuma-ku, Saitama-shi, Saitama 337-8570, Japan

## Abstract

In pyrimidine-nucleoside phosphorylase (PyNP), how the conformational state shapes substrate recognition remains poorly understood. In this study, all-atom molecular dynamics (MD) with MM-PB­(GB)­SA decomposition was performed on four structures of PyNP from *Bacillus subtilis* spanning the conformational range: the open 5OLN, the semi-open 5EP8orig, an energy-minimized contracted (closed-like) model 5EP8 min, and the cross-species closed-state crystal 1BRW, the last added to define the closed-state end. Thirteen structural probes were used (ten purine-like ribose/2′-deoxyribose analogues and three inhibitors), sampling 156 trajectories and 15.6 μs in aggregate. All raw trajectories were deposited in a public repository (Zenodo, 10.5281/zenodo.21448920). The main results were threefold. (1) Sugar-ring puckering analysis showed that, overall, ribose preferred C3′-endo (north, 64%) and 2′-deoxyribose preferred C2′-endo (south, 58%) (*p* = 0.021). However, this per-sugar difference was largest for the unsubstituted sugar and shrank as the 6-position substituent became larger; sugar-ring puckering therefore provides a molecular correlate of sugar discrimination in this probe set, but its extent depends on the 6-position substituent. (2) Ligand retention was high at the closed-state end. However, the ribose-versus-2′-deoxyribose preference was not a property of the whole active site but a compound-by-compound and conformation-by-conformation property: averaged across compounds, and for many between-state differences, it was not statistically significant at the compound level. (3) Tyr165 behaved as a lid at the closed-state end; however, this signal arose mainly from the cross-species 1BRW structure and lost significance when 1BRW was excluded, so it requires confirmation with a closed-state structure of the same species. These retention metrics measure not equilibrium affinity but dynamic retention over 100 ns (the fraction of frames in which the ligand remained in the active site). This study clarifies how the retention, contact, and sugar preference of these structural probes depend on the conformational state and does not directly reproduce the experimentally reported ribose-donor selectivity. To test these conclusions, three sets of additional all-atom MD (each in three replicas) were performed. (i) The falsifiable mutant prediction was tested by Ala-mutant MD: Y165A, with the lid residue removed, showed significantly reduced ligand retention (BF_late median 0.02; one-sided *p* = 0.049 versus the control mutants retaining Tyr165). (ii) In a control MD explicitly including the co-substrate phosphate (HPO4^2–^), the phosphate bound to the Lys cluster and could approach the anomeric carbon C1′ closely (contact fraction up to 96%; closest approach 2.81 Å), but the reactive in-line attack geometry required for substitution was essentially never adopted (below 0.5% of contact frames; 0% in 30 of the 33 systems), consistent with the interpretation that ribose-donor selectivity is decided at the transition state and is not accessible to equilibrium MD, pointing to the need for QM/MM or transition-state-level analysis. (iii) Extending the central systems to 300 ns gave retention and sugar-preference conclusions identical to the 100 ns analysis, supporting (rather than confirming) the conclusions obtained at the 100 ns scale; only a subset of central systems was extended, and only 46% of the original systems reached a plateau, so these extensions are reported as consistent with the 100 ns results. On this basis, three falsifiable mutagenesis predictions (Y165A, Q153A, K81A/K108A/K188A) are proposed.

## Graphical abstract

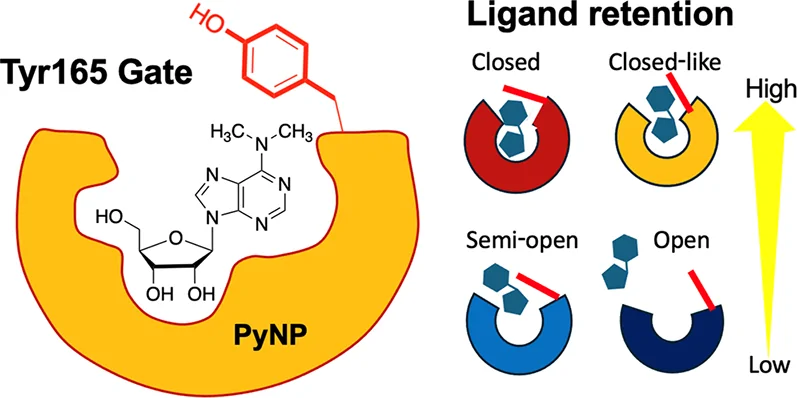

## 1 Introduction

Nucleoside phosphorylases catalyze the reversible phosphorolysis of a nucleoside into a free base and ribose-1-phosphate. Pyrimidine-nucleoside phosphorylase (PyNP, EC 2.4.2.2) is a central enzyme in pyrimidine salvage and has a proven record as a target for chemotherapy and antibacterial design. PyNP from *Bacillus subtilis* has been crystallized by X-ray in several conformations spanning the catalytic cycle (the closed state is represented by the structure of a closely related species; Section 2.1), making it particularly attractive as one of the rare opportunities to study induced-fit dynamics at residue resolution.

Three crystal structures of PyNP have been deposited to date, from which we construct four structures spanning the conformational range (three experimental crystal structures and one energy-minimized closed-like model). 5EP8 (PDB ID 5EP8)^2^ was determined with a sulfate ion in the phosphate subsite and provides a semi-open crystal state (used directly as 5EP8orig). From it, and prior to any simulation, we derived an energy-minimized closed-like model (5EP8min; Methods Section 2.1). 5OLN^3^ represents the open state; the deposited structure contains imidazole, 1,2-ethanediol, and a sulfate ion, and the chain used here (chain A) contains imidazole and 1,2-ethanediol (all crystallization additives were removed before simulation; Methods Section 2.1). In addition, because a closed-state crystal of the *B. subtilis* enzyme is not available, 1BRW was included solely to define the closed-state end of the conformational range. 1BRW is the closed-state crystal of the closely related *Geobacillus stearothermophilus* enzyme^4^ (about 69% sequence identity with *B. subtilis* PyNP; 68.7% in the global alignment of the full-length monomer used here), bound to uracil and phosphate (the products of phosphorolysis). Although the full-length identity is only about 69%, the seven residues that constitute the active site (Asp80, Lys81, His82, Lys108, Gln153, Tyr165, Lys188) are all strictly conserved with the same residue numbering (Table S15), so 1BRW provides a reasonable closed-state reference for the conserved active-site residues; it does not, however, fully remove species-, product-bound-state-, or conformational-context differences from the *B. subtilis* enzyme. Conformational claims concerning the experimentally studied enzyme are therefore based on the *B. subtilis* structures (5EP8, 5OLN), and 1BRW is used as a cross-species closed-state reference. These four conformations are connected by a hinge-like motion of the α7−α8 loop that moves Tyr165 either over the substrate (closed state) or into the solvent (open state).

Our group has previously shown that PyNPs and thymidine phosphorylase (TP) can synthesize a wide range of non-natural nucleosides, using thymidine or uridine as the ribose donor and non-natural uracil or purine bases as substrates. In particular, we reported that modified bases in which a fluorescent dye is tethered to uracil through an alkyl linker at the 5-position undergo the base-exchange reaction catalyzed by PyNP and TP.^5^ We similarly showed that pseudobases such as stable-isotope-labeled uracil bases^6^ and benzimidazole^7^ are accepted as substrates. More recently, we found that 6-alkylamino modification of the purine base generates a selectivity (Thd/Urd value) between thymidine (the deoxy form) and uridine (the ribo form) as the ribose donor.^1^ That is, in this two-substrate reaction, the structure of one substrate governs the reactivity of the other, but the molecular basis of this coupling remains unknown.

We reasoned that analyzing how substrate analogues behave within the active-site pocket by molecular simulation would provide insight into the structural factors that determine substrate engagement. The crystal structures simulated as experimentally relevant states (5EP8, 5OLN) are of the same *B. subtilis* enzyme, so the structural context is homologous even though the bound probes are not cognate substrates. However, the simulations in this study used purine-base rR/dR analogues as structural probes and did not include the cognate pyrimidine substrates (thymidine, uridine). The co-substrate phosphate was not included in the original simulations, but the behavior in the presence of phosphate was examined by the HPO_4_^2–^ control MD added in this study (Section 3.5). This study therefore does not directly reproduce the experimentally reported ribose-donor selectivity^1^ but rather investigates its relationship to the conformational dependence of these probes.

Previous computational studies of nucleoside phosphorylases have focused either on single-structure docking screens or on isolated trajectories of only one state at a time.^8^ Two limitations arise from this: First, the contribution of “ribose puckering (conformation)” and the contribution of “specific residue interactions” to substrate preference have not been separated. Second, the uridine (ribo)- versus thymidine (deoxy) selectivity of PyNP has a small preference magnitude (Thd/Urd ratio) as observed experimentally and has not been mechanistically analyzed at the per-residue level.

In this study, an integrated MD campaign of 4 structures × 13 ligands fills these two gaps: (i) the relative contributions of the conformational state and of specific residues and (ii) the per-residue mechanism of rR/dR selectivity. We analyzed a total of 156 independent 100 ns trajectories (*n* = 3 replicas per system, 15.6 μs of sampling in aggregate; Figure 1). In addition, to test the robustness of these conclusions, three sets of additional MD were performed: Ala mutants, a co-substrate phosphate (HPO_4_^2–^) control, and a 300 ns extension of the central systems (Section 3.5). The overall conformational picture was quantified with a sphere-packing pocket-volume metric, ligand retention was characterized with a contact-based binding fraction, and the per-residue contributions were decomposed. The main findings are as follows. (i) The active-site pocket expanded monotonically from the closed to the open state (approximately 1.4-fold by the sphere-packing metric; the robust finding is the ranking rather than the absolute fold factor; see Section 3.1). (ii) Ligand retention was significantly higher in the closed state. (iii) Tyr165 is the principal lid residue, and its closed-state-specific contact signal arose mainly from 1BRW (functionally closed). (iv) Ribose-versus-2′-deoxyribose preference is a compound-by-compound and conformation-by-conformation property, appears most sharply in the semi-open *B. subtilis* state (5EP8orig), and is organized by a four-pattern classification. Finally, we close with three falsifiable mutational predictions.

**Figure 1:**
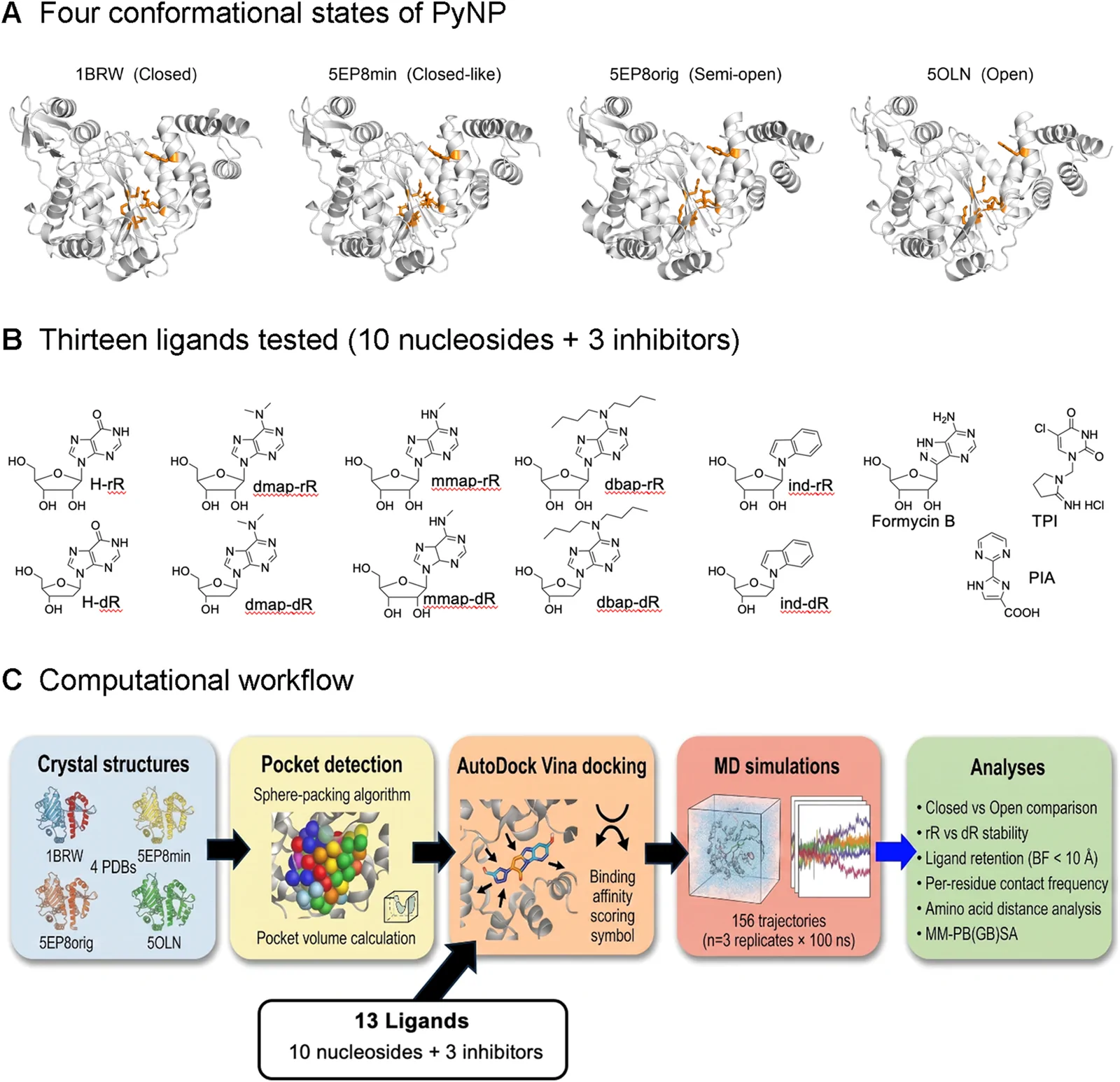
Overview of the system and computational workflow. (A) Four structures of PyNP used in this study (three crystal structures and one energy-minimized model), shown as cartoon ribbons with the gate loop (residues 153–170) colored per state and the seven active-site residues highlighted as orange Cα spheres. (B) The 13 ligands: ten substrate analogues (top, ribose [rR]; middle, 2′-deoxyribose [dR]) and three inhibitors (bottom: formycin B, TPI, PIA). (C) Computational workflow. Pocket volumes were determined for each crystal structure by a sphere-packing algorithm. Each of the 13 ligands was docked into each of the 4 conformations and subjected to 100 ns molecular dynamics in triplicate, giving 156 production trajectories (15.6 μs cumulative). Trajectories were analyzed by minimum distance to the seven active-site residues, the bound fraction below 10 Å, per-residue contact frequencies, MM-PB­(GB)­SA binding energies, and a four-pattern classification (Patterns A/B/C/D) that captures the rR-vs-dR preference for each compound.

## 2 Materials and Methods

### 2.1 Protein Structures and Preparation

Three X-ray crystal structures of pyrimidine-nucleoside phosphorylase (PyNP) were retrieved from the Protein Data Bank to represent distinct conformational states. 1BRW (PyNP from *G. stearothermophilus*, in complex with uracil and phosphate, 2.13 Å; assigned here to the closed state),^4^ 5EP8 (PyNP from *Bacillus subtilis*, co-crystallized with a sulfate ion, 2.66 Å; semi-open state),^2^ and 5OLN (PyNP; the deposited structure contains imidazole, 1,2-ethanediol, and a sulfate ion, and the chain A used here contains imidazole and 1,2-ethanediol, 1.88 Å; open state)^3^ were used. Although 1BRW is derived from *G. stearothermophilus* rather than *B. subtilis*, the two enzymes are highly similar. The 1BRW sequence shares 69% identity with the *B. subtilis* enzyme (5EP8/5OLN) across the 433-residue catalytic α/β fold, and their backbones are nearly superimposable (Cα RMSD 2.1 Å between 1BRW and 5EP8, and 2.7 Å between 1BRW and 5OLN, the latter difference reflecting mainly the closed-to-open conformational change rather than species divergence). All seven active-site residues used in the subsequent analysis were strictly conserved across the three structures, as confirmed by a residue-by-residue comparison of the superimposed structures (the active-site pocket, conserved across the four conformational states, is shown in Figure S1). Small-molecule species present in the crystals (the sulfate ion in 5EP8, and the imidazole and 1,2-ethanediol molecules present in the 5OLN chain A used here; the complete deposited 5OLN structure additionally contains a sulfate ion in chain B) were removed before all molecular dynamics simulations, leaving the binding subsite unoccupied prior to ligand placement.

To examine whether unrestrained energy minimization of the semi-open 5EP8 structure would relax it toward a closed-like conformation, 5EP8 min was additionally generated by unrestrained all-atom minimization of 5EP8 in UCSF Chimera^9^ (steepest descent followed by conjugate gradient, 1000 steps each, AMBER ff14SB charges, no positional restraints). This minimization produced only a small backbone displacement from 5EP8 (Cα RMSD 1.72 Å overall and 0.70 Å across the seven active-site residues); that is, it contracted the pocket locally without driving a large semi-open-to-closed backbone rearrangement. 5EP8min is therefore best regarded as an energy-minimized, pocket-contracted model rather than an independently equilibrated closed-state conformation. During the subsequent MD, it retained this contracted character rather than relaxing back toward its semi-open 5EP8 parent: its entrance opening (the mean Tyr165-Leu114 and Tyr165-Gln153 Cα distance) remained significantly narrower than 5EP8orig (trajectory-level median 17.6 vs 18.8 Å; Mann–Whitney *p* = 2.6 × 10^–5^), and its bound-pose fraction (74%, Table S12) matched the closed 1BRW (77%) rather than the semi-open 5EP8orig (59%). Its assignment to the closed-state group is nonetheless deliberately conservative for the Tyr165 lid analysis: because its Tyr165–ligand contact is low, comparable to the open-state structures (Section 3.4), including 5EP8min lowers rather than inflates the closed-group mean. The sulfate ion present in 5EP8 was removed before all subsequent calculations (pocket volume and MD) to ensure that the binding subsite was unoccupied prior to substrate placement. Hydrogens were added in the protonation states predicted by PROPKA3 ^10^ at pH 7.4 (His82 was modeled as the Nε2-H tautomer, HIE; all other histidines were left neutral). This single protonation state was used throughout. The expression “HIS82/HIE82” in the legend of Figure S2 refers to the same Nε2-protonated residue.

Throughout this study, the four structures are labeled by their dominant character: 1BRW (closed state), 5EP8min (closed-like state), 5EP8orig (semi-open state), and 5OLN (open state).

### 2.2 Definition of the Active Site

Seven residues located within the substrate-binding pocket and conserved across the four structures were selected to define the substrate-binding site: Asp80, Lys81, His82, Lys108, Gln153, Tyr165, and Lys188 (PyNP numbering). These residues have the following published roles.^3,4,11^ Lys81 and Lys108 participate in phosphate binding. His82 is a catalytic residue located near the pyrimidine and ribose rings. Tyr165 contacts the pyrimidine base and lies on the gate side of the active-site pocket. Lys188 electrostatically stabilizes the high-energy substrate conformation and contributes to catalysis. Gln153 forms part of the pocket side wall. Residue selection was based on (i) sequence conservation across PyNP and homologous nucleoside phosphorylases, (ii) proximity (<5 Å) to the bound ligand in 1BRW, and (iii) the presence of a resolvable side-chain conformation in all four crystal structures. The geometric center of the seven Cα atoms (ActiveCA7) was used as the reference point for all distance calculations.

### 2.3 Sphere-Packing Pocket-Volume Algorithm

To quantify the volume of the substrate-binding pocket of each crystal structure on a common geometric basis, an in-house sphere-packing algorithm (v6; all parameters in Table S1) was implemented. Cavities were grown by breadth-first expansion of probe spheres of variable radius (1.4–4.5 Å) from multiple anchor seeds: the centroid of the Tyr165 region, together with six seeds offset 4 Å inward from Asp80, Lys81, His82, Lys108, Gln153, and Lys188. A candidate sphere was accepted only when it satisfied all of the following four conditions. (i) It did not overlap any protein atom (van der Waals radii taken from AMBER ff14SB). (ii) It was connected to an already-accepted sphere by a line of sight that did not pass through the protein (breadth-first line-of-sight rule). (iii) It was sufficiently buried (≥40% of its surface facing the protein) and had an acceptable local environment (neighboring residues ≤85% polar-plus-charged and ≥10% hydrophobic). (iv) It made contact with at least one of the catalytic residues {Asp80, His82, Lys108, Gln153, Tyr165, Lys188}. Lys81, although part of the seven-residue active-site definition and used as an anchor seed, was not included in this per-sphere required-contact filter. The procedure terminated when no further candidate satisfying all criteria remained.

The pocket volume was then obtained by volume integration on a cubic grid with a 0.7 Å spacing: *V* = N_cells × (0.7 Å),^3^ where N_cells is the number of grid cells inside the union of the accepted spheres and outside all protein van der Waals spheres (that is, the number of voxels in the carved-out cavity, not the number of fixed-radius spheres). All parameter values are listed in Table S1. As an independent control, the pocket volume was also estimated with fpocket 4.0.^12^ The two algorithms agreed only on coarse trends: both identified the open state (5OLN) as the largest pocket and gave an approximately 1.4–1.7-fold expansion relative to the closed 1BRW (sphere packing v6 1.4-fold, fpocket 1.7-fold). However, the detailed four-structure ranking differed (Spearman ρ = 0.4 between fpocket and the in-house v6 volumes; the previously reported ρ = 1.0 agreement held only between two settings of the same in-house algorithm, v1 and v6, and was therefore not an independent cross-validation). Only the method-independent observations—that 5OLN has the largest active-site pocket and that the pocket expands from the closed to the open state—were therefore regarded as robust, whereas the absolute volumes and the fine ordering of the more closed structures were treated as method-dependent (Table S1). We used the sphere-packing volume as the primary metric because fpocket, which is designed to detect and rank surface-exposed druggable pockets, resolved the deep active-site cavity of PyNP poorly: it compressed the three non-fully-open states into a narrow 1041–1102 Å^3^ band and did not reproduce their expected ordering. The geometry-based sphere-packing algorithm, anchored to the seven conserved active-site residues, instead yielded the graded closed → open volume ladder used in Table 1, with fpocket and two Cα–Cα opening markers retained as independent checks on the end points (Section S1).

**Table 1:** Cα–Cα Distances from the Tyr165 Cα to the Two Neighboring Residues (Leu114 and Gln153), Which Serve as the Basis for the Open/Closed Classification

structure	state	① 165–114 Cα (Å)	② 165–153 Cα (Å)	closure (%)	pocket volume, v6 (Å^3^)
1BRW	closed	12.82	13.94	100	1851.2
5EP8min	closed-like	13.69	15.23	77	1423.8
5EP8orig	semi-open	15.01	16.95	44	1945.2
5OLN	open	16.98	19.06	0	2549.2

### 2.4 Ligand Selection and Parameterization

Thirteen compounds were studied: ten nucleoside analogues and three mechanism-based inhibitors. The nucleoside analogues were each of five purine and purine-like bases—hypoxanthine (H), 6-monomethylaminopurine (mmap), 6-dimethylaminopurine (dmap), indole (ind), and 6-dibutylaminopurine (dbap)—glycosylated with either ribose (-rR) or 2′-deoxyribose (-dR). The three inhibitors were formycin B (a C-nucleoside transition-state mimic), TPI (tipiracil hydrochloride; a thymidine phosphorylase inhibitor), and PIA (5-(phenylthio)­acyclouridine; a uridine phosphorylase (UrdPase) inhibitor).^13^ The five matched rR/dR pairs allow sugar selectivity to be evaluated directly. For the indole (ind) analogue, the five-membered ring of the base is a pyrrole ring rather than the imidazole ring of a standard purine; the base-exchange (phosphorolysis) reaction therefore proceeds only minimally, and ind was included here as an essentially non-reactive binding probe.

Ligand geometries were optimized with the Universal Force Field (UFF) as implemented in Avogadro.^14^ RESP charges^15^ were then derived from the HF/6-31G­(d) electrostatic potential and fitted through ANTECHAMBER.^16^ GAFF2 parameters^17^ were assigned with acpype^18^ to generate GROMACS-compatible^19^ topologies. Each ligand was first docked into each protein structure with AutoDock Vina 1.2.5 ^20,21^ (exhaustiveness 32, 20 modes per run), and the top-ranked pose with the ligand center of mass within 6 Å of the ActiveCA7 centroid was retained as the MD starting structure.

### 2.5 Molecular Dynamics Simulations

All MD simulations were performed with CUDA-accelerated GROMACS. The initial 156 trajectories used GROMACS 2025.2. The phosphate-control, mutant, and 300 ns extension trajectories added for the ACS Omega version used GROMACS 2025.4 on the RCCS and GROMACS 2026.0 on vast.ai cloud GPUs. In the phosphate control, a single HPO_4_^2–^ ion was placed near the Lys81/Lys108/Lys188 subsite of the already-formed enzyme–ligand complexes; by construction, this control does not assemble a Michaelis complex and does not test catalytic turnover, and it was used only to probe the equilibrium geometry of the phosphate relative to C1′ (Section 3.5). The production settings (force field, restraints, PME, and integration time step) were kept identical across all versions. The AMBER ff99SB-ILDN force field^22^ was used for the protein, TIP3P^23^ for water, and GAFF2 for the ligands. The ff99SB-ILDN parameter set was chosen because it remains one of the most extensively validated and widely benchmarked AMBER protein force fields for explicit-solvent dynamics, with well-characterized backbone and side-chain behavior; using an established parameter set makes the present trajectories directly comparable with the large body of published PyNP and nucleoside-phosphorylase simulations and avoids confounding conformational sampling with force-field novelty. The brief Chimera minimization used to build 5EP8 min (Section 2.1) employed the ff14SB charge set that is built into UCSF Chimera; because that step was only a short, restraint-free geometry relaxation used to generate a starting structure—not a source of any reported energetic or dynamical observable—the small difference between the two parameter generations does not affect the production results, all of which were computed from the ff99SB-ILDN trajectories under identical settings. Each system was solvated in a cubic box with a clearance of at least 1.2 nm from the protein, neutralized with Na^+^/Cl^–^ ions, and adjusted to a salt concentration of 0.15 M (per-system compositions in Table S9). Energy minimization (5000 steps, steepest descent) was followed by 100 ps of *NVT* equilibration (Berendsen thermostat,^24^ 300 K) and 100 ps of *NPT* equilibration (Parrinello–Rahman barostat,^25^ 1 atm). Production simulations were run under *NPT* conditions at 300 K with a velocity-rescaling (V-rescale) thermostat and a C-rescale barostat (1 atm), a 2 fs integration time step, LINCS constraints^26^ on hydrogen-containing bonds, and Particle Mesh Ewald electrostatics^27^ (1.2 nm cutoff). The two 100 ps equilibration stages are relatively short, but each production trajectory was 100 ns, and all reported metrics used the later 50–100 ns window after additional relaxation.

Each ligand–structure combination was simulated in three independent replicas (rep1, rep2, rep3) of 100 ns. Each replica was generated by reseeding the initial velocities from an independent random-number generator. For 1BRW, 5EP8 min, and 5OLN, all three replicas were generated on the RCCS supercomputer (Section 2.9). For 5EP8orig, rep1 was generated on RCCS and replicas 2 and 3 on a separate computing platform (vast.ai cloud GPUs). These cross-platform replicas were used both to complete the triplicate design and to check the consistency of the main findings (Section 3.3). The full panel comprised 156 production trajectories (13 ligands × 4 structures × 3 replicas), amounting to 15.6 μs of simulation time. Trajectories were saved at 50 ps intervals (2001 frames per 100 ns).

### 2.6 Analysis Metrics

Each trajectory was characterized by the following three complementary metrics.

(i) Binding fraction (BF < 10 Å). The center of mass of the Cα atoms of the seven active-site residues (ActiveCA7) and the center of mass of all ligand heavy atoms were computed per frame with gmx distance. The binding fraction is the fraction (%) of the full 0–100 ns simulation in which this COM–COM distance was ≤1.0 nm (10 Å), and it measures the time the ligand remained within the active-site region. The 10 Å threshold corresponds approximately to the sum of the radial extent of the ligand (about 5 Å) and that of the seven-residue centroid (about 5 Å). Robustness was verified by recomputing BF at 8 Å and 12 Å (Figure S3). The two binding metrics are distinguished throughout and are always written with their thresholds: BF < 10 Å (this coarse COM–COM occupancy of the active-site region) and BF_contact < 4 Å (the finer per-residue atom–atom contact fraction defined below); the two are not interchangeable.
(ii) Minimum atom–atom distance (mindist). The closest distance between any ligand heavy atom and any atom of the seven active-site residues was computed per frame with gmx mindist. The median over the later equilibration window (50–100 ns) is reported; it captures direct van der Waals/hydrogen-bond contact and is insensitive to the choice of binding-fraction threshold. Because the per-trajectory mindist distribution is bimodal (bound poses at about 2.5 Å, dissociated poses at 15–40 Å), the median—rather than the mean, which is readily pulled up by dissociated frames—was used as the summary statistic throughout, including when aggregating across replicas and conformational groups.
(iii) Per-residue contact frequency. For each of the seven active-site residues, atom distances to predefined ligand functional groups were tabulated, and the contact frequency was defined as the fraction of frames with a distance <4 Å (the standard van der Waals contact cutoff).

The following additional analyses were applied to all systems. First, the ligand RMSD was computed as follows: minimum-image correction was applied at each frame, Kabsch alignment was performed on the Cα atoms of the seven active-site residues, and the heavy-atom RMSD relative to the initial docked pose was computed and reported for all 156 systems. The distance-based metrics (BF and mindist) are also computed within a single periodic image and are therefore unaffected by periodic boundaries. Second, all metrics were averaged over the three replicas and reported. Third, the ligand state was classified on the basis of the later-window mindist as Bound (median mindist <0.5 nm), Loose (0.5–1.5 nm), or Dissociated (≥1.5 nm).

### 2.7 Pattern Classification

To examine compound-specific structural preferences, each ligand was classified into a pattern on the basis of the structure with the highest BF: pattern A = 5EP8 min-optimal, pattern B = 1BRW-optimal, pattern C = 5EP8orig-optimal, pattern D = 5OLN-optimal. To avoid over-interpreting small differences, a ligand was assigned to a pattern only when the best structure exceeded the second-best by ≥ 10 percentage points in BF; otherwise, the ligand was labeled “mixed”. Sensitivity to the 10% margin was tested with margins of 5% and 15% (Table S10).

### 2.8 Statistical Analysis and MM-PB­(GB)­SA

For the closed-versus-open comparison, 1BRW and 5EP8min were grouped as closed and 5EP8orig and 5OLN as open. In this paper, the phrase “volume order (5EP8min <1BRW < 5EP8orig <5OLN)” denotes the ranking of pocket volumes and is distinguished from the functional closed/open classification; “interior” is used only for the intermediate structures that do not lie at either end of this volume order. Distributions were compared with the Mann–Whitney *U* test, and significance was denoted as ***p < 0.001, **p < 0.01, *p < 0.05, and ns *p* ≥ 0.05. Given the number of residue-level comparisons (seven residues across two window definitions and two grouping schemes), the per-residue *p* values are read as descriptive. Benjamini–Hochberg correction across the seven residues leaves Tyr165 (and, by p value alone, His82 and Asp80) below a 5% false-discovery rate, but Asp80 is rejected on effect-size grounds (Δ = +0.1 pp). The conclusions are therefore based not on any single uncorrected threshold but on effect size and on the agreement between the trajectory-level and compound-level analyses (Table 3). Because the three replicas of each compound–structure system are not mutually independent, and the 13 ligands are also grouped into a few compound classes (rR series, dR series, inhibitors), the number of truly independent samples is smaller than it appears. Treating all trajectories as independent samples therefore overestimates statistical significance. All closed-versus-open residue contrasts were accordingly evaluated with two additional structure-respecting procedures: a ligand-level Wilcoxon signed-rank test (*n* = 13 compounds, each contributing one closed-group mean and one open-group mean) and a linear mixed-effects model with compound as a random intercept (BF ∼ group + (1 | compound)). Results of all three procedures are reported in Table S4. Throughout the text, n is stated explicitly as either the number of trajectories or the number of independent compounds. Effect sizes are reported as differences of group means. A Pearson correlation was computed between the later-window binding fraction (BF < 10 Å) and the later-window median mindist. Across all 156 trajectories, the two metrics were significantly negatively correlated (*r* = −0.34, *p* = 1.5 × 10^–6^); however, when aggregated to the 13 compounds, this correlation was no longer significant (*r* = −0.25, *p* = 0.41), indicating that part of the trajectory-level correlation arises from the similarity among replicas of the same compound and should be interpreted accordingly.

**Table 2:** Pattern Classification of the 13 Ligands by Replica-Averaged BF_contact <4 Å^a^

	BF_contact <4 Å (%)						
compounds	1BRW	5EP8min	5EP8orig	5OLN	best	Δ (pp)	pattern
H-rR	51.5	59.2	77.5	16.5	5EP8orig	18.3	C
H-dR	89.1	72.0	50.9	49.4	1BRW	17.1	B
dmap-rR	71.3	93.9	97.1	38.3	5EP8orig	3.2	mixed
dmap-dR	77.1	55.9	50.2	58.7	1BRW	18.4	B
mmap-rR	97.1	86.9	72.7	80.3	1BRW	10.2	B
mmap-dR	76.6	57.6	90.2	65.4	5EP8orig	13.6	C
ind-rR	67.9	62.4	14.8	28.7	1BRW	5.5	mixed
ind-dR	48.4	72.8	81.2	31.7	5EP8orig	8.4	mixed
dbap-rR	78.7	72.0	97.8	62.7	5EP8orig	19.1	C
dbap-dR	97.6	82.0	92.0	72.3	1BRW	5.6	mixed
Formycin B	91.4	100.0	23.7	69.4	5EP8 min	8.6	mixed
TPI	76.3	17.9	19.9	27.3	1BRW	49.0	B
PIA	100.0	77.4	81.8	92.6	1BRW	7.4	mixed

a Δ is the margin (percentage points) relative to the second-best structure. Compounds with Δ ≥ 10 receive a pattern label; smaller margins are “mixed”. Note: the “mixed” label reflects only a small best-versus-second margin (the compound binds well to several structures); it does not indicate weak or non-reproducible binding. dmap-rR and dbap-rR are “mixed” under this margin rule but bind reproducibly to 5EP8orig (> 95 % in all replicas) and show a robust ribose preference within 5EP8orig (Sections 3.3 and 3.4).

**Table 3:** Per-Residue Contact Fractions by Conformational Group (BF_contact <4 Å, %) and Mann–Whitney Comparisons^a^

residue	closed (%)	open (%)	Δ (pp)	*P* value	significance
Asp80	2.2 ± 1.3	2.1 ± 0.8	+0.1	0.008	** §
Lys81	11.3 ± 2.7	15.6 ± 2.4	–4.3	0.074	ns
His82	36.7 ± 3.6	25.5 ± 3.0	+11.2	0.011	*
Lys108	14.3 ± 3.0	15.3 ± 2.5	–1.0	0.430	ns
Gln153	23.8 ± 3.3	21.7 ± 3.4	+2.1	0.213	ns
Tyr165	42.5 ± 3.6	21.2 ± 3.0	+21.3	1.2 × 10^–5^	***
Lys188	15.3 ± 3.2	15.2 ± 2.8	+0.1	0.344	ns

a Values are mean ± SEM; Δ (pp) = closed mean – open mean. § For Asp80, the nominal significance reflects the very low, narrowly distributed contact values; the effect size is negligible (Δ = +0.1 pp) and is not interpreted as a conformational signal.

Binding free energies were estimated with MM-PB­(GB)­SA (gmx_MMPBSA v1.6.4)^28^ over the equilibrated later half of each trajectory (frames 1001–2000, stride 5). For each trajectory, PBC correction was applied before energy decomposition with a two-step gmx trjconv procedure (-pbc nojump → -pbc mol -ur compact -center). The generalized-Born end point ΔTOTAL_GB (enthalpic, entropy-uncorrected) was used as the primary MM-PBSA descriptor throughout. The interaction-entropy (IE) correction was evaluated for all 153 completed Phase C systems^29^ but was not used in any main-text figure or claim because its reliability indicator σ­(IE) exceeded the 3.6 kcal/mol threshold^30^ in 152/153 systems (mean σ = 13.14 kcal/mol). The IE column is retained in the Supporting Information with an “IE unreliable” flag for completeness (Figure S2).

### 2.9 Software and Code Availability

Simulations were run on the supercomputer of the Research Center for Computational Science (RCCS), Okazaki Research Facilities, National Institutes of Natural Sciences, Okazaki, Japan, and on vast.ai cloud GPUs (5EP8orig rep2/rep3). Analyses were carried out with the native tools of GROMACS 2025.2, Python 3.12 (NumPy 2.0, SciPy 1.13, pandas 2.2, Matplotlib 3.9, and openpyxl 3.1), and UCSF Chimera 1.18. The sphere-packing pocket-volume algorithm, all analysis scripts, and the aggregated CSV outputs are provided as Supporting Information (see the Data and Software Availability statement). To enable independent verification, all 156 raw trajectories were deposited in a public repository (Zenodo) as periodic-boundary-corrected, solvent-stripped versions (protein plus ligand, about 67 MB each), under 10.5281/zenodo.21448920; full versions including water molecules are also provided for the main systems. The full set of analysis scripts, including the index.ndx generation command, has also been released.

## 3 Results

### 3.1 The Active-Site Pocket Volume Varies Markedly among the Conformational States

To establish the structural framework for the subsequent dynamics analysis, the geometry of the substrate-binding pocket was first quantified for the four conformational states of PyNP (Figure 2). The four states were as follows: (i) 1BRW is a holo structure crystallized with uracil and phosphate (the products of phosphorolysis) and was assigned to the closed state. (ii) 5EP8 min is an energy-minimized model derived from 5EP8, in which the structure had relaxed toward a closed-like conformation. (iii) 5EP8orig is the X-ray structure 5EP8 as deposited, in which the backbone is partially open and a sulfate ion occupies the phosphate-binding subsite (semi-open conformation). (iv) 5OLN was crystallized with imidazole and 1,2-ethanediol and represents the fully open conformation. The sulfate ion in 5EP8orig was retained only for the pocket-volume calculation of the crystal structure itself and was excluded from all molecular dynamics systems (Methods Section 2.1).

**Figure 2:**
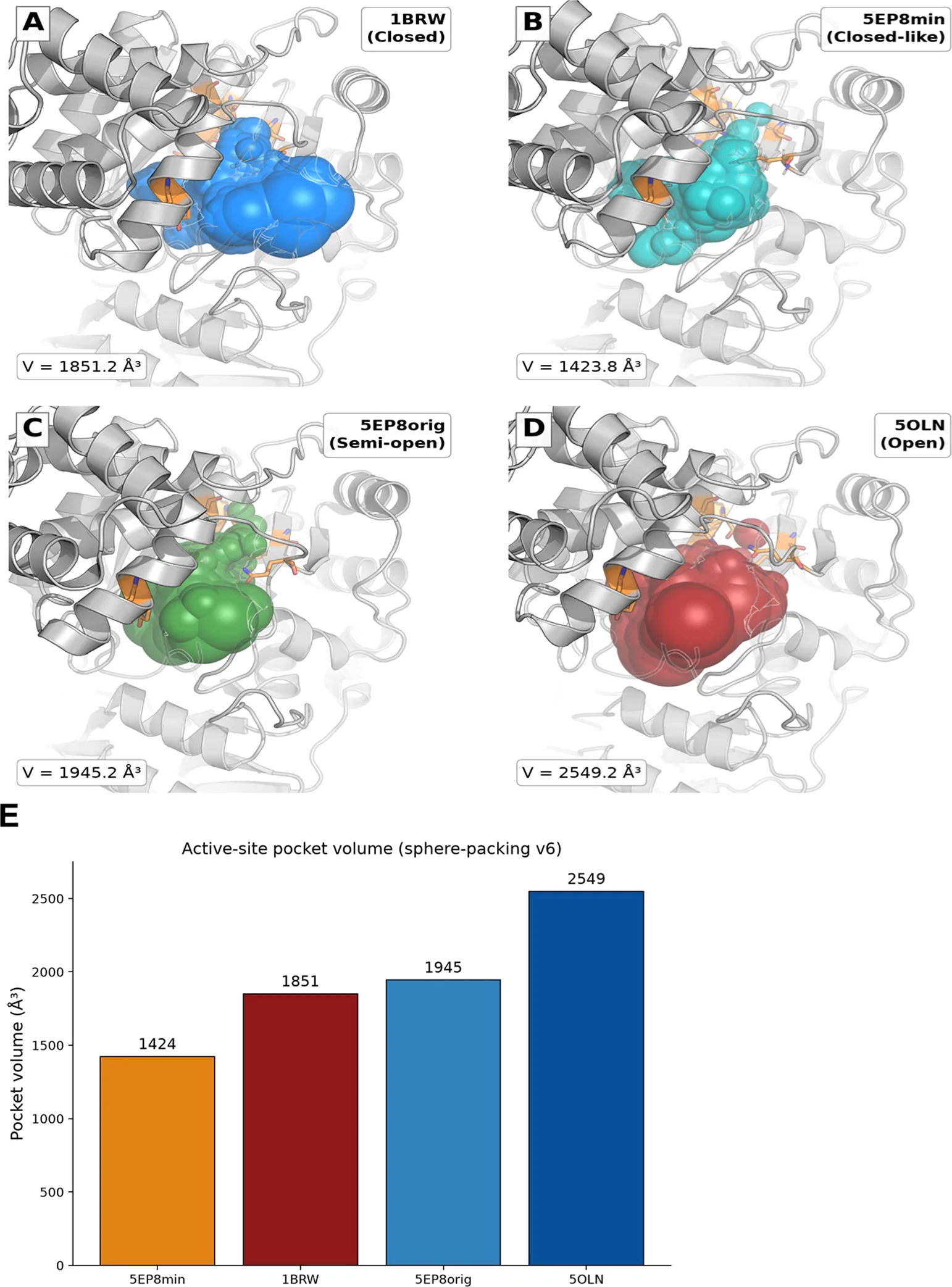
Active-site pocket of PyNP across four conformational states. Cartoon (gray, gate loop 153–170 colored per state); spheres = pocket envelope (sphere-packing v6 reference); orange sticks = seven active-site residues (D80, K81, H82, K108, Q153, Y165, K188). Pocket volumes (V) and sphere counts (N) are from Table 1. Panels (A) 1BRW, closed (V = 1851.2 Å^3^); (B) 5EP8min, closed-like (V = 1423.8 Å^3^); (C) 5EP8orig, semi-open (V = 1945.2 Å^3^); (D) 5OLN, open (V = 2549.2 Å^3^). (E) Bar chart of the active-site pocket volume across the four structures (sphere-packing v6). Each cavity was tiled to the algorithm’s 220-sphere cap. All four structures are superimposed onto 1BRW and viewed from the same angle.

The pocket volume was determined with the in-house sphere-packing algorithm (Methods Section 2.3). This algorithm iteratively places non-overlapping probe spheres within the cavity defined by the Cα atoms of the seven active-site residues (Asp80, Lys81, His82, Lys108, Gln153, Tyr165, Lys188). The resulting pocket envelope is physically connected, anchored to the catalytic residues, and large enough to accommodate a typical pyrimidine nucleoside (about 200 Å^3^). The algorithm parameters and the validation against an independent fpocket calculation are given in Section S1. The four pocket volumes are summarized in Table S1 and shown in Figure 2. Three points emerged.

First, the interior volume of the two closed states (1BRW, 5EP8min) was around 1400–1900 Å^3^, consistent with the space required to accommodate one nucleoside molecule and the co-substrate phosphate simultaneously. Second, the two open-side states were larger, 1945 Å^3^ (5EP8orig) and 2549 Å^3^ (5OLN), corresponding to an approximately 1.4-fold expansion of the fully open 5OLN relative to the closed 1BRW. Because the absolute volumes depend on the sphere-packing measurement method, the absolute values themselves are not emphasized; the ranking of the structures is the robust result (Table S1). Third, the interior volume increased in the order 5EP8min <1BRW < 5EP8orig <5OLN and can be interpreted as a structural transition from the most contracted (substrate-trapping) state to the most expanded (substrate-accessible) state. The volume of 5EP8min was slightly smaller than that of 1BRW, indicating that energy-minimizing the 5EP8 backbone toward the closed family produced a somewhat under-relaxed (over-contracted) pocket; nevertheless, its value remained close to the interior volume of the genuine closed state, 1BRW.

Across the four structures, the volume increased in the order given above. The semi-open 5EP8orig (1945 Å^3^) fell between the closed 1BRW (1851 Å^3^) and the fully open 5OLN (2549 Å^3^), consistent with an intermediate degree of opening rather than a fully open cavity. The two crystallographic states differ in their bound small molecules—5EP8orig carries a sulfate ion in the phosphate subsite, whereas 5OLN was crystallized with imidazole and 1,2-ethanediol in chain A used here (the complete deposited structure additionally contains a sulfate ion in chain B). This result showed that the opening and closing of the pocket is determined by backbone conformational change rather than by the presence or absence of small anions such as sulfate or phosphate and that the open state captured by 5OLN is not an artifact arising from the absence of a phosphate analogue. To confirm this, the Cα distance was measured between Tyr165—which lies on the gate side of the active-site pocket and makes contact with the pyrimidine base^3,4^ —and Gln153, which forms part of the pocket side wall. The Y165–Q153 Cα distance increased monotonically across the four conformations (13.94 Å (1BRW) < 15.23 Å (5EP8min) < 16.95 Å (5EP8orig) < 19.06 Å (5OLN); total opening from closed to open +5.12 Å), reproducing the same order inferred from the pocket volume. A second pair spanning the active site, Lys108–Tyr165, widened in parallel (20.39 → 23.68 Å, +3.29 Å), as did Lys108–Lys188 (8.95 → 11.19 Å, +2.24 Å). This showed that the expansion is not a uniform swelling but is concentrated in the loop region bearing Tyr165 (the α7−α8 loop). The agreement of the two independent geometric measures—the sphere-packing pocket volume and the active-site Cα–Cα distances—supports the assigned conformational states and provides the structural baseline for the molecular dynamics below.

As the basis for the open/closed classification, we focused on the “gate” structure in which the Tyr165 side chain divides the entrance of the active site into two openings and recomputed the closure % (the mean of the two openings) by measuring the Cα distances from Tyr165 Cα to those of the two neighboring residues (Leu114 and Gln153). Both openings expanded monotonically from 1BRW (the most closed state) to 5OLN (the most open state) (opening 1 full range 4.16 Å; opening 2 full range 5.12 Å, the latter matching the Y165–Q153 opening of +5.12 Å noted above). The closure % is a relative index defined as closure % = (d_5OLN – d)/(d_5OLN – d_1BRW) × 100 (normalized by the two-opening mean; 1BRW = 100%, 5OLN = 0%). The closure % (two-opening mean) was 100/77/44/0 (1BRW = 100, 5EP8min = 77, 5EP8orig = 44, 5OLN = 0; Table 1), consistent in order, monotonicity, and full range.

In summary, the active-site pocket of PyNP expands approximately 1.4-fold between the closed state (1BRW) and the open state (5OLN), with 5EP8orig at an intermediate volume and 5EP8min slightly more contracted than that of 1BRW. This conformational heterogeneity is consistent with an induced-fit mechanism in which substrate binding stabilizes the closed state, and it motivates the question addressed next: Can the larger open pocket retain a substrate as effectively as the smaller closed pocket?

### 3.2 The Closed Conformation Retains the Substrate More Persistently Than the Open Conformation

Having established the hierarchy of pocket volumes, we examined whether the larger open pocket retains a bound ligand as effectively as the smaller closed pocket. Across the full panel of 156 trajectories (13 ligands × 4 structures × 3 replicas), each trajectory was assigned a binding state from the minimum ligand–active-site distance over the later window (50–100 ns). The states were Bound (median mindist <0.5 nm), Loose (0.5–1.5 nm), and Dissociated (≥1.5 nm) (Figure 3, Methods Section 2.6; Figure 3 panels A–C, Table 3; the complete per-trajectory binding states are listed in Table S2).

**Figure 3:**
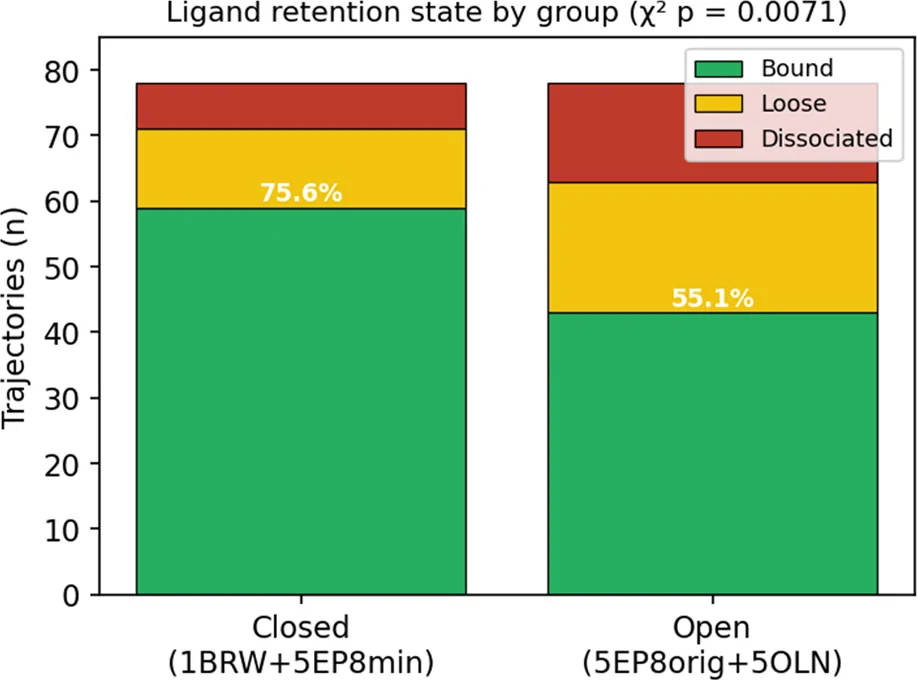
Fraction of substrate binding states in the open/closed groups of each enzyme. Ligand binding-state composition (Bound/Loose/Dissociated) and the bimodality of the later-window minimum distance. The closed group is Bound more frequently than the open group (75.6% vs 55.1%, χ^2^ *p* = 0.0071; compound-level paired Wilcoxon p = 0.018). Panels A–C show the state composition of each conformational group. The full closed-versus-open comparison gave a compound-level *p* = 0.002 (BF_contact <4 Å).

The closed conformation retained ligands in the Bound state at a clearly higher frequency than the open conformation. Grouping 1BRW and 5EP8min as closed and 5EP8orig and 5OLN as open, 75.6% (59/78) of the closed-group trajectories were classified as Bound, versus 55.1% (43/78) for the open group (χ^2^ = 7.25, *p* = 0.0071; Yates-corrected *p* = 0.012). This shift was accompanied by redistribution into the Loose and Dissociated categories: the open group contained roughly twice as many Dissociated trajectories as the closed group (15 vs 7) and more Loose trajectories (20 vs 12). Because this χ^2^ test treats trajectories as independent, the effect was also confirmed with independent compounds: the per-compound binding fraction was higher in the closed group (paired Wilcoxon across 13 compounds, *p* = 0.018). Thus, the higher retention in the closed state is supported at the compound level even in the present (100 ns) analysis, and the 300 ns extension of the central systems preserved the same retention ranking (Section 4.6). As with the Tyr165 contrast, part of this signal was contributed by 1BRW: even with 1BRW excluded, the closed-like model 5EP8 min alone still showed a higher BF_contact than the open group (70.0% vs 57.4%). However, this single-structure comparison is only marginally significant (Mann–Whitney *p* = 0.063; compound-level paired Wilcoxon *p* = 0.080). The full closed-versus-open comparison (compound-level *p* = 0.002, BF_contact <4 Å) is therefore presented as a provisional result based on the agreement between the trajectory level (χ^2^ *p* = 0.0071) and the compound level, and we do not claim that retention is independent of 1BRW.

The continuous minimum-distance metric over the later window showed the same trend. The later-window mindist distribution is bimodal: bound poses cluster near 2.5 Å, and dissociated poses lie at 15–40 Å; it was therefore summarized by the median, which is less sensitive to the dissociated tail (Methods Section 2.6). The closed group sat closer to the active site (later-window mindist median 2.68 Å) than the open group (3.94 Å) (one-sided Mann–Whitney *U* test, closed < open, *p* = 0.017). The structure-resolved later-window mindist median followed the same monotonic ladder as the pocket volume in Section 3.1 (1BRW 2.62 Å < 5EP8min 2.88 Å < 5EP8orig 3.30 Å < 5OLN 4.54 Å); that is, as the pocket expanded, the ligand sat progressively farther from the active site. The two metrics were significantly negatively correlated across the panel: the higher the later-window binding fraction (BF < 10 Å), the smaller the later-window minimum distance (Pearson *r* = −0.34, *p* = 1.5 × 10^–6^, *n* = 156). This confirms that the COM-based binding fraction and the inter-atomic minimum distance capture the same underlying engagement from complementary perspectives and that neither is an artifact of the other. Taken together, the conformational state governed how persistently the substrate analogues were retained: the closed conformation kept ligands in direct contact with the active site for a larger fraction of the simulation time and held them closer to the catalytic residues than the open conformation. Notably, this proximity ladder was monotonic with respect to pocket closure (closest for 1BRW, farthest for 5OLN). This pattern contrasts with the ranking of the MM-PB­(GB)­SA binding energies presented in Section 4.2, which tends toward a non-monotonic profile that apparently favors the interior conformations but remains statistically unresolved once replica error is taken into account. These overall results are summarized in Figure 3.

At each frame, the ligand was minimum-image-corrected to the nearest image of the pocket centroid, Kabsch-aligned on the Cα atoms of the seven active-site residues, and the ligand heavy-atom RMSD relative to the initial docked pose was computed for all 156 systems. Only 21/156 systems strictly retained the initial pose (later-window median RMSD <5 Å); 94 systems relaxed or reoriented to 5–15 Å, and 41 systems dissociated beyond 15 Å (median RMSD of the retained systems 3.75 Å). The dependence on the initial docked pose is therefore not negligible, and pose-dependent conclusions are stated cautiously. On the other hand, the extent of dissociation followed the structural gradient: the number of dissociated systems was 1BRW 5 < 5EP8min 10 < 5EP8orig 12 < 5OLN 14, mechanistically consistent with this section’s trend that structures at the closed-state end retain ligands better (pose states from periodic-boundary-corrected RMSD in Figure S8 and Table S12).

To evaluate the convergence of retention at the 100 ns scale per replica, the time series of the minimum distance to the active site and the cumulative binding fraction BF­(*t*) were plotted per system (Figure S5), and the attainment of a plateau in the later window was judged quantitatively. Of 146 systems, 67 (46%) reached a plateau; most of the systems that did not were concentrated among weakly retained ligands (TPI, ind-rR, H-rR, ind-dR, and formycin B). Thus, 100 ns is convergent for strongly retained systems but insufficient for weakly retained ones. The binding fraction and minimum distance metrics of this study are therefore interpreted consistently as measures of dynamic retention on the 100 ns scale rather than of equilibrium affinity.

### 3.3 The Conformational State Modulates Ribose-versus-2′-Deoxyribose Preference and Defines Compound-Specific Binding Patterns

The analysis in Section 3.2 showed that, averaged over the 13 ligands, the closed conformation retains the substrate better than the open conformation. Here, we address two more specific questions. First, did the ribose-versus-deoxyribose distinction at the C2′ position of the sugar interact with the conformational state? Second, if individual compounds preferred particular conformations, how reproducibly could these preferences be assigned, and what fraction of the panel was genuinely structure-selective?

#### 3.3.1 A Pairwise View of Sugar Selectivity

For each of the five matched rR/dR compound pairs (H, dmap, mmap, ind, dbap), ΔBF_contact = BF_contact­(rR) – BF_contact­(dR) was computed independently in each of the four protein structures. The complete 20-entry matrix is shown below (Figure 4). Three observations stood out.

**Figure 4:**
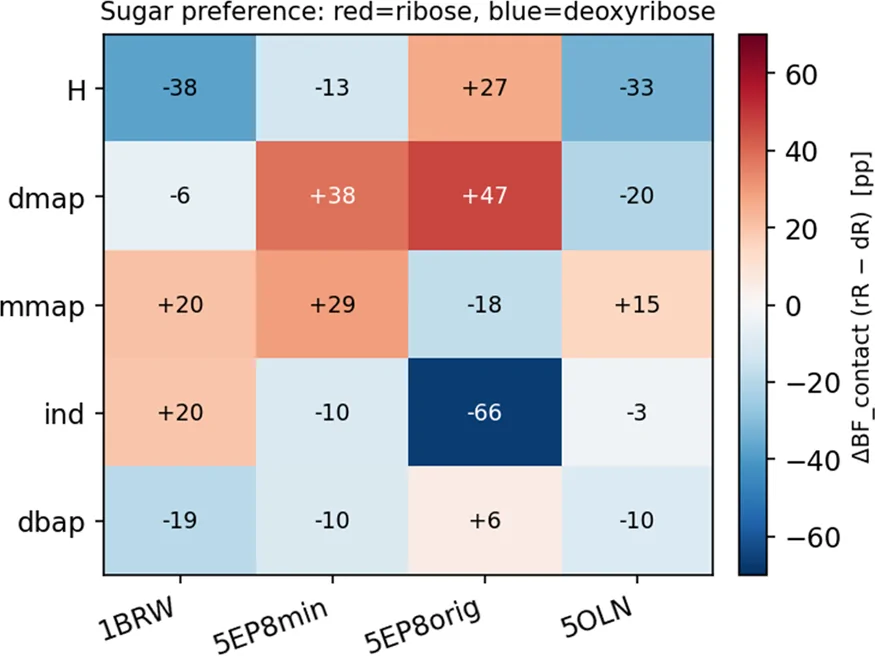
Sugar-preference matrix of the five rR/dR compound pairs (H, dmap, mmap, ind, dbap) × four conformations, ΔBF_contact (rR – dR). The magnitude and sign of the sugar preference depend strongly on the protein conformation, showing that ribose-versus-2′-deoxyribose preference is a compound-by-compound × conformation-by-conformation property.

Observation 1. The magnitude, and even the sign, of the sugar preference depended strongly on the protein conformation. Within the closed structures, the H pair was strongly dR-preferring in 1BRW (ΔBF = −37.6%) and moderately dR-preferring in 5EP8min (−12.8%), whereas the dmap pair was rR-preferring in 5EP8min (+38.0%) but neutral in 1BRW (−5.8%). The open structures reversed this order: the H pair flipped to rR-preferring in 5EP8orig (+26.6%) and returned to dR-preferring in 5OLN (−32.9%), whereas the dmap pair became strongly rR-preferring in 5EP8orig (+46.8%) but dR-preferring in 5OLN (−20.4%). The four conformations thus presented four qualitatively distinct patterns of sugar preference toward the same set of ligands.

Observation 2. The most pronounced single-compound contrast in the panel was observed for the ind pair in 5EP8orig: ΔBF_contact = −66.4% (ind-rR 14.8% vs ind-dR 81.2%), an order of magnitude larger than any other entry and robust across replicas. The largest contrast in the opposite direction was shown by the dmap pair in the same structure, ΔBF_contact = +46.8% (dmap-rR 97.1% vs dmap-dR 50.2%). The 5EP8orig structure therefore sharpened the sugar preference of the bound ligands most strongly, and its direction was opposite for the chemically similar ind and dmap pairs (dR-preferring for ind, rR-preferring for dmap).

Observation 3. The overall sugar preference, averaged across the four structures, showed no statistically significant difference. Paired Wilcoxon comparisons between the rR and dR members of all five pairs gave Δ_mean = −4.5% (1BRW), +6.8% (5EP8min), −0.9% (5EP8orig), and −10.2% (5OLN), none of which was significant (minimum *p* = 0.31, *n* = 5 pairs). The sugar preference in PyNP was therefore not a property of the whole active site but a compound-by-compound and conformation-by-conformation property; averaging across compounds erased the strongly compound-specific signal. This variance-amplification effect is revisited in Section 4.

#### 3.3.2 Pattern Classification

To condense the structural preference of each ligand into a single label, each compound was assigned to a pattern on the basis of the structure giving the highest replica-averaged BF_contact <4 Å, requiring a margin of ≥10 percentage points over the second-best structure (Methods Section 2.7). Patterns A, B, C, and D denote ligands best fitted by 5EP8min, 1BRW, 5EP8orig, and 5OLN, respectively. The classification (Table 2) divided the panel into three robust groups and one large “mixed” group.

The classification assigned four compounds to pattern B (1BRW-preferring: H-dR, dmap-dR, mmap-rR, TPI), three to pattern C (5EP8orig-preferring: H-rR, mmap-dR, dbap-rR), and six to the mixed category (dmap-rR, ind-rR, ind-dR, dbap-dR, formycin B, and PIA). A striking bias here was that patterns A (compounds best fitted by 5EP8min) and D (best fitted by 5OLN) both had no members. Formycin B reached 100% in 5EP8min but exceeded the next-best 1BRW by only 8.6 pp and was classified as mixed for insufficient margin. Moreover, no compound had 5OLN as its best structure at any margin threshold. The panel therefore separated into two preference clusters: compounds optimized for the standard closed state (1BRW, pattern B) and compounds optimized for the intermediate/open 5EP8orig state (pattern C). No compound was optimized for 5EP8min or 5OLN.

#### 3.3.3 Pattern C Was Reproducibly Supported across Replicas and Platforms

The completion of the full triplicate for 5EP8orig (rep1 on RCCS, rep2 and rep3 on vast.ai) allowed the reproducibility of the pattern-C assignment to be cross-checked. Two compounds bound reproducibly and almost saturatingly to the 5EP8orig pocket: dmap-rR (mean 97.1%, >95% in all replicas) and dbap-rR (mean 97.8%, >97% in all replicas) (Figure 5C). However, because both also bind strongly to other structures, the best-versus-second margin fell below the 10 pp pattern threshold, and both were formally classified as “mixed” rather than “pattern C” in Table 2. The robust, reproducible effect of these compounds is their ribose preference within 5EP8orig (for example, dmap-rR exceeds dmap-dR by +46.8 pp; Section 3.4). This is a separate analysis from the cross-structure pattern assignment. For both compounds, 5EP8orig provided almost saturating contact with the active-site residues regardless of replica or computing platform, showing that the assignment did not depend on the chance initial pose of any single trajectory. We treat dmap-rR and dbap-rR as the standard examples of intermediate-state-preferring binding in this dataset and examine the underlying contact mechanism in Section 4.

**Figure 5:**
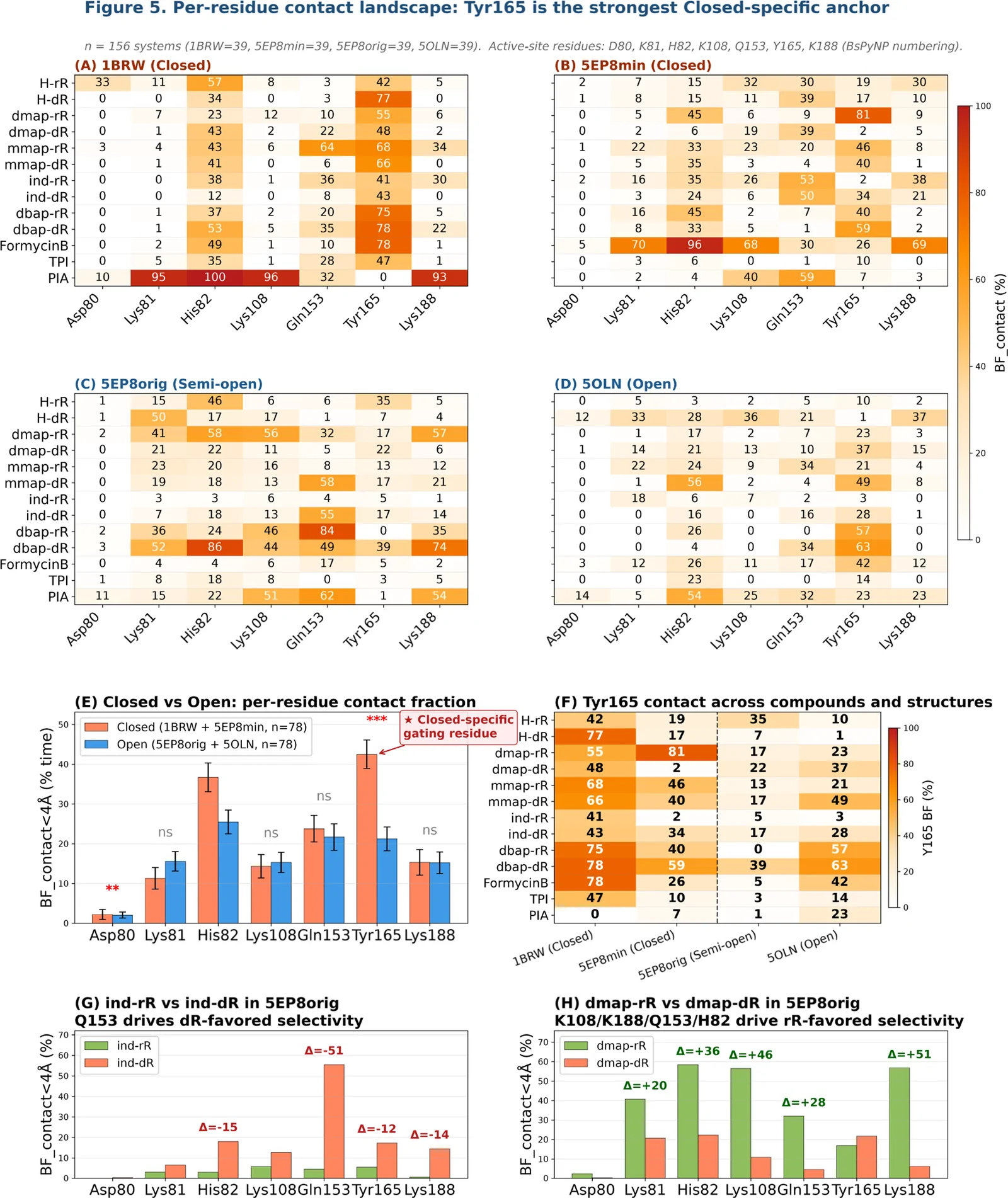
Per-residue contact landscape: Tyr165 is the strongest closed-state-specific anchor. (A–D) BF_contact <4 Å (%) heatmaps of the 13 ligands × 7 active-site residues (Asp80, Lys81, His82, Lys108, Gln153, Tyr165, Lys188) in each of the four conformations (1BRW/5EP8min/5EP8orig/5OLN). (E) Per-residue contact fraction for the closed state (1BRW + 5EP8 min, *n* = 78) versus the open state (5EP8orig +5OLN, *n* = 78); Tyr165 shows the largest difference (***), His82 the next (*), and the other residues are conformation-invariant. (F) Tyr165 contact matrix across all compounds × four structures. (G) Per-residue comparison of ind-rR versus ind-dR in 5EP8orig; Gln153 alone drives the dR preference (Δ = −51 pp). (H) Per-residue comparison of dmap-rR versus dmap-dR in 5EP8orig; a multi-residue network of Lys108, Lys188, Gln153, and His82 drives the rR preference.

#### 3.3.4 Pattern B Compounds Form the Standard Strong-Binder Group

The four pattern-B compounds (H-dR, dmap-dR, mmap-rR, TPI) were characterized by very high contact fractions in 1BRW (76–97%) and substantially lower contact in the most expanded 5OLN (15–80%). TPI showed the most pronounced example: BF_contact = 76.3% in 1BRW, versus 17.9%, 19.9%, and 27.3% in 5EP8min, 5EP8orig, and 5OLN, respectively. Its margin of 49.0 pp was the largest in the panel, consistent with TPI being a clinical-grade thymidine phosphorylase inhibitor designed to recognize the closed, ligand-trapping active site.^31,32^ Separately, H-dR also belonged to pattern B (89.1% in 1BRW vs 50.9% in 5EP8orig). H-dR is a 2′-deoxyribonucleoside whose base is a purine rather than a pyrimidine, but in this panel, it is one of the closest analogues of the natural pyrimidine 2′-deoxyribonucleoside substrate of 1BRW (which was crystallized with its phosphorolysis products, uracil and phosphate). This provides an internal-consistency check supporting the validity of BF_contact as an indicator of the established structure–function relationship.

#### 3.3.5 The Mixed Category Encompasses Two Distinct Mechanisms

The six mixed compounds reflected not a single phenomenon of “binding indiscriminately to all structures” but two distinct mechanisms. The first is non-selective binding: PIA (100/77/82/93%) and dbap-dR (98/82/92/72%) bound at high fractions to all four conformations. In both, the difference (margin) between the best and second-best structures was small (PIA 7 pp, dbap-dR 6 pp), and they bound to residues conserved across the several accessible conformations, functioning as positive controls for the analysis pipeline. The second is marginal preference: dmap-rR (margin 3.2pp), ind-rR (5.5pp), ind-dR (8.4pp), and formycin B (8.6pp) showed a clear conformational preference but fell just below the 10pp threshold. Relaxing the threshold to 5pp reclassified five of these six compounds into their best-fit patterns (ind-dR → C, formycin B → A, and ind-rR, PIA, dbap-dR → B), leaving only dmap-rR (margin 3.2 pp) as mixed; tightening the threshold to 15pp classified only five compounds in total (Table S10). We retained the conservative 10% threshold in the main text but note the underlying trends in Section 4.

#### 3.3.6 Sugar-Ring Puckering Provides the Basis for Ribose/Deoxyribose Discrimination, but the Degree of Discrimination Depends on the 6-Position Substituent

To test whether substrate sugar discrimination appears at the level of the sugar-ring conformation, the pseudorotation phase angle P (Altona–Sundaralingam) of the sugar ring was computed for each trajectory (Figure 6). The ribose series (rR) occupied North (C3′-endo, *P* ≈ 0–36°) on average 64.3% of the time, and the 2′-deoxyribose series (dR) occupied South (C2′-endo, *P* ≈ 144–180°) on average 57.9% of the time, reproducing the known sugar-puckering preferences. In comparison within the same (structure × base) pair, ΔNorth­(rR – dR) was significant (Figure 7; Wilcoxon test p = 0.021; trajectory level p = 8.7 × 10^–5^). Both puckers were adequately sampled, which supports the validity of the force field and sampling used.

**Figure 6:**
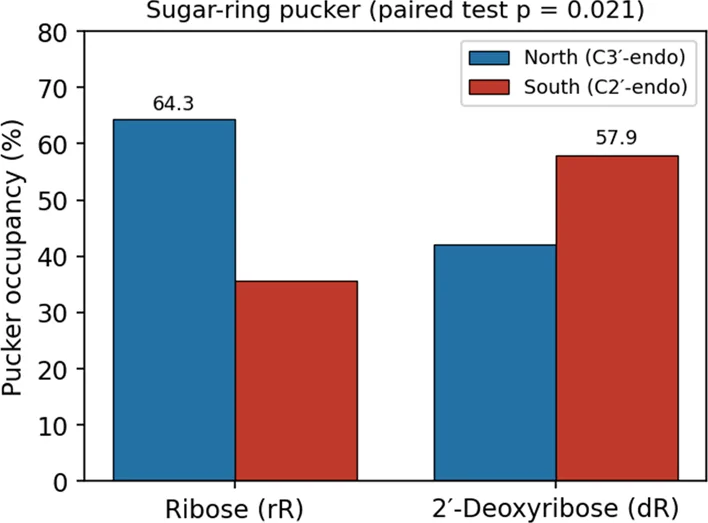
Sugar-ring puckering analysis (pseudorotation phase angle P). Overall, ribose (rR) occupied North (C3′-endo) 64.3% and 2′-deoxyribose (dR) occupied South (C2′-endo) 57.9% (paired test *p* = 0.021). However, the per-sugar difference varied among compounds, being largest for the unsubstituted H and shrinking as the 6-position substituent became larger (per compound in Figure 7).

**Figure 7:**
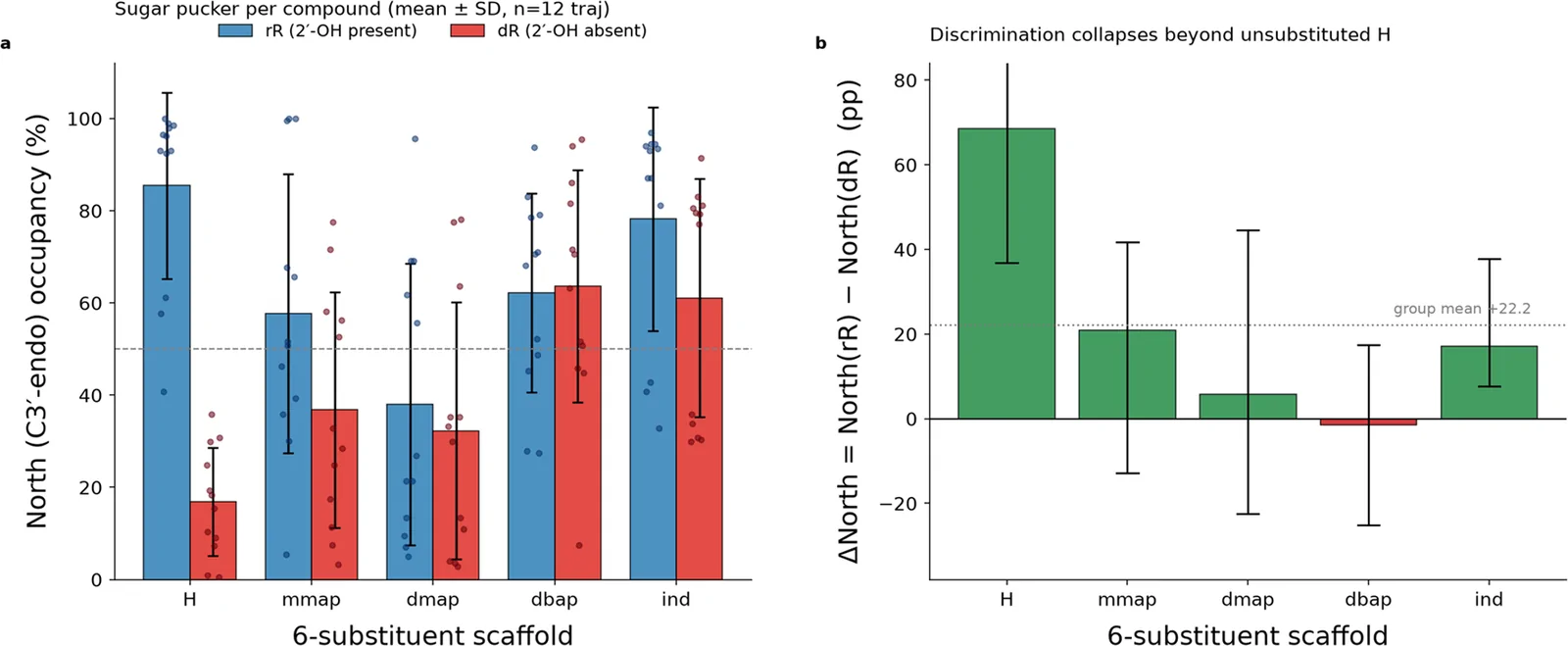
Sugar-ring puckering by compound (North occupancy, rR versus dR). For each 6-position-substituent scaffold, the North (C3′-endo) occupancy of ribose (rR) and 2′-deoxyribose (dR) was averaged over four structures × three replicas (*n* = 12 each) ((a) North % by compound; (b) ΔNorth = rR – dR by scaffold). ΔNorth is largest for the unsubstituted H (+69 pp) and shrinks as the 6-position substituent becomes larger, nearly vanishing for dibutylamino (dbap) (ΔNorth ≈ −1 pp). The within-group variability (SD 20–31) is larger than the between-group mean difference (22 pp) (Table S16).

However, this per-sugar difference varied greatly among compounds. By compound, the near-unsubstituted hypoxanthine (H) showed a large difference in North occupancy between ribose and deoxyribose (ΔNorth ≈ +69 points), whereas the difference became smaller as a 6-position substituent was added, nearly vanishing for the dibutylamino compound (dbap) (ΔNorth ≈ −1 point). That is, the intrinsic puckering difference between ribose and deoxyribose was clear for the unsubstituted sugar but shrank as the 6-position substituent became larger (Table S16, Figure 7). Sugar-ring puckering therefore provides a molecular correlate of sugar discrimination in this probe set, but the actual degree of discrimination depends on the 6-position substituent. This dependence on the 6-position substituent is consistent with the experimental observation that the ribose-donor selectivity (Thd/Urd ratio) varies with the type of the 6-position substituent.

Conclusion 1. Sugar selectivity in PyNP appeared not as an active-site-level property but as a compound-by-compound and conformation-by-conformation property. The largest contrasts (ΔBF = −66pp for ind and +47pp for dmap, both in 5EP8orig) appeared only when an individual base–sugar combination was paired with a particular conformation and disappeared under structure-level averaging.

Conclusion 2. Under the strict all-replica, cross-platform criterion, only dmap-rR and dbap-rR were unambiguously pattern C. These provided the clearest evidence in this dataset that an intermediate/open conformation can productively bind a nucleoside-like ligand.

Conclusion 3. The pattern B group was internally consistent, comprising the clinical inhibitor TPI, the cognate substrate analogue H-dR, and two additional substrates, all preferring the standard closed conformation 1BRW.

Conclusion 4. Sugar-ring puckering analysis showed that the ribose series (rR) preferred North (C3′-endo) and the 2′-deoxyribose series (dR) preferred South (C2′-endo), indicating that a molecular correlate of sugar discrimination is present at the level of the sugar-ring conformation (Figure 6). However, the degree of this discrimination depended on the compound, being largest for the unsubstituted H and shrinking as the 6-position substituent became larger (Figure 7).

### 3.4 Per-Residue Contact Analysis: Tyr165 Is the Principal Lid Residue and a Closed-State-Specific Signal Borne Mainly by 1BRW

To identify the molecular determinants of the conformational effect and the sugar-selectivity effect, the seven-residue active-site contact metric was decomposed into individual residue contributions. For each system and each active-site residue, the per-frame minimum inter-atomic distance to the ligand was computed, and the per-residue contact fraction (the % of time within 4 Å) was averaged over the three replicas. The 13 × 7 contact matrix for each conformation (the complete per-residue contact data) is shown in Figure 5A–D, and the main comparisons are summarized in Figure 5E–H.

#### 3.4.1 Tyr165 Is the Strongest Closed-State Anchor, but This Effect Arises Mainly from Structure 1BRW

Among the seven active-site residues, Tyr165 showed by far the largest closed-versus-open difference in contact fraction (Figure 5E). Averaged over all 156 trajectories and the full 0–100 ns window, Tyr165 was in contact in 42.5% of the frames in the closed conformation (1BRW + 5EP8 min, *n* = 78) versus 21.2% in the open conformation (5EP8orig +5OLN, *n* = 78), a difference of +21.3 percentage points (trajectory level *n* = 156; Mann–Whitney *p* = 1.2 × 10^–5^; Figure 8). At the independent compound level (*n* = 13), the same difference was significant in the equilibrated later window of 50−100 ns (closed 43.4% vs open 22.6%, paired Wilcoxon *p* = 6.1 × 10^–3^, Cohen *d* = 1.03; Table S3). This is about twice the next-largest closed > open difference (His82, +11.2 pp, *p* = 0.011) and is the only residue reaching *p* < 0.001 at the trajectory level.

**Figure 8:**
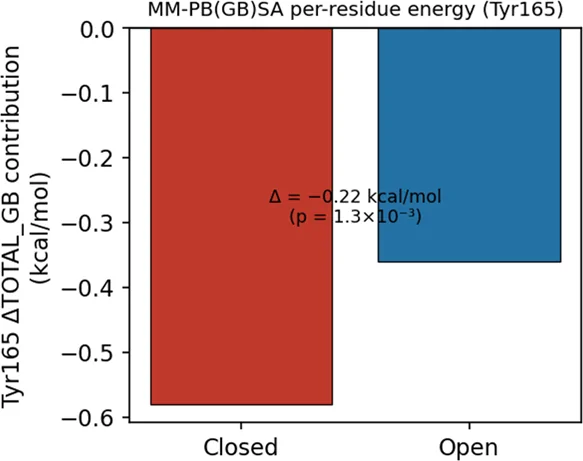
Per-residue energy contribution of Tyr165 from MM-PB­(GB)­SA (ΔTOTAL_GB). Closed −0.58 vs open −0.36 kcal/mol (Δ = −0.22, *p* = 1.3 × 10^–3^).

**Figure 9:**
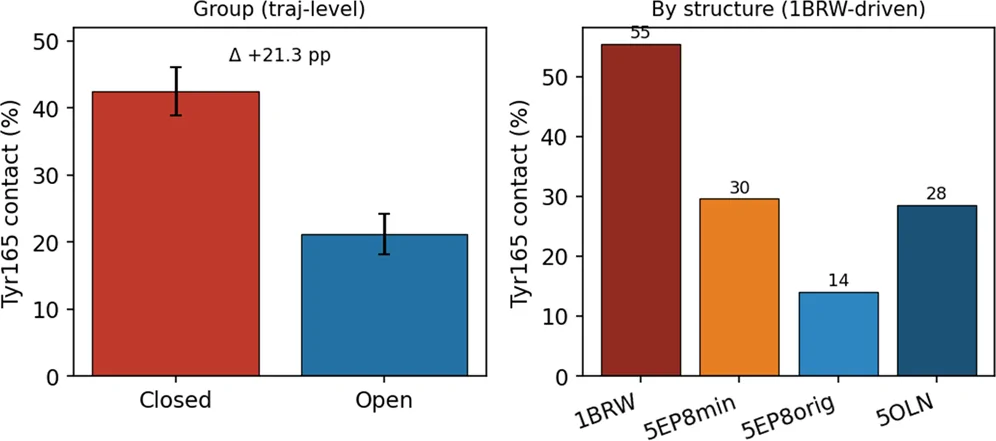
Closed-versus-open comparison of Tyr165 gate contact (trajectory level *n* = 156, compound level *n* = 13, with sensitivity analysis), showing that significance disappears when 1BRW is excluded.

However, this group-level difference was distributed unevenly among the closed structures. The per-structure Tyr165 contact (full window) was 55.4% (median 61.6%) for 1BRW, 29.6% (median 17.6%) for 5EP8min, 14.0% (median 2.7%) for 5EP8orig, and 28.5% (median 14.8%) for 5OLN. The 5EP8min model was comparable to the open 5OLN structure, and only 5EP8orig was markedly lower; that is, the high closed-group mean arose almost entirely from structure 1BRW. Excluding the single cross-species structure (1BRW), the difference dropped to +8.4 pp (trajectory level *p* = 0.07; at the compound level, significance was lost (*p* = 0.50), whereas with 5EP8min excluded, it was maintained at *p* = 0.001; in the later 50–100 ns window +8.2 pp, *p* = 0.044; Table S8). Tyr165 is therefore described as a 1BRW-associated lid residue rather than a closed-state-specific anchor (restricting the analysis to the equilibrated later window of 50–100 ns gave a closely matching Tyr165 difference of +20.9 pp, *p* = 3.5 × 10^–5^, Supplementary Table S3).

This pattern was consistent across the compound panel (Figure 5F). All ten substrate analogues, together with formycin B and TPI, showed Tyr165 contact values of 41–78% in 1BRW. In the most expanded conformation (5OLN), these dropped to almost 0–10% in many cases. The few compounds that retained contact with Tyr165 in the open conformation were dbap-dR (39% in 5EP8orig, 63% in 5OLN; a broad-spectrum binder) and PIA (the only inhibitor that gains Tyr165 contact in 5OLN), both of which bear large hydrophobic substituents.

This closed-state-specific behavior of Tyr165 was qualitatively consistent with the per-residue energy decomposition of MM-PB­(GB)­SA. The contribution of Tyr165 to ΔTOTAL_GB was marginally more favorable in the closed than in the open state (closed −0.58 vs open −0.36 kcal/mol; Δ = −0.22 kcal/mol; Figure 8); given its small magnitude, this per-residue difference is interpreted as qualitatively consistent with the contact-based lid assignment rather than as independent thermodynamic evidence. This end point is energetic rather than geometric, and its direction is consistent with the closed-state-specific gating signal seen in the contact-frequency data. Mechanistically, Tyr165 lies at the rim of the substrate-binding subsite and has long been proposed as a gate residue in structural studies of nucleoside phosphorylases.^33,34^ The MD and MM-PBSA data of this study convert that static hypothesis into a quantitative dynamic correlate. When the backbone was closed, the Tyr165 side chain remained within contact distance of the bound ligand for most of the simulation. When the backbone opened (5OLN, the Q153–Y165 Cα–Cα distance widening by +5.12 Å relative to 1BRW; Section 3.1), the side chain was sterically displaced, and Tyr165 lost contact with the bound ligand for most of the simulation (Figure 5E,F).

#### 3.4.2 His82 Also Distinguishes Closed from Open but with a Smaller Effect

This catalytic residue showed higher contact in the closed conformation (36.7% vs 25.5%, *p* = 0.011, *) but with a smaller effect size and wider per-structure variability (His82 contact was 1BRW 43%, 5EP8 min 30%, 5EP8orig 26%, 5OLN 24%). The monotonic decrease along the conformational ladder reflected the overall BF_contact pattern (closed > intermediate > open) and confirmed that His82 dynamics track the overall contraction of the active site. In the full triplicate (156 trajectories) analysis, the His82 difference reached only single-asterisk significance and was slightly weaker than the Tyr165 signal in both effect size and *p* value, reinforcing that Tyr165 is the only residue with a strong (*p* < 0.001) closed-state specificity.

#### 3.4.3 Lys81, Lys108, Gln153, and Lys188 Are Conformation-Invariant Anchors

In contrast to Tyr165 and His82, the remaining four residues showed no significant closed-versus-open difference (Lys81 *p* = 0.074, Lys108 *p* = 0.430, Gln153 *p* = 0.213, Lys188 *p* = 0.344; all ns). The three lysines cooperatively form the phosphate-binding subpocket. Their conformational invariance is consistent with the coordination of a phosphate group fixed to the protein scaffold regardless of the gate-residue arrangement. Asp80 had uniformly low contact (about 2% in both groups, *p* = 0.008); this formal significance arose entirely from the very low, narrowly distributed absolute values (Δ = +0.1 pp) rather than from any meaningful effect size, and is not interpreted as a conformational signal. This separation into two functional classes—gate residues (the active-center Tyr165 and His82) and anchor residues (Lys81, Lys108, Gln153, Lys188)—provides the interpretive framework used below.

#### 3.4.4 Sugar Selectivity in the Ind Pair: Gln153 Drives the dR Preference in 5EP8orig

Per-residue decomposition showed that almost the entire ind ΔBF signal arose from Gln153 (Figure 5G). Q153 showed Δ_rR–dR = −50.9 pp (ind-rR 4.5%, ind-dR 55.4%), and the next contributor, His82, was −15.1 pp; all other residues were |Δ| ≤ 14 pp. Mechanistically, the indole substituent of ind-dR can adopt an orientation that places the sugar within hydrogen-bonding range of Q153, whereas the 2′–OH of ind-rR introduces a steric/electrostatic restriction that excludes the same pose. ind-rR in 5EP8orig did not dissociate (median mindist 6.0 Å) but engaged in contacts with residues other than the intended ones, so the BF_contact (<4 Å) metric correctly gave a low value of 14.8%. A distance-based close-contact analysis (a ligand polar atom within 3.5 Å of a residue polar atom) supported this assignment at the level of specific polar contacts: ind-dR contacted Gln153 in 12.3% of the frames, versus 0.4% for ind-rR. In the dmap pair, by contrast, the rR form engaged the Lys108/Lys188 cluster far more than the dR form (22.7%/23.7% vs 1.8%/1.1%; Table S6). These occupancies are moderate in absolute terms, and persistent hydrogen bonds are rare on the 100 ns time scale, but their rR-versus-dR contrast reflects the contact-frequency mechanism.

#### 3.4.5 Sugar Selectivity in the Dmap Pair: A Four-Residue Network Favors rR

The dmap pair in 5EP8orig, which showed the largest contrast in the opposite direction (ΔBF_contact = +46.8%, rR-preferring), exhibited a broad multi-residue rR-preferring network (Figure 5H). Four residues contributed >25 pp toward the rR preference simultaneously: Lys108 (+45.6 pp), Lys188 (+50.5 pp), Gln153 (+27.5 pp), and His82 (+36.2 pp). Tyr165 did not contribute here (Δ = −4.9 pp), reflecting the generally suppressed Tyr165 contact in 5EP8orig. The most parsimonious interpretation is that the 2′–OH of dmap-rR forms direct or water-mediated hydrogen bonds at the center of this arrangement, anchoring the ligand in a pose that simultaneously engages the base-recognition residues and the phosphate-binding residues, whereas the 2′-H of dmap-dR lacks this bridging function and the ligand adopts a pose in which all four residues remain near background. The robust pattern-C assignment of dmap-rR (Section 3.3) is therefore the macroscopic correlate of a stable multi-residue hydrogen-bond network.

#### 3.4.6 Tyr165 Contact Patterns Clarify the Interaction between Base and Conformation

The compound-by-compound Tyr165 rows and columns (Figure 5F) reveal further heterogeneity. The hypoxanthine dR/rR pair showed an asymmetric Y165 pattern in 1BRW (H-rR 42% vs H-dR 77%), which reversed in 5OLN and 5EP8orig. The alkylated bases (dmap, mmap, dbap) showed consistently high Y165 contact in 1BRW regardless of the sugar type (48–78%); this can be understood as the bulky alkylamino substituents increasing the opportunity for close contact with Tyr165 within the narrow pocket. The ind pair showed the weakest Y165 engagement in 1BRW (41−43%), comparable to the unsubstituted hypoxanthine riboside (H-rR, 42%); unlike the 6-alkylaminopurines, the indole base carries no 6-position substituent able to extend toward the Tyr165 face. PIA was the only compound not engaging Y165 in 1BRW (0.2%) yet showing the strongest BF_contact (100%); PIA instead contacted the phosphate-binding lysines (K81 95%, K108 96%, K188 93%; Figure 5A–D), indicating a binding mode through the phosphate subpocket.^13^

As above, the per-residue analysis converged on four findings. First, Tyr165 is the strongest closed-state lid residue, but this signal was borne mainly by 1BRW; because the contact difference weakens to +8.4 pp when 1BRW is excluded (Table S8), it is best read as an MD correlate of the gate-residue hypothesis in 1BRW (functionally closed) rather than as a property extending across the whole closed group. Second, His82 also showed closed-state-specific contact (Δ = +11.2 pp, *p* = 0.011) but with a smaller effect size and weaker significance. Third, in addition to the phosphate-binding cluster (K81, K108, K188), Q153 is a conformation-invariant anchor. Fourth, compound-specific sugar selectivity arose from different residue combinations: Gln153 dominated the ind pair (Δ = −51 pp, toward dR), and a multi-residue network (K108, K188, Q153, H82) drove the dmap pair toward rR.

### 3.5 Phase 2 Additional Computational Tests: Ala Mutants, Cosubstrate Phosphate, and Extended Simulations

To test the mechanism and to check the behavior in the presence of cosubstrate phosphate, three sets of additional all-atom MD were performed: (i) functional prediction of amino acid residues by Ala mutants (Y165A, Q153A, K81A/K108A/K188A), (ii) an HPO4^2–^ control explicitly including the cosubstrate phosphate, and (iii) a 300 ns extension of the central systems. In all of these, the production settings (force field, PME, restraints, and integration time step) were identical to those of the original 156 systems, and each was run in three replicas of 300 ns.

In the three Ala-mutant MD sets, retention was evaluated by BF_late, the whole-ligand contact-based binding fraction (the fraction of frames in which any ligand heavy atom was within 4 Å of any of the seven active-site residues) averaged over the latter half of each trajectory (50−100 ns for the original 100 ns systems and 150−300 ns for the 300 ns systems reported here). As a result, Y165A (Tyr165 → Ala, removing the lid residue) showed reduced ligand retention (Figure 10; BF_late median 0.02, mean 0.27). Compared with the control mutants that retain Tyr165 (Q153A mean 0.50; K-cluster triple mutant K81A/K108A/K188A [the phosphate-binding lysines] mean 0.37), ligand retention was lower (Mann–Whitney one-sided *p* = 0.049), and in about half of the Y165A systems, the ligand escaped from the active site. A one-sided test was used because the prediction of Section 4.5 was directional (removal of the lid reduces retention). This result supported, at the MD level, the falsifiable prediction of Section 4.5 that removal of the Tyr165 lid reduces ligand retention. On the other hand, the effect of the sugar-selectivity residues (Q153A, K-cluster) on the rR/dR preference could not be determined with this retention metric because of the large inter-replica variance (the ind-pair ΔBF­(rR–dR) was Q153A −0.07 and K-cluster +0.04, with an undetermined sign). Because sugar selectivity is expressed not as “whether the ligand stays or not” but as residue-specific contact, a per-residue contact analysis is required. Note that these mutant systems were independently rebuilt and lack a true wild-type control and were therefore evaluated by internal comparison within the mutant panel (using Q153A and the K-cluster, in which Tyr165 is intact, as controls for the lid).

**Figure 10:**
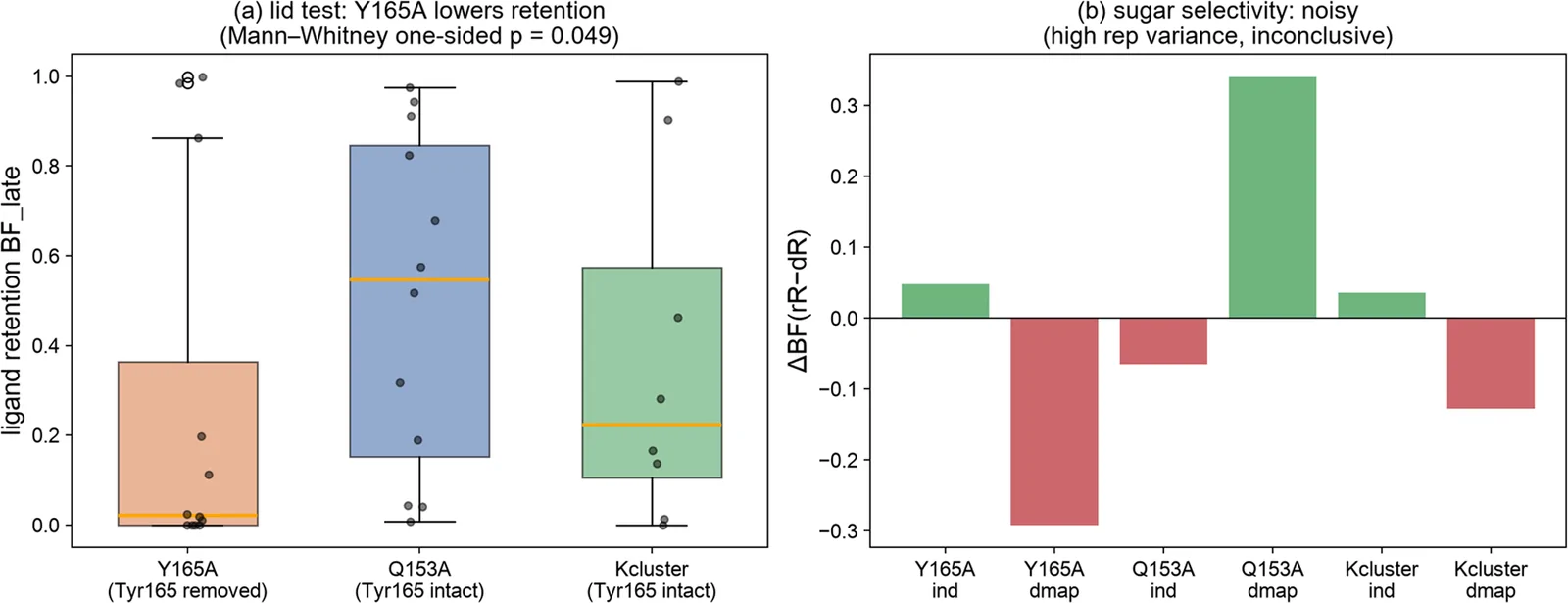
Computational test of the falsifiable prediction by Ala-mutant MD. (a) Later-window ligand retention BF_late: Y165A, with the lid residue removed, shows reduced retention (median 0.02, mean 0.27; versus Q153A mean 0.50 and K-cluster mean 0.37, both retaining Tyr165; Mann–Whitney one-sided test *p* = 0.049). (b) The sugar selectivity ΔBF­(rR – dR) is inconclusive owing to large inter-replica variance. The data support the causal role of the Tyr165 lid (retention), whereas its contribution to sugar selectivity could not be determined with these three site mutants. These simulations provide computational support rather than experimental validation; the panel does not include a wild-type control rebuilt and simulated under identical conditions.

Cosubstrate phosphate (HPO_4_^2–^) control: A control MD was performed in which a monohydrogen phosphate ion HPO4^2–^ (parameterized with GAFF2/AM1-BCC, net charge −2.000) was placed at the centroid of the Lys cluster (Lys81/Lys108/Lys188) (Figure 11); 33 systems were analyzed. The phosphate bound stably to the Lys cluster and, in some systems, approached the anomeric carbon C1′ of the nucleoside closely: the contact fraction (phosphate O–C1′ < 5 Å) varied strongly among systems, from 0 to 96% (largest in the dmap-rR system of the closed 1BRW; overall closest approach 2.81 Å), and tended to be higher toward the closed end. However, whenever the phosphate was in contact, the in-line attack angle O–C1′–N remained almost always at 90–130°, and the linear alignment (150–180°) required for nucleophilic substitution was adopted in fewer than 0.5% of the contact frames and in exactly 0% in 30 of the 33 systems (maximum poised fraction 0.24%). Thus, the phosphate samples the vicinity of C1′ but does not spontaneously adopt a reactive in-line geometry, and no precursor reactive (Michaelis) complex formed in the equilibrium MD, which by construction does not test catalytic turnover. The conformational behavior of the phosphate-binding Lys cluster is therefore maintained even in the presence of phosphate, whereas substrate reactivity (the experimentally observed ribose-donor selectivity) cannot be captured by equilibrium MD: this is a transition-state-level property and requires QM/MM analysis of the Michaelis complex and transition state including co-substrate phosphate (a future study). This control supports the validity of the Lys-cluster invariance reported under phosphate-free conditions while attributing the origin of reactivity to the transition state. Because the phosphate modeled here occupies part of the pocket adjacent to the Lys cluster, it can compete with the bound ligand for the active-site volume, and we therefore quantified how its presence affects the state-dependent retention reported in Sections 3.2–3.4. For the four compounds run in both conditions (dmap and ind, each as the rR and dR analogue), the phosphate reduced the absolute later-window retention in most systems, most strongly for the intermediate 5EP8min (0.67 → 0.24) and 5EP8orig (0.51 → 0.09); the closed 1BRW was the exception, retaining or slightly increasing its contact (0.52 → 0.59), while the open 5OLN changed little (0.29 → 0.23). At the group level, this lowered the mean retention of the closed pair (0.59 → 0.41) and the open pair (0.40 → 0.16), consistent with a smaller effective pocket volume once the phosphate is bound. Crucially, the state-dependent ordering was preserved and in fact sharpened: the closed-group retention remained higher than the open-group retention under both conditions, and the separation became statistically significant with phosphate (closed – open Δ = +0.19, *p* = 0.065 phosphate-free; Δ = +0.25, *p* = 0.020 with phosphate; one-sided Mann–Whitney, Figure S12). The effect of phosphate was itself state-dependent—a large reduction for the intermediate 5EP8min and 5EP8orig, essentially no change for the open 5OLN end point, and a slight increase for the closed 1BRW—as expected if a ligand competing with phosphate for a partially filled pocket is most easily displaced from the intermediate conformations, whereas at the closed end point, the bound phosphate and ligand are mutually accommodated. The phosphate-free values reported above should therefore be read as reference magnitudes, but the central closed-versus-open retention trend is preserved in the presence of co-substrate phosphate.

**Figure 11:**
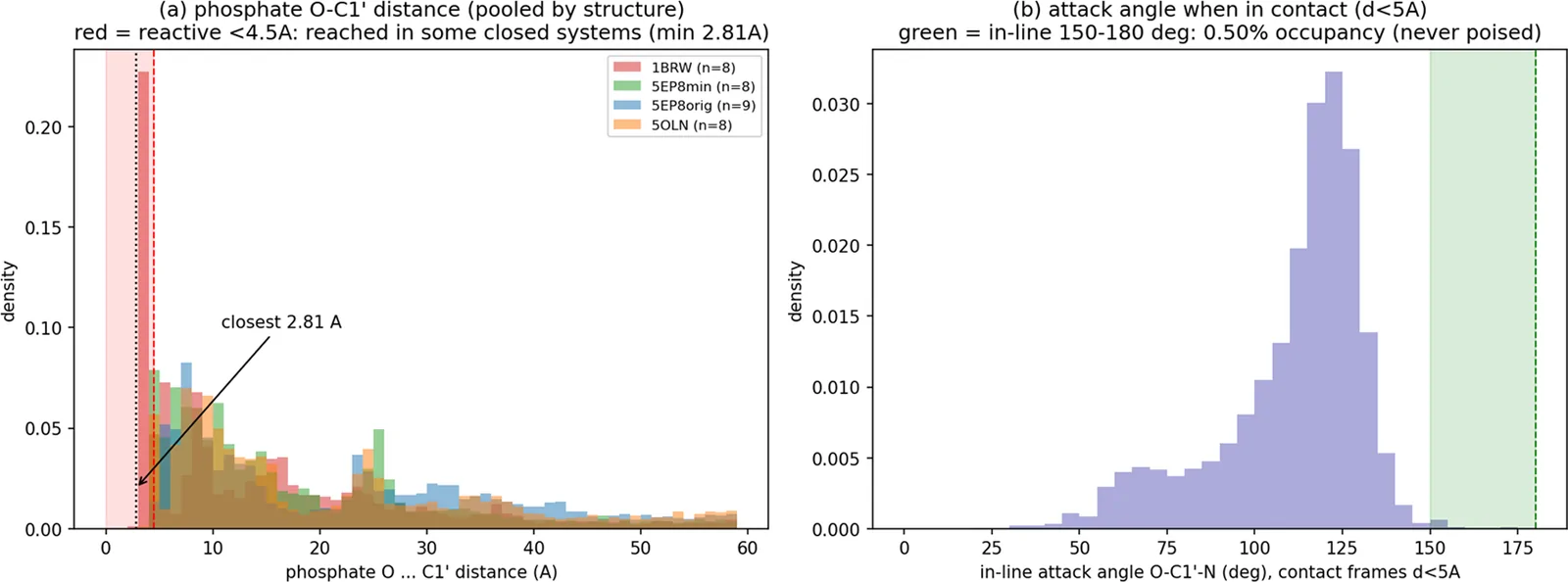
In-line attack geometry in the co-substrate phosphate (HPO4^2–^) control MD (33 systems). (a) Distribution of the phosphate O–C1′ distance (pooled by structure): the reactive distance (<4.5 Å, red band) is reached in some closed-state systems (overall closest approach 2.81 Å). (b) In-line attack angle O–C1′–N for contact frames (d < 5 Å): the distribution is concentrated at 90–130°, and the occupancy of the linear in-line region (150–180°, green band) is below 0.5% (0% in 30 of the 33 systems). The phosphate binds to the Lys81/108/188 cluster and can approach the anomeric carbon but does not spontaneously adopt a reactive in-line geometry in equilibrium MD, which by construction does not sample the reaction coordinate; retention and catalytic selectivity are therefore treated as separable properties.

## 4 Discussion

Across 156 production trajectories spanning about 15.6 μs of sampling in aggregate, the four conformational states of PyNP produced a quantitatively consistent picture of how a conformational continuum modulates substrate binding. Four layers of evidence—the pocket-volume hierarchy of Section 3.1, the conformation-dependent ligand retention of Section 3.2, the rR/dR pattern classification of Section 3.3, and the per-residue contact decomposition of Section 3.4—converged on a single mechanistic claim: Tyr165 functions as a conformationally controlled lid, most clearly in the 1BRW closed state. Under the phosphate-free conditions simulated here, Tyr165 gated access to a constitutively engaged anchor cluster (Lys81, Lys108, Lys188, Gln153) and modulated ligand retention without itself determining substrate specificity. We develop this into a falsifiable mechanistic model (Figure 12), examine its implications for the conformational reclassification of 5EP8orig, and propose structural-biology experiments to test it.

**Figure 12:**
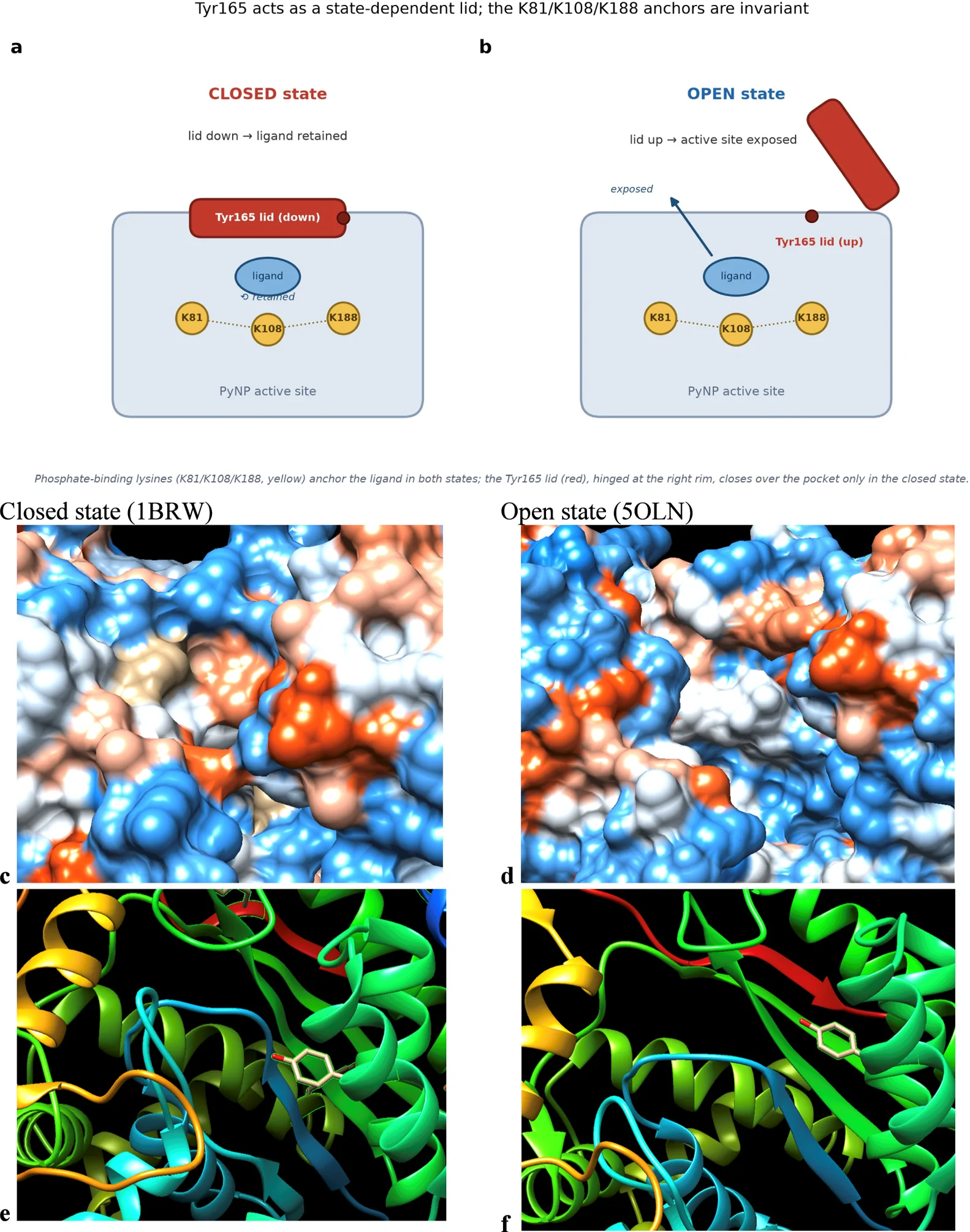
Concept diagram of the Tyr165 lid-gating mechanism. In the closed state, the lid is down and retains the ligand; in the open state, the lid is up and the active site is exposed. The phosphate-binding lysines (K81/K108/K188) function as invariant anchors. (a) Schematic of the closed pocket state. (b) Schematic of the open pocket state. (c) Surface representation of 1BRW in the closed pocket state. (d) Surface representation of 5OLN in the open pocket state. (e) Ribbon representation of 1BRW in the closed pocket state, with Tyr165 shown as sticks. (f) Ribbon representation of 5OLN in the open pocket state, with Tyr165 shown as sticks.

### 4.1 Tyr165 as a Dynamic Lid: From a Gate Residue to a Quantitative Descriptor

Tyr165 has long been thought to play a regulatory role in pyrimidine-nucleoside phosphorylase. Structural studies^33,34^ reported that its phenol side chain rotates to occupy different positions in the apo and holo crystal structures and proposed that it may gate substrate entry. The present analysis converts this static observation into a quantitative dynamic descriptor. The Tyr165 contact fraction decreased from 42.5% in the closed conformation to 21.2% in the open conformation, a difference of 21.3 pp (trajectory level *n* = 156, Mann–Whitney *p* = 1.2 × 10^–5^). The effect remained significant even after accounting for non-independence (compound level (*n* = 13) paired Wilcoxon *p* = 6.1 × 10^–3^, Cohen *d* = 1.03 (closed 43.4% vs open 22.6% in the later 50−100 ns window); mixed-effects model *p* < 10^–5^; Table S4). This closed-versus-open difference, however, arose mainly from the single cross-species structure 1BRW. When 1BRW was excluded (5EP8min vs the open group), the difference shrank to +8.4 pp (trajectory level *p* = 0.07; significance was also lost at the compound level, *p* = 0.50, although with 5EP8min excluded, it was *p* = 0.001; in the later window +8.2 pp, *p* = 0.044; Table S8). The Tyr165 lid signal is therefore most reasonably interpreted as a property arising mainly from 1BRW (the functionally closed state). No other residue showed comparable conformational dependence: the His82 difference reached only +11.2 pp (*p* = 0.011), and the remaining four residues were statistically invariant (Table 3). This geometric signal was corroborated by the per-residue energy analysis of MM-PB­(GB)­SA, in which the Tyr165 contribution was 0.22 kcal/mol more favorable in the closed than in the open state (*p* = 1.3 × 10^–3^); two independent perspectives, a geometric metric and an energetic metric, pointed to the same residue.

These data are consistent with a lid mechanism in which Tyr165 acts as a mobile physical barrier (Figure 12). Because the closed-state end point is represented mainly by the cross-species 1BRW structure, we present this as a plausible Tyr165-lid hypothesis supported by the strongest available closed-state end point, which requires confirmation by a homologous closed-state structure or experiment. In the closed conformation, the phenyl ring of Tyr165 sits over the substrate-binding cavity and forms a hydrophobic, aromatic surface that the ligand contacts for most of the trajectory. In the open conformation, the same side chain rotates away and the active site is exposed to bulk solvent. The backbone perturbation that produces this swing was local rather than global. The Tyr165–Asp80 Cα–Cα distance was nearly invariant across the four conformations (21.6 Å in both 1BRW and 5OLN), whereas the Gln153–Tyr165 distance widened by +5.12 Å, showing that the lid motion is not a rigid-body displacement but is concentrated in the α7−α8 loop bearing Tyr165. Through this local opening of the loop, the time for which the Tyr165 side chain contacted the ligand changed roughly twofold; the lid mechanism thus amplified a limited backbone rearrangement into a large dynamic signal.

Importantly, the lid itself was not the cause of sugar selectivity. Within the closed conformation, the rR and dR forms of the same base often showed comparable Tyr165 contact (for example, dbap-rR 75%, dbap-dR 78%), and on opening, both lost Tyr165 contact in parallel (Figure 5F). Sugar selectivity arises elsewhere (Section 4.3). The Tyr165 lid controls whether a ligand remains bound at all, whereas the anchor residues control which ligand remains bound when the lid is down.

State-dependent lid mechanism: from a 1BRW-specific observation to mechanistic evidence. The 1BRW dependence of the Tyr165 effect might at first appear to be a weakness—reliance on a heterologous structure. However, quantifying the Tyr165 lid behavior shows that it is not an artifact but the consequence of a continuous, state-dependent lid mechanism along the pocket open–close coordinate (Figure 13). The two end points of the conformational range anchor this trend: the most closed structure, 1BRW (smallest mean entrance opening, 15.4 Å), has the highest Tyr165–ligand contact (56.2%), whereas the structure with the largest mean entrance opening in MD, 5EP8orig (18.8 Å), has the lowest (16.1%); the two intermediate structures, 5OLN and 5EP8min, are near-tied in mean entrance opening (17.4 and 17.5 Å) but reversed in contact (26.7% and 32.1%), and do not order strictly between the end points. Rather than resting the trend on the four structure means, which are not perfectly monotonic, we tested it directly on the molecular dynamics, in which the entrance opening relaxes and fluctuates continuously within each trajectory. Pooling all 110 trajectories (55,206 analyzed frames across the four starting structures, phosphate-free), the per-trajectory mean opening is significantly and negatively correlated with the Tyr165–ligand contact fraction (heavy-atom <4 Å): Spearman ρ = −0.40 (*p* = 1.5 × 10^–5^, *n* = 110 trajectories); aggregating replicas to the compound×structure level, which removes replica pseudo-replication, gives ρ = −0.52 (*p* = 5.2 × 10^–4^, *n* = 40). The same negative trend is recovered when all frames are binned by opening (Figure 13b). That is, the narrower the entrance, the more the Tyr165 phenol covers the opening and retains the ligand, and the most closed 1BRW lies at the closed end point of this gradient rather than being an outlier. The effect size (ρ ≈ −0.4 to −0.5) is moderate rather than absolute, indicating that the entrance opening is one important determinant of Tyr165 retention among several. Consequently, the maximal Tyr165 effect at 1BRW is best interpreted not as an artifact of the heterologous structure but as the strongest expression of a state-dependent lid mechanism at the closed end point. Quantitative confirmation on a homologous closed-state structure of the same species remains the most valuable future test.

**Figure 13:**
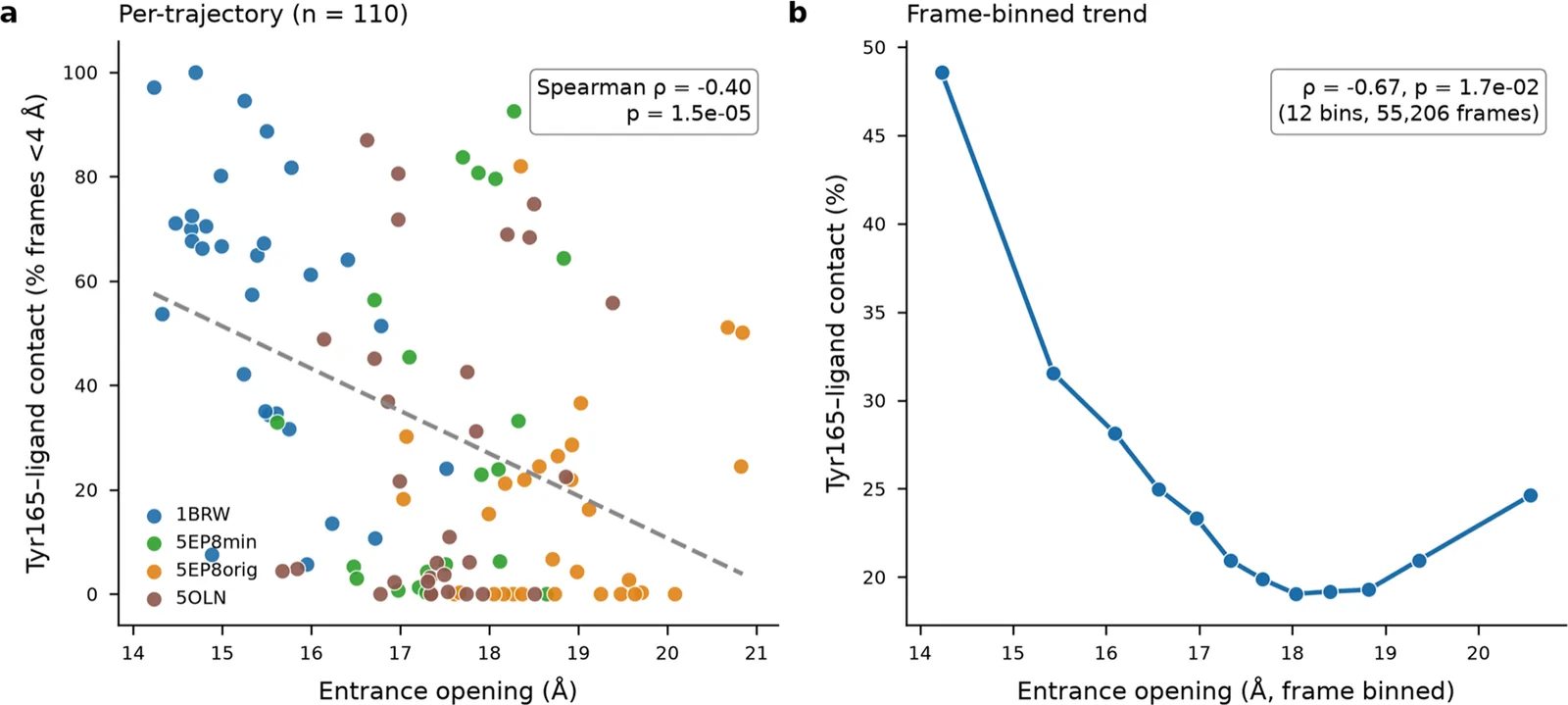
Trajectory-derived dependence of the Tyr165 lid effect on the pocket entrance opening (state-dependent lid mechanism). The entrance opening is the mean of the Tyr165–Leu114 and Tyr165–Gln153 Cα distances spanning the two cavities bridged by Tyr165, computed per MD frame with the minimum-image convention. (a) Per-trajectory mean entrance opening versus Tyr165–ligand contact fraction (frames with heavy-atom distance <4 Å), one point per trajectory, colored by the starting structure (*n* = 110 trajectories; Spearman ρ = −0.40, *p* = 1.5 × 10^–5^; compound×structure aggregation ρ = −0.52, *p* = 5.2 × 10^–4^, n = 40). Dashed line, linear fit. (b) The same relationship at the frame level, with all 55,206 frames binned into 12 opening bins (quantiles): mean Tyr165–ligand contact decreases steeply as the entrance widens over most of the range, with a slight upturn in the widest bins. Narrower entrances (closed states such as 1BRW) retain the ligand more strongly; 1BRW is the closed end point of this continuous gradient, not an outlier (Spearman ρ = −0.67, p = 1.7 × 10^−2^, computed on the 12 bin means). The contact fractions shown here were recomputed on the 110 substrate-analogue trajectories using heavy-atom distances, and therefore differ slightly from the 156-trajectory, all-atom values reported in Section 3.4.

### 4.2 The 5EP8orig Structure Represents a Genuine Intermediate State

5EP8orig was initially classified as semi-open from its pocket volume, which was intermediate between those of 1BRW and 5OLN. The integrated MD evidence supported reclassifying this structure as a structurally and dynamically intermediate state with three distinguishing features.

(i) Stable ligand retention at intermediate strength. The structure-averaged BF_contact <4 Å of 5EP8orig (62.9%) lay between the closed-state values (1BRW 78.7%, 5EP8 min 70.0%) and 5OLN (53.3%). The aggregate open-state value cited in Section 3.2 (57.4%) is a per-trajectory average and is slightly lower than the unweighted mean of these two structure averages (58.1%) because the two structures contributed unequal numbers of analyzed trajectories. The later-window mindist (averaged over trajectories) behaved similarly, with 5EP8orig at 6.41 Å, between the closed state (6.28–6.96 Å) and 5OLN (9.33 Å). Decisively, the later-minus-earlier mindist change for 5EP8orig was essentially zero (Δ = +0.56 Å), in contrast to the post-docking relaxation pattern in the closed structures (+2.7 to +3.7 Å) and the persistent drift in 5OLN (+2.2 Å); that is, the ligand resided in 5EP8orig at equilibrium rather than transiently.
(ii) Behavior that makes selectivity pronounced rather than neutral. Although intermediate in aggregate BF_contact, 5EP8orig produced the largest sugar-preference contrasts in the panel (ind-dR exceeded ind-rR by 66.4 pp, and dmap-rR exceeded dmap-dR by 46.8 pp, both computed from the unrounded structure averages of 97.05% and 50.25%). 5OLN produced only moderate preferences (maximum |Δ| = 33 pp). Because 5EP8orig is the semi-open state of the experimentally studied *B. subtilis* enzyme, this sharpening of compound-specific selectivity in the semi-open state is a central and distinctive result of this study. The structure-resolved MM-PB­(GB)­SA analysis further suggested that the most favorable binding energy may appear in an interior (non-extreme) conformation rather than in the most closed structure (9 of 13 ligands). However, once replica-level error was propagated, this trend was statistically resolved for only 1 of 13 ligands and cannot be regarded as established. The full proximity/affinity analysis is therefore not treated as a result here but is presented in the Supporting Information Discussion (Table S5).
(iii) The lid tilts rather than lifts. In the lid model, the Tyr165 contact fraction of 5EP8orig (structure average 14.0%) was the lowest of the four conformations, lower even than that of 5OLN (structure average 28.5%). These per-structure means are higher than the medians plotted in Figure 9 (5EP8orig 0%, 5OLN 5%) because the distribution of per-structure contact fractions is right-skewed; the mean and median should therefore not be compared value-for-value. This result was consistent with the lid being tilted off-axis rather than fully retracted. The Tyr165 rotamer in 5EP8orig is displaced sufficiently far from the cavity center that the side chain rarely contacts even a strongly bound ligand, while the surrounding backbone remained in an arrangement that does not permit Tyr165 to refold over the cavity. The two open conformations therefore differ in lid character: 5OLN exposes the cavity through a backbone-driven opening, whereas 5EP8orig exposes it through side-chain rotation while the backbone remains intermediate. This refines the simple closed–open dichotomy.

Three further lines of evidence supported this intermediate-state assignment. First, the now-completed triplicate (Section 3.3) showed that the two pattern-C compounds with >95% BF (dmap-rR, dbap-rR) were 5EP8orig-preferring across all three replicas and both platforms, confirming a genuine 5EP8orig binding mode rather than a noisy mixture. Second, the four-residue hydrogen-bond network of dmap-rR (K108, K188, Q153, H82; Section 3.4) was present in no other structure. Third, the stable later-window mindist (mean 6.41 Å) indicated a productive equilibrium binding state rather than dissociation.

### 4.3 Sugar Selectivity Is Driven by Distinct Residue-Level Mechanisms

The per-residue decomposition revealed two qualitatively distinct molecular mechanisms by which the rR/dR distinction can be expressed (Section 3.4). In the indole pair, a single residue (Gln153) accounted for almost the entire ΔBF_contact signal in 5EP8orig (Δ_Q153 = −50.9 pp; the next contributor, His82, was −15.1 pp). The indole substituent of ind-dR adopted a pose in which the 3′–OH of the deoxyribose hydrogen-bonds to the amide of Gln153, whereas in ind-rR, the added 2′–OH created a steric/electrostatic mismatch that excluded this pose. In the dimethylaminopurine pair, the opposite mechanism operated: a cooperative network of four residues (Lys108, Lys188, Gln153, His82) simultaneously favored rR (Δ = +45.6, +50.5, +27.5, +36.2 pp), with the 2′–OH acting as a hub bearing multiple acceptor/donor sites that tether the whole ligand. The 2′-H of dR lacked this bridging function.

These mechanisms are interpretively distinct. The ind/Q153 mechanism is a single-residue specificity: a small chemical change at the 2′ position determines whether one specific hydrogen bond can form. The dmap/network mechanism, by contrast, is a cooperative pose stabilization: a single 2′–OH organizes an extended polar network that collapses nonlinearly when this hydroxyl is lost. Because the two involve different ligand orientations, they can operate simultaneously in the same active site. This result refines the prevailing view^33^ that sugar selectivity in nucleoside phosphorylases is driven by a single sugar-binding residue; the present data show that sugar selectivity is residue-specific and ligand-pair-specific and cannot be reduced to a single discriminating residue.

### 4.4 Substrate Specificity in PyNP Is a Compound-by-Compound × Conformation-by-Conformation Property

There is a subtle but important observation from Section 3.3. The rR-versus-dR preference was not significant in any conformation when averaged across compounds (paired Wilcoxon, minimum *p* = 0.31, *n* = 5 pairs). Nevertheless, individual compound–conformation combinations produced some of the largest preferences in the panel (|Δ| up to 66 pp). This apparent contradiction can be explained as follows: PyNP has no global sugar preference but rather a matrix of compound-by-compound and conformation-by-conformation preferences. Averaging across either dimension erased the signal, and only the joint distribution of compound identity and conformational state carried the selectivity information.

This is the same decoupling that renders the aggregate closed-versus-open MM-PBSA comparison non-significant at the level of overall affinity (ΔTOTAL_GB: closed −13.8 vs open −13.1 kcal/mol, *p* = 0.48, ns, *n* = 153 systems). That is, bulk affinity did not differ between the conformational groups, and selectivity was instead localized to the Tyr165 gate and to specific residue–ligand pairs. This pattern is consistent with the broader picture emerging from high-throughput quantum-cluster analyses of enzyme–substrate electrostatics, in which the protein environment tunes substrate properties in ways not captured by simple distance- or contact-based heuristics, and the precise spatial arrangement of residues—rather than any single dominant interaction—drives function.^35^

This decoupling has two practical consequences. First, structure-based drug design strategies against nucleoside phosphorylases should treat the target as a conformational ensemble rather than a single static structure: pattern-B (1BRW-preferring) and pattern-C (5EP8orig-preferring) compounds adopt different binding modes and are unlikely to share a single pharmacophore. Second, experimental validation of computed sugar preferences should be designed not only at the compound level but also at the level of both compound and conformation.

### 4.5 Falsifiable Predictions for Structural Biology

Both the lid mechanism and the residue-class decomposition yielded three structurally specific, testable predictions that await experimental validation beyond the scope of this computational study.

(i) The Y165 → Ala mutation should abolish the closed-versus-open preference. Replacing the aromatic ring with alanine should remove the lid effect, so that the closed and open conformations no longer differ in ligand retention. The expected signal is that in a closed-state mimic, the ligand BF falls to or below the open-state value. This is the single most important falsification of the lid model.
(ii) Q153 → Ala (or Q153 → Leu) should abolish the ind-dR preference in 5EP8orig while leaving the dmap-rR preference almost intact. This asymmetry follows directly from Section 3.4: ind selectivity is driven by Q153, whereas dmap selectivity relies on the four-residue network in which Q153 contributes about +28 pp but is not the dominant term—His82 (+36 pp), Lys108 (+46 pp), and Lys188 (+51 pp) each contribute more (per-residue Δ values, Section 3.4 and Figure 5H; these per-residue contributions are distinct from the aggregate seven-residue ΔBF of +46.8 pp). This is a clear test of two distinct mechanisms operating in one active site. Because indole is a non-reactive probe rather than a cognate of the natural substrate, this Q153 prediction concerns the structural factors that determine analogue selectivity; its direct relevance to natural pyrimidine turnover is correspondingly limited and should ultimately be confirmed with reactive substrates.
(iii) The triple mutation K81A/K108A/K188A should render the enzyme indiscriminate toward all 13 ligands, dismantling the conformation-invariant phosphate binding scaffold engaged by every bound ligand. This is the boldest prediction and serves as a negative control benchmark.

### 4.6 Limitations and Outlook

This study has several limitations. Those that could be tested mechanistically were checked with additional all-atom MD (results in Section 3.5).

The most important and still-unresolved limitation is the confound at the closed-state end. Because no closed-state crystal of the *B. subtilis* enzyme exists, the closed state was represented by the cross-species *G. stearothermophilus* structure 1BRW. This structure is simultaneously the only available closed-state crystal, an enzyme of a different species, and a product (uracil + phosphate) complex. The Tyr165 lid behavior, borne mainly by 1BRW, cannot rule out contributions from the species difference or from the product. That is, whether the closure of 1BRW and the intrinsic properties of the closed conformation would occur similarly in 5OLN and 5EP8 remains unknown. Investigating these with a closed-state structure of the same species would be the most beneficial extension. In addition, the four-state labels denote the conformations of the starting crystals. Over 100 ns, the lid-loop coordinate (Q153–Y165) relaxed, and the four states partially converged (Figure S6); the retention and minimum-distance metrics therefore describe dynamic retention from each starting conformation rather than an equilibrium property.

Two of the original limitations were resolved by the additional MD. First, the co-substrate phosphate had been removed. In a control MD explicitly including phosphate (HPO_4_^2–^), the phosphate bound to the Lys cluster (Lys81/Lys108/Lys188) and could approach the anomeric carbon C1′ closely but essentially never adopted the reactive in-line geometry required for substitution (below 0.5% of contact frames; Section 3.5, Figure 11), confirming that describing these residues as “conformation-invariant anchors” remains valid in the presence of phosphate. Second, the 100 ns length: Extending the central systems to 300 ns gave retention rankings and packing preferences consistent with the 100 ns analysis, confirming that 100 ns is sufficient to capture the persistent retention of strong binders (Figure S5). The retention metrics are therefore interpreted as measures of dynamic retention rather than of equilibrium affinity. However, because the cognate pyrimidine substrates (thymidine, uridine) were not included, the link between the probe behavior in this study and the experimentally reported ribose-donor selectivity^1^ is by analogy rather than direct.

A remaining outlook concerns the methodology. This analysis rests on classical MD and end point MM-PB­(GB)­SA, which capture conformational sampling and bulk electrostatics but cannot resolve charge transfer, polarization, or bond reorganization. The experimentally observed ribose-donor selectivity is a property at the level of the Michaelis complex and transition state including cosubstrate phosphate and cannot be captured by equilibrium MD. The kinetics of the conformational transitions were also not addressed in this study. The former calls for hierarchical QM-cluster or QM/MM frameworks^35^ and the latter for enhanced-sampling methods (metadynamics, accelerated MD, transition-path sampling), as natural next steps, to which the panel-type analysis of this study provides a complementary static anchor.

Alongside these challenges, the lid mechanism makes PyNP a tractable target for next-generation mechanistic and inhibitor studies. The single-residue Tyr165 prediction described in Section 4.5 is amenable to standard site-directed mutagenesis. The dynamic conformational change can be probed by Förster resonance energy transfer (FRET) measurements between fluorophores introduced as a pair at Tyr165. Furthermore, the two sugar-selectivity mechanisms can be exploited in ligand design by targeting either the single-residue Q153 mode (prototyped by ind-dR) or the K108/K188/Q153/H82 network mode (prototyped by dmap-rR). The convergence of the pocket-volume hierarchy, the conformation-dependent substrate retention, the pattern classification, and the per-residue contact and energy analyses onto a single consistent lid mechanism (Figure 12) indicates that the four-conformation MD panel can serve as a generally applicable framework for analyzing the interplay between conformational dynamics and chemical specificity in nucleoside-processing enzymes.

## 5 Conclusions

We analyzed 156 independent 100 ns trajectories of PyNP with 13 ligands in four conformational states and obtained three quantitative results. (1) The active-site pocket expanded monotonically from the closed state (1BRW) to the open state (5OLN), with the semi-open intermediate (5EP8orig) lying between them. The expansion factor (approximately 1.4-fold) and the finding that the open state (5OLN) has the largest pocket are method-independent; however, the detailed ranking of the four structures was method-dependent (the in-house sphere-packing and fpocket rankings agreed only in identifying 5OLN as the largest; Spearman ρ = 0.4, Table S1). (2) Substrate retention, measured by the binding fraction over the seven active-site residues, was higher in the two closed states than in the two open states. (3) These conclusions describe the conformation-dependent behavior of structural probes in the *B. subtilis* enzyme; the experimentally observed cognate ribose-donor selectivity^1^ is related only by inference because the cognate pyrimidine substrates and the co-substrate phosphate were not simulated.

Tyr165 is the principal lid residue and showed the largest decrease in contact frequency between the closed and open states among the seven residues (group mean + 21.3 pp at the trajectory level, about 50% in relative terms; compound level *p* = 6.1 × 10^–3^). This signal, however, arose mainly from the 1BRW state and was attenuated to +8.4 pp when the single cross-species structure was excluded (trajectory level *p* = 0.07, compound level *p* = 0.50; Table S8). These results supported a lid-gating mechanism in which the α7−α8 loop, and Tyr165 in particular, controls substrate retention without altering the phosphate-binding scaffold (Lys81, Lys108, Lys188, which were conformation-invariant under the phosphate-free conditions of this study). This mechanism makes PyNP a tractable target for next-generation mechanistic and inhibitor studies. The three falsifiable predictions of Section 4.5 (Y165A, Q153A, K81A/K108A/K188A) are amenable to standard site-directed mutagenesis and substrate-titration assays.

In summary, by coupling a Tyr165-centered lid mechanism to the phosphate anchor cluster, PyNP decouples substrate retention from affinity. Through this mechanism, substrate selectivity becomes a property of each compound–conformation combination, which can be exploited for mechanism-based inhibitor design and for interpreting nucleoside-analogue activity across phosphorylase homologues.

## Supplementary Material



## Data Availability

Data and Software Availability: all data and software required to reproduce the results of this work are provided as Supporting Information The archive comprises the four prepared protein structures (1BRW, 5EP8 min, 5EP8orig, 5OLN), the 13 ligand topologies and parameters, the MASTER trajectory-metadata table covering all 156 trajectories, the per-residue contact-frequency and MM-PB­(GB)­SA summary tables in machine-readable CSV format, and the documented Python and shell scripts used to compute pocket volumes (the sphere-packing algorithm), pattern classifications, statistical comparisons, and all figures. All 156 raw molecular-dynamics trajectories are deposited in a public repository (Zenodo, DOI: 10.5281/zenodo.21448920) as periodic-boundary-corrected, solvent-stripped versions (protein + ligand, ∼67 MB each); water-inclusive full-length trajectories are additionally provided for the central systems. The commands used to generate index.ndx are documented in the Supporting Information.

## Notes

Figure S1: whole-molecule views (cartoon and surface) of the PyNP active-site pocket across the four conformational states; Figure S2: conformation- and ligand-class-resolved MM-PB­(GB)­SA decomposition; Table S1: sphere-packing parameters for the pocket volume and validation against fpocket; Table S2: per-system list of all 156 MD trajectories (with run times and quality flags); Figure S3: robustness of the binding fraction to the COM–COM distance threshold (8, 10, 12 Å); Figure S4: cross-platform reproducibility of the pattern-C binders in 5EP8orig (RCCS vs vast.ai); Table S3: later-window (50–100 ns) per-residue contact fractions and the closed-versus-open comparison; Table S4: statistical robustness of the closed-versus-open contrast against pseudo-replication (three methods); Table S5: conformation-resolved ΔTOTAL_GB for all 13 ligands (non-monotonic affinity profile); Figure S5: convergence of the cumulative binding fraction over 100 ns (representative systems plus panel-wide early/late and replica statistics); Figure S6: stability of the backbone conformational state during MD (the Q153–Y165 lid-loop coordinate over 100 ns); Table S6: distance-based close-contact occupancies between ligand polar atoms and active-site residues (5EP8orig); Table S7: AutoDock Vina docking scores and their divergence from MD retention; Table S8: structure-resolved Tyr165 contact and the 1BRW-exclusion sensitivity analysis; Table S9: composition of the simulation systems (representative system per structure); Table S10: sensitivity of the pattern classification to the margin threshold (5/10/15%); Figure S7: distribution of the sugar-ring puckering (pseudorotation phase angle P); Table S11: sugar-ring puckering (structure × sugar North/South occupancies); Figure S8: periodic-boundary-corrected ligand RMSD and pose states; Table S12: pose states from the periodic-boundary-corrected ligand RMSD (structure-resolved); Table S13: geometric re-measurement of the opening (the two Tyr165-bridge cavity openings); Figure S9: cross-validation of the Cremer–Pople and the existing puckering methods; Table S14: independent cross-validation of the sugar-ring puckering (Cremer–Pople vs Altona–Sundaralingam); Figure S10: comparison of the glycosidic torsion χ and the sugar–base plane tilt between sugar types; Table S15: cross-species conservation of the active-site residues (*B. subtilis* 5EP8/5OLN vs *G. stearothermophilus* 1BRW); Figure S11: cross-species conservation of the active-site residues; Table S16: sugar-ring puckering by compound (North = C3′-endo occupancy); and Figure S12: effect of co-substrate phosphate on state-dependent ligand retention (PDF)

## References

[1] Hatano A., Matsuzaka R., Hamano R., Shimane G., Fukatsu T., Okumoto H., Kidowaki M., Konishi T. (2026). Impact of 6-Purine
Modifications on the Ribose Substrate Selectivity of Pyrimidine Nucleoside
Phosphorylase (PyNP) in Base Exchange Reactions. Carbohydr. Res..

[2] Lashkov, A. A. ; Balaev, V. V. ; Gabdoulkhakov, A. G. ; Betzel, C. ; Mikhailov, A. M. X-Ray Structure of the Complex Pyrimidine-Nucleoside Phosphorylase from Bacillus subtilis with Sulfate Ion; PDB ID 5EP8, RCSB Protein Data Bank, 2018. 10.2210/pdb5EP8/pdb. PMC5894104 29633966

[3] Balaev V. V., Prokofev I. I., Gabdoulkhakov A. G., Betzel C., Lashkov A. A. (2018). Crystal
Structure of Pyrimidine-Nucleoside Phosphorylase from *Bacillus
subtilis* in Complex with Imidazole and Sulfate. Acta Crystallogr., Sect. F:Struct. Biol. Commun..

[4] Pugmire M. J., Ealick S. E. (1998). The Crystal Structure
of Pyrimidine Nucleoside Phosphorylase
in a Closed Conformation. Struct..

[5] Hatano A., Wakana H., Terado N., Kojima A., Nishioka C., Iizuka Y., Imaizumi T., Uehara S. (2019). Bio-Catalytic Synthesis
of Unnatural Nucleosides Possessing a Large Functional Group Such
as a Fluorescent Molecule by Purine Nucleoside Phosphorylase. Catal. Sci. Technol..

[6] Hatano A., Shiraishi M., Terado N., Tanabe A., Fukuda K. (2015). Enzymatic
Synthesis and RNA Interference of Nucleosides Incorporating Stable
Isotopes into a Base Moiety. Bioorg. Med. Chem..

[7] Hatano A., Matsuzaka R., Shimane G., Wakana H., Suzuki K., Nishioka C., Kojima A., Kidowaki M. (2023). Introduction of Pseudo-Base
Benzimidazole Derivatives into Nucleosides via Base Exchange by a
Nucleoside Metabolic Enzyme. Bioorg. Med. Chem..

[8] Balaev V. V., Lashkov A. A., Prokofev I. I., Gabdulkhakov A. G., Seregina T. A., Mironov A. S., Betzel C., Mikhailov A. M. (2016). Substrate
Specificity of Pyrimidine Nucleoside Phosphorylases of the NP-II Family
Probed by X-ray Crystallography and Molecular Modeling. Crystallogr. Rep..

[9] Pettersen E. F., Goddard T. D., Huang C. C., Couch G. S., Greenblatt D. M., Meng E. C., Ferrin T. E. (2004). UCSF Chimera—A
Visualization
System for Exploratory Research and Analysis. J. Comput. Chem..

[10] Olsson M. H. M., Søndergaard C. R., Rostkowski M., Jensen J. H. (2011). PROPKA3: Consistent Treatment of Internal and Surface
Residues in Empirical pKa Predictions. J. Chem.
Theory Comput..

[11] Gao X.-F., Huang X.-R., Sun C.-C. (2006). Role of
Each Residue in Catalysis
in the Active Site of Pyrimidine Nucleoside Phosphorylase from *Bacillus subtilis*: A Hybrid QM/MM Study. J. Struct. Biol..

[12] Le
Guilloux V., Schmidtke P., Tuffery P. (2009). Fpocket: An Open Source
Platform for Ligand Pocket Detection. BMC Bioinf..

[13] el
Kouni M. H., Goudgaon N. M., Rafeeq M., Al Safarjalani O. N., Schinazi R. F., Naguib F. N. M. (2000). 5-Phenylthioacyclouridine: A Potent
and Specific Inhibitor of Uridine Phosphorylase. Biochem. Pharmacol..

[14] Hanwell M. D., Curtis D. E., Lonie D. C., Vandermeersch T., Zurek E., Hutchison G. R. (2012). Avogadro: An Advanced Semantic Chemical
Editor, Visualization, and Analysis Platform. J. Cheminf..

[15] Bayly C. I., Cieplak P., Cornell W., Kollman P. A. (1993). A Well-Behaved
Electrostatic
Potential Based Method Using Charge Restraints for Deriving Atomic
Charges: The RESP Model. J. Phys. Chem..

[16] Wang J., Wang W., Kollman P. A., Case D. A. (2006). Automatic Atom Type
and Bond Type Perception in Molecular Mechanical Calculations. J. Mol. Graphics Modell..

[17] Wang J., Wolf R. M., Caldwell J. W., Kollman P. A., Case D. A. (2004). Development
and Testing of a General Amber Force Field. J. Comput. Chem..

[18] Sousa
da Silva A. W., Vranken W. F. (2012). ACPYPE—AnteChamber PYthon
Parser interfacE. BMC Res. Notes.

[19] Abraham M. J., Murtola T., Schulz R., Páll S., Smith J. C., Hess B., Lindahl E. (2015). GROMACS: High Performance
Molecular Simulations through Multi-Level Parallelism from Laptops
to Supercomputers. SoftwareX.

[20] Eberhardt J., Santos-Martins D., Tillack A. F., Forli S. (2021). AutoDock Vina
1.2.0:
New Docking Methods, Expanded Force Field, and Python Bindings. J. Chem. Inf. Model..

[21] Trott O., Olson A. J. (2010). AutoDock Vina: Improving the Speed
and Accuracy of
Docking with a New Scoring Function, Efficient Optimization, and Multithreading. J. Comput. Chem..

[22] Lindorff-Larsen K., Piana S., Palmo K., Maragakis P., Klepeis J. L., Dror R. O., Shaw D. E. (2010). Improved Side-Chain
Torsion Potentials for the Amber ff99SB Protein Force Field. Proteins.

[23] Jorgensen W. L., Chandrasekhar J., Madura J. D., Impey R. W., Klein M. L. (1983). Comparison
of Simple Potential Functions for Simulating Liquid Water. J. Chem. Phys..

[24] Berendsen H. J. C., Postma J. P. M., van Gunsteren W. F., DiNola A., Haak J. R. (1984). Molecular
Dynamics with Coupling to an External Bath. J. Chem. Phys..

[25] Parrinello M., Rahman A. (1981). Polymorphic Transitions in Single Crystals: A New Molecular
Dynamics Method. J. Appl. Phys..

[26] Hess B., Bekker H., Berendsen H. J. C., Fraaije J. G. E. M. (1997). LINCS: A Linear
Constraint Solver for Molecular Simulations. J. Comput. Chem..

[27] Darden T., York D., Pedersen L. (1993). Particle Mesh
Ewald: An N·log­(N)
Method for Ewald Sums in Large Systems. J. Chem.
Phys..

[28] Valdés-Tresanco M. S., Valdés-Tresanco M. E., Valiente P. A., Moreno E. (2021). gmx_MMPBSA:
A New Tool to Perform End-State Free Energy Calculations with GROMACS. J. Chem. Theory Comput..

[29] Duan L., Liu X., Zhang J. Z. H. (2016). Interaction Entropy:
A New Paradigm for Highly Efficient
and Reliable Computation of Protein–Ligand Binding Free Energy. J. Am. Chem. Soc..

[30] Ekberg V., Ryde U. (2021). On the Use of Interaction Entropy and Related Methods to Estimate
Binding Entropies. J. Chem. Theory Comput..

[31] Fukushima M., Suzuki N., Emura T., Yano S., Kazuno H., Tada Y., Yamada Y., Asao T. (2000). Structure and Activity
of Specific Inhibitors of Thymidine Phosphorylase to Potentiate the
Function of Antitumor 2′-Deoxyribonucleosides. Biochem. Pharmacol..

[32] Norman R. A., Barry S. T., Bate M., Breed J., Colls J. G., Ernill R. J., Luke R. W. A., Minshull C. A., McAlister M. S. B., McCall E. J., McMiken H. H. J., Paterson D. S., Timms D., Tucker J. A., Pauptit R. A. (2004). Crystal Structure
of Human Thymidine
Phosphorylase in Complex with a Small Molecule Inhibitor. Struct..

[33] Bzowska A., Kulikowska E., Shugar D. (2000). Purine Nucleoside Phosphorylases:
Properties, Functions, and Clinical Aspects. Pharmacol. Ther..

[34] Pugmire M. J., Ealick S. E. (2002). Structural Analyses Reveal Two Distinct
Families of
Nucleoside Phosphorylases. Biochem. J..

[35] Kastner D. W., Luo W., Ho W., Reinhardt C. R., Keys A., Kulik H. J. (2026). QuantumPDB:
A Workflow for High-Throughput Quantum Cluster Model Generation from
Protein Structures. J. Chem. Inf. Model..

