# Supplementary material for: Conformation- and Compound-Dependence of Ribose/2′-Deoxyribose Retention Patterns in Pyrimidine-Nucleoside Phosphorylase from Bacillus subtilis: A Four-Structure Molecular Dynamics Study
Source: ACS Omega. 2026 Sep 12;11(38):58284–305. doi: 10.1021/acsomega.6c08499 (PMC13629066; doi:10.1021/acsomega.6c08499)
Supplement: Supplementary file 1 [file ao6c08499_si_001.pdf]

Supporting Information

Conformation- and Compound-Dependence of Ribose/2′-Deoxyribose Retention Patterns in Pyrimidine-Nucleoside Phosphorylase from Bacillus subtilis: A Four-Structure Molecular Dynamics Study

Akihiko Hatano[1],* and Kousei Takeshima[1]

[1] Course of Chemistry and Biotechnology, Program of Chemistry and Materials, College of Engineering, Shibaura Institute of Technology, 307 Fukasaku, Minuma-ku, Saitama-shi, Saitama 337-8570, Japan

* Corresponding author. E-mail: a-hatano@sic.shibaura-it.ac.jp

Contents

- Figure S1. Whole-molecule views (cartoon and surface) of the PyNP active-site pocket across the four conformational states (see Figure_S1_v6_full.pdf).

- Figure S2. Conformation- and ligand-class-resolved MM-PB(GB)SA decomposition.

- Table S1. Sphere-packing parameters for the pocket volume and validation against fpocket.

- Table S2. Per-system list of all 156 MD trajectories (with run times and quality flags).

- Figure S3. Robustness of the binding fraction to the COM–COM distance threshold (8, 10, 12 Å).

- Figure S4. Cross-platform reproducibility of the pattern-C binders in 5EP8orig (RCCS vs vast.ai).

- Table S3. Later-window (50–100 ns) per-residue contact fractions and the closed-versus-open comparison.

- Table S4. Statistical robustness of the closed-versus-open contrast against pseudo-replication (three methods).

- Table S5. Conformation-resolved ΔTOTAL_GB for all 13 ligands (non-monotonic affinity profile).

- Figure S5. Convergence of the cumulative binding fraction over 100 ns (representative systems plus panel-wide early/late and replica statistics).

- Figure S6. Stability of the backbone conformational state during MD (the Q153–Y165 lid-loop coordinate over 100 ns).

- Table S6. Distance-based close-contact occupancies between ligand polar atoms and active-site residues (5EP8orig).

- Table S7. AutoDock Vina docking scores and their divergence from MD retention.

- Table S8. Structure-resolved Tyr165 contact and the 1BRW-exclusion sensitivity analysis.

- Table S9. Composition of the simulation systems (representative system per structure).

- Table S10. Sensitivity of the pattern classification to the margin threshold (5/10/15 %).

- Figure S7. Distribution of the sugar-ring puckering (pseudorotation phase angle P).

- Table S11. Sugar-ring puckering (structure × sugar North/South occupancies).

- Figure S8. Periodic-boundary-corrected ligand RMSD and pose states.

- Table S12. Pose states from the periodic-boundary-corrected ligand RMSD (structure-resolved).

- Table S13. Geometric re-measurement of the opening (the two Tyr165-bridge cavity openings).

- Figure S9. Cross-validation of the Cremer–Pople and the existing puckering methods.

- Table S14. Independent cross-validation of the sugar-ring puckering (Cremer–Pople vs Altona–Sundaralingam).

- Figure S10. Comparison of the glycosidic torsion χ and the sugar–base plane tilt between sugar types.

- Table S15. Cross-species conservation of the active-site residues (*B. subtilis* 5EP8/5OLN vs *Geobacillus stearothermophilus* 1BRW).

- Figure S11. Cross-species conservation of the active-site residues.

- Table S16. Sugar-ring puckering by compound (North = C3′-endo occupancy).

- Figure S12. Effect of co-substrate phosphate on state-dependent ligand retention.

Figure S1. Whole-molecule views of the PyNP active-site pocket across the four conformational states.

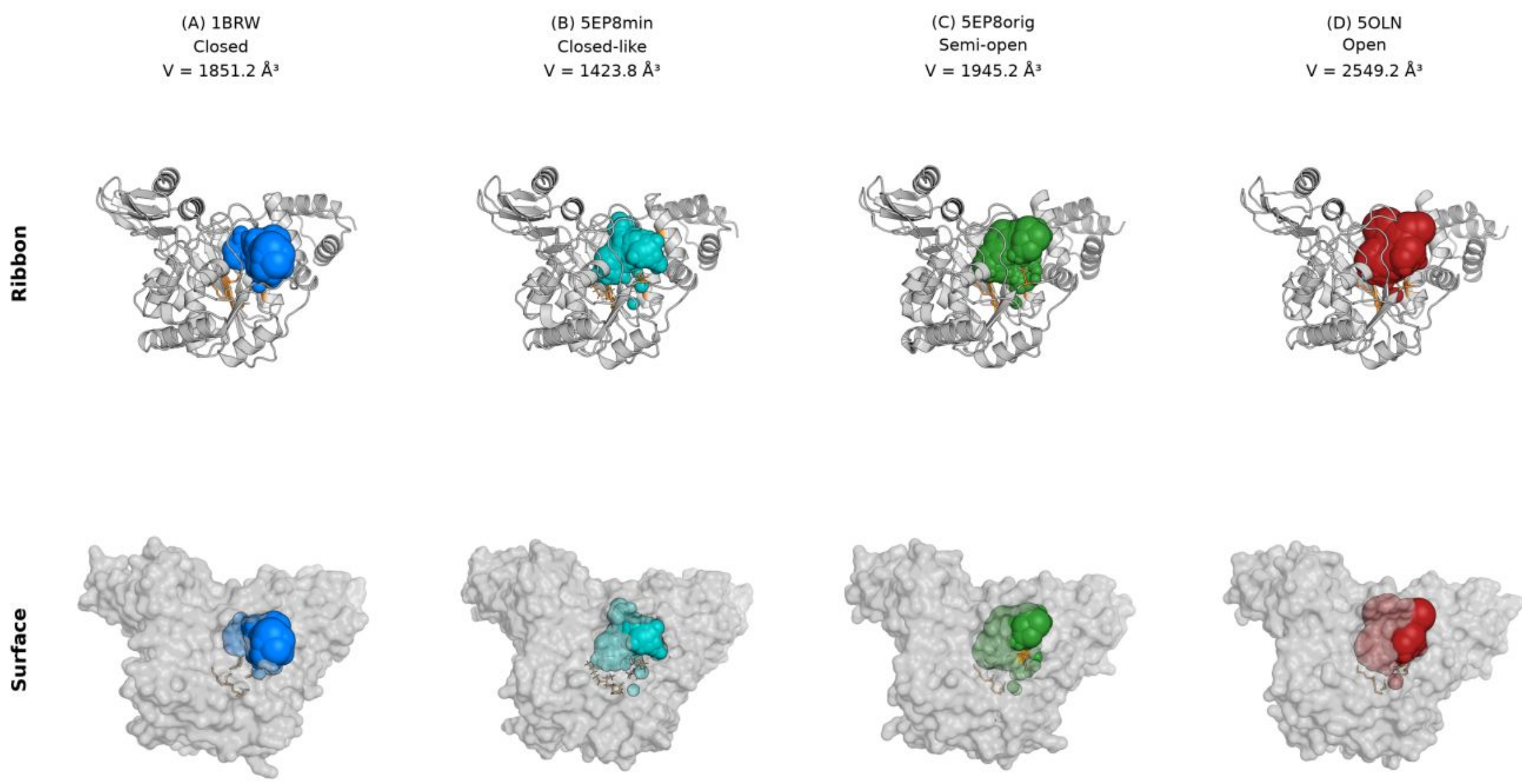

The sphere-packing pocket envelope (v6 reference; Methods §2.3) is shown superimposed on the four crystal structures of PyNP. All structures were superimposed on 1BRW with the matchmaker tool of UCSF ChimeraX (ref S1) and are shown from a common viewpoint. Top row: cartoon-ribbon representation with the α7–α8 gate loop (residues 153–170) shown in grey in all panels. Bottom row: semi-transparent surface representation from the same viewpoint. The pocket spheres are colored by state, and the seven active-site residues (D80, K81, H82, K108, Q153, Y165, K188) are shown as orange sticks in all panels. (A) 1BRW (closed state, V = 1851.2 Å^3); (B) 5EP8min (closed-like state, V = 1423.8 Å^3); (C) 5EP8orig (semi-open state, V = 1945.2 Å^3); (D) 5OLN (open state, V = 2549.2 Å^3). The columns are arranged in the same order as Table 1. The pocket volume expands monotonically about 1.4-fold from the closed state (A) to the open state (D).

Table S1. Sphere-packing parameters for the pocket volume and a three-method comparison.

A. Algorithm parameters (sphere-packing v6)

Parameter	Value
r_min	1.4 Å
r_max	4.5 Å
search_radius	15.0 Å
grid_spacing	0.7 Å
min_buried_fraction	0.40
max_polar_combined	0.85
min_hydrophobic	0.10
required_contact	≥ 1 of {D80,H82,K108,Q153,Y165,K188}
line-of-sight rule	BFS from the nearest accepted sphere
anchor seeds	1 centroid + 6 inner-offset

B. Pocket volume — three-method comparison

PDB code	Conformation	fpocket (7-residue coverage, Å³)	Sphere v1 (unconstrained, Å³)	Sphere v6 (strict, Å³)
1BRW	Closed	1040.8	4649.7	1851.2
5EP8min	Closed-like	1102.4	4239.8	1423.8
5EP8orig	Semi-open	1056.0	6692.6	1945.2
5OLN	Open	1739.2	7768.3	2549.2
Ratio (5OLN / 1BRW)		1.67	1.67	1.38

Notes:

• The methods agree only on the point that the open state (5OLN) has the largest active-site pocket and that it is expanded roughly 1.4–1.7-fold relative to the closed 1BRW (5OLN/1BRW ratio 1.38–

1.67). The detailed rank ordering of the four structures is method-dependent: the in-house sphere-packing order (5EP8min < 1BRW < 5EP8orig < 5OLN) is reproduced only by its own unconstrained variant v1 (Spearman $\rho = 1.0$, two settings of the same algorithm), whereas fpocket gives a different order (1BRW < 5EP8orig < 5EP8min < 5OLN; Spearman $\rho = 0.4$ between fpocket and sphere v6). Only the finding that 5OLN is the largest and the closed→open expansion are method-independent.

• fpocket counts only spheres co-localized with the seven active-site residues and therefore assigns a small absolute volume.

• Sphere-packing v1 is unconstrained and overestimates the cavity by leaking into surface clefts.

• Sphere-packing v6 (strict, this study) is reported in Table 1 and underlies Figure 2.

• The Cα distance is an independent geometric marker; Q153–Y165 increases monotonically (+5.12 Å), supporting the lid-loop opening interpretation.

Section A. Parameters of the in-house sphere-packing algorithm (v6: BFS expansion with multi-anchor seeding). The ten algorithm parameters (the lower and upper sphere radii, the search radius, the grid spacing, the burial-fraction threshold, the polar/hydrophobic neighborhood requirements, the required active-site contact, the line-of-sight rule, and the seeding strategy) are listed with their values and rationale.

Section B. Pocket volumes determined by three independent methods: (i) fpocket with a seven-residue coverage filter; (ii) sphere-packing v1 (unconstrained, an overestimating reference); (iii) sphere-packing v6 (the strict criteria used in main-text Table 1). Two Cα–Cα distances (Gln153–Tyr165 and Lys108–Tyr165) are reported as independent geometric markers of the conformational opening. All three methods agree on the two conformational endpoints — the fully open 5OLN is the largest cavity and gives a consistent 5OLN/1BRW volume ratio across methods (fpocket 1.67, sphere v1 1.67, sphere v6 1.38) — and the two sphere-packing variants rank all four states identically (Spearman $\rho = 1.00$ between v1 and v6). fpocket, however, did not resolve the intermediate states for this particular pocket: it compressed the three non-fully-open structures into a narrow 1041–1102 Å^3 band (1BRW 1040.8, 5EP8orig 1056.0, 5EP8min 1102.4 Å^3) and even placed the closed-like 5EP8min above the semi-open 5EP8orig, so its rank correlation with sphere-packing v6 was only $\rho = 0.40$. This behavior is consistent with fpocket being optimized for detecting and scoring surface-exposed druggable pockets rather than for measuring the graded volume of a single, largely buried active-site cavity partly bounded by a mobile gate loop; for the present four-state comparison it lacked the discrimination needed to place the intermediate states. We therefore used the geometry-based sphere-packing algorithm, which is anchored to the seven conserved active-site residues and reproduces the full closed→open volume ladder, as the primary volume metric, and report fpocket and the two Cα–Cα opening markers (Gln153–Tyr165 and Lys108–Tyr165) alongside it as independent checks that agree on the closed-to-open endpoints.

Note (sphere-packing v6 sphere counts N): 1BRW 54 / 5EP8min 47 / 5EP8orig 58 / 5OLN 61. This table was moved here from main-text §3.1 (former main-text Table 1, sphere-packing v6 volumes).

Figure S2. Conformation- and ligand-class-resolved MM-PB(GB)SA decomposition.

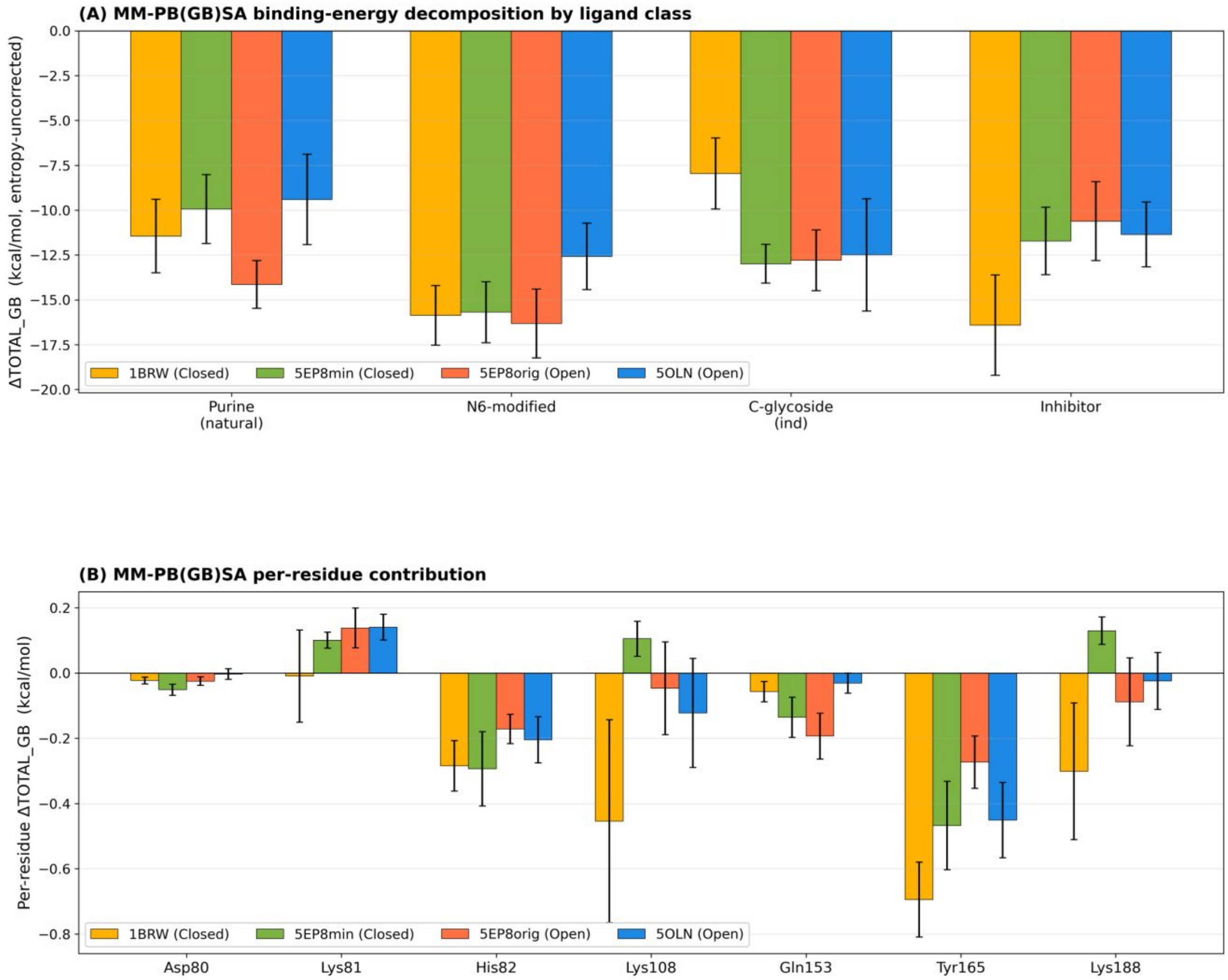

(A) Binding-energy decomposition by ligand class. The mean ΔTOTAL_GB (entropy-uncorrected) averaged over the compounds within each class is shown for each conformation. Ligand classes: native purines (H-rR, H-dR); N6-modified analogues (dmap, mmap, dbap pairs); C-glycosides (ind-rR, ind-dR); inhibitors (formycin B, TPI, PIA). Error bars: standard error of the mean.

(B) Per-residue MM-PB(GB)SA contributions. The mean per-residue ΔTOTAL_GB of each active-site residue is shown separated by conformation. The HIS82 and HIE82 protonation variants are aggregated. Error bars: standard error of the mean. Both panels were computed from the Phase C cohort (n = 153 systems: 1BRW 39, 5EP8min 39, 5EP8orig 36, 5OLN 39). The interaction-entropy correction was omitted because the per-frame interaction-entropy standard deviation σ(IE) exceeded the reliability threshold of 3.6 kcal/mol (15 kJ/mol) in 152 of 153 systems (99 %) (Methods §2.8). The per-residue values use atomic contributions to ΔTOTAL_GB.

Table S2. List of all 156 production MD trajectories.

#	Structure	Conformation	Compound	Replicate	Run length (ns)	Status	Phase C MM-PBSA	Notes
1	1BRW	Closed	H-rR	rep1	100	Loose	✓	
2	1BRW	Closed	H-rR	rep2	100	Dissoc	✓	
3	1BRW	Closed	H-rR	rep3	100	Bound	✓	
4	1BRW	Closed	H-dR	rep1	100	Bound	✓	
5	1BRW	Closed	H-dR	rep2	100	Bound	✓	
6	1BRW	Closed	H-dR	rep3	100	Bound	✓	
7	1BRW	Closed	dmap-rR	rep1	100	Bound	✓	
8	1BRW	Closed	dmap-rR	rep2	100	Bound	✓	
9	1BRW	Closed	dmap-rR	rep3	100	Bound	✓	
10	1BRW	Closed	dmap-dR	rep1	100	Bound	✓	
11	1BRW	Closed	dmap-dR	rep2	100	Loose	✓	
12	1BRW	Closed	dmap-dR	rep3	100	Bound	✓	
13	1BRW	Closed	mmap-rR	rep1	100	Bound	✓	
14	1BRW	Closed	mmap-rR	rep2	100	Bound	✓	
15	1BRW	Closed	mmap-rR	rep3	100	Bound	✓	
16	1BRW	Closed	mmap-dR	rep1	100	Bound	✓	
17	1BRW	Closed	mmap-dR	rep2	100	Bound	✓	
18	1BRW	Closed	mmap-dR	rep3	100	Dissoc	✓	
19	1BRW	Closed	ind-rR	rep1	100	Loose	✓	
20	1BRW	Closed	ind-rR	rep2	100	Bound	✓	
21	1BRW	Closed	ind-rR	rep3	100	Loose	✓	
22	1BRW	Closed	ind-dR	rep1	100	Dissoc	✓	
23	1BRW	Closed	ind-dR	rep2	100	Loose	✓	
24	1BRW	Closed	ind-dR	rep3	100	Loose	✓	

25	1BRW	Closed	dbap-rR	rep1	100	Bound	✓
26	1BRW	Closed	dbap-rR	rep2	100	Bound	✓
27	1BRW	Closed	dbap-rR	rep3	100	Bound	✓
28	1BRW	Closed	dbap-dR	rep1	100	Bound	✓
29	1BRW	Closed	dbap-dR	rep2	100	Bound	✓
30	1BRW	Closed	dbap-dR	rep3	100	Bound	✓
31	1BRW	Closed	FormycinB	rep1	100	Bound	✓
32	1BRW	Closed	FormycinB	rep2	100	Bound	✓
33	1BRW	Closed	FormycinB	rep3	100	Bound	✓
34	1BRW	Closed	TPI	rep1	100	Bound	✓
35	1BRW	Closed	TPI	rep2	100	Bound	✓
36	1BRW	Closed	TPI	rep3	100	Bound	✓
37	1BRW	Closed	PIA	rep1	100	Bound	✓
38	1BRW	Closed	PIA	rep2	100	Bound	✓
39	1BRW	Closed	PIA	rep3	100	Bound	✓
40	5EP8min	Closed	H-rR	rep1	100	Dissoc	✓
41	5EP8min	Closed	H-rR	rep2	100	Bound	✓
42	5EP8min	Closed	H-rR	rep3	100	Bound	✓
43	5EP8min	Closed	H-dR	rep1	100	Bound	✓
44	5EP8min	Closed	H-dR	rep2	100	Bound	✓
45	5EP8min	Closed	H-dR	rep3	100	Bound	✓
46	5EP8min	Closed	dmap-rR	rep1	100	Bound	✓
47	5EP8min	Closed	dmap-rR	rep2	100	Bound	✓
48	5EP8min	Closed	dmap-rR	rep3	100	Bound	✓
49	5EP8min	Closed	dmap-dR	rep1	100	Bound	✓
50	5EP8min	Closed	dmap-dR	rep2	100	Loose	✓
51	5EP8min	Closed	dmap-dR	rep3	100	Bound	✓

52	5EP8min	Closed	mmap-rR	rep1	100	Bound	✓
53	5EP8min	Closed	mmap-rR	rep2	100	Bound	✓
54	5EP8min	Closed	mmap-rR	rep3	100	Bound	✓
55	5EP8min	Closed	mmap-dR	rep1	100	Dissoc	✓
56	5EP8min	Closed	mmap-dR	rep2	100	Loose	✓
57	5EP8min	Closed	mmap-dR	rep3	100	Bound	✓
58	5EP8min	Closed	ind-rR	rep1	100	Loose	✓
59	5EP8min	Closed	ind-rR	rep2	100	Bound	✓
60	5EP8min	Closed	ind-rR	rep3	100	Bound	✓
61	5EP8min	Closed	ind-dR	rep1	100	Bound	✓
62	5EP8min	Closed	ind-dR	rep2	100	Bound	✓
63	5EP8min	Closed	ind-dR	rep3	100	Loose	✓
64	5EP8min	Closed	dbap-rR	rep1	100	Bound	✓
65	5EP8min	Closed	dbap-rR	rep2	100	Bound	✓
66	5EP8min	Closed	dbap-rR	rep3	100	Loose	✓
67	5EP8min	Closed	dbap-dR	rep1	100	Bound	✓
68	5EP8min	Closed	dbap-dR	rep2	100	Bound	✓
69	5EP8min	Closed	dbap-dR	rep3	100	Bound	✓
70	5EP8min	Closed	FormycinB	rep1	100	Bound	✓
71	5EP8min	Closed	FormycinB	rep2	100	Bound	✓
72	5EP8min	Closed	FormycinB	rep3	100	Bound	✓
73	5EP8min	Closed	TPI	rep1	100	Loose	✓
74	5EP8min	Closed	TPI	rep2	100	Dissoc	✓
75	5EP8min	Closed	TPI	rep3	100	Dissoc	✓
76	5EP8min	Closed	PIA	rep1	100	Bound	✓
77	5EP8min	Closed	PIA	rep2	100	Bound	✓
78	5EP8min	Closed	PIA	rep3	100	Bound	✓

79	5EP8orig	Open	H-rR	rep1	100	Loose	✓	
80	5EP8orig	Open	H-rR	rep2	100	Bound	✓	
81	5EP8orig	Open	H-rR	rep3	100	Dissoc	✓	rep3 generated on vast.ai (substrate panel)
82	5EP8orig	Open	H-dR	rep1	100	Loose	✓	
83	5EP8orig	Open	H-dR	rep2	100	Bound	✓	
84	5EP8orig	Open	H-dR	rep3	100	Bound	✓	rep3 generated on vast.ai (substrate panel)
85	5EP8orig	Open	dmap-rR	rep1	100	Bound	✓	
86	5EP8orig	Open	dmap-rR	rep2	100	Bound	✓	
87	5EP8orig	Open	dmap-rR	rep3	100	Bound	✓	rep3 generated on vast.ai (substrate panel)
88	5EP8orig	Open	dmap-dR	rep1	100	Loose	✓	
89	5EP8orig	Open	dmap-dR	rep2	100	Loose	✓	
90	5EP8orig	Open	dmap-dR	rep3	100	Loose	✓	rep3 generated on vast.ai (substrate panel)
91	5EP8orig	Open	mmap-rR	rep1	100	Bound	✓	
92	5EP8orig	Open	mmap-rR	rep2	100	Bound	✓	
93	5EP8orig	Open	mmap-rR	rep3	100	Dissoc	✓	rep3 generated on vast.ai (substrate panel)
94	5EP8orig	Open	mmap-dR	rep1	100	Bound	✓	

95	5EP8orig	Open	mmap-dR	rep2	100	Bound	✓	
96	5EP8orig	Open	mmap-dR	rep3	100	Loose	✓	rep3 generated on vast.ai (substrate panel)
97	5EP8orig	Open	ind-rR	rep1	100	Dissoc	✓	
98	5EP8orig	Open	ind-rR	rep2	100	Loose	✓	
99	5EP8orig	Open	ind-rR	rep3	100	Dissoc	✓	rep3 generated on vast.ai (substrate panel)
100	5EP8orig	Open	ind-dR	rep1	100	Bound	✓	
101	5EP8orig	Open	ind-dR	rep2	100	Bound	✓	
102	5EP8orig	Open	ind-dR	rep3	100	Bound	✓	rep3 generated on vast.ai (substrate panel)
103	5EP8orig	Open	dbap-rR	rep1	100	Bound	✓	
104	5EP8orig	Open	dbap-rR	rep2	100	Bound	✓	
105	5EP8orig	Open	dbap-rR	rep3	100	Bound	✓	rep3 generated on vast.ai (substrate panel)
106	5EP8orig	Open	dbap-dR	rep1	100	Bound	✓	
107	5EP8orig	Open	dbap-dR	rep2	100	Bound	✓	
108	5EP8orig	Open	dbap-dR	rep3	100	Bound	✓	rep3 generated on vast.ai (substrate panel)
109	5EP8orig	Open	FormycinB	rep1	100	Bound	✓	
110	5EP8orig	Open	FormycinB	rep2	100	Loose	✓	

111	5EP8orig	Open	FormycinB	rep3	100	Dissoc	—
112	5EP8orig	Open	TPI	rep1	100	Dissoc	✓
113	5EP8orig	Open	TPI	rep2	100	Loose	✓
114	5EP8orig	Open	TPI	rep3	100	Dissoc	—
115	5EP8orig	Open	PIA	rep1	100	Bound	✓
116	5EP8orig	Open	PIA	rep2	100	Bound	✓
117	5EP8orig	Open	PIA	rep3	100	Bound	—
118	5OLN	Open	H-rR	rep1	100	Loose	✓
119	5OLN	Open	H-rR	rep2	100	Loose	✓
120	5OLN	Open	H-rR	rep3	100	Dissoc	✓
121	5OLN	Open	H-dR	rep1	100	Loose	✓
122	5OLN	Open	H-dR	rep2	100	Bound	✓
123	5OLN	Open	H-dR	rep3	100	Loose	✓
124	5OLN	Open	dmap-rR	rep1	100	Dissoc	✓
125	5OLN	Open	dmap-rR	rep2	100	Bound	✓
126	5OLN	Open	dmap-rR	rep3	100	Loose	✓
127	5OLN	Open	dmap-dR	rep1	100	Loose	✓
128	5OLN	Open	dmap-dR	rep2	100	Loose	✓
129	5OLN	Open	dmap-dR	rep3	100	Bound	✓
130	5OLN	Open	mmap-rR	rep1	100	Bound	✓
131	5OLN	Open	mmap-rR	rep2	100	Bound	✓
132	5OLN	Open	mmap-rR	rep3	100	Bound	✓
133	5OLN	Open	mmap-dR	rep1	100	Bound	✓
134	5OLN	Open	mmap-dR	rep2	100	Dissoc	✓
135	5OLN	Open	mmap-dR	rep3	100	Bound	✓
136	5OLN	Open	ind-rR	rep1	100	Loose	✓
137	5OLN	Open	ind-rR	rep2	100	Loose	✓

138	5OLN	Open	ind-rR	rep3	100	Bound	✓
139	5OLN	Open	ind-dR	rep1	100	Dissoc	✓
140	5OLN	Open	ind-dR	rep2	100	Dissoc	✓
141	5OLN	Open	ind-dR	rep3	100	Bound	✓
142	5OLN	Open	dbap-rR	rep1	100	Loose	✓
143	5OLN	Open	dbap-rR	rep2	100	Bound	✓
144	5OLN	Open	dbap-rR	rep3	100	Bound	✓
145	5OLN	Open	dbap-dR	rep1	100	Bound	✓
146	5OLN	Open	dbap-dR	rep2	100	Dissoc	✓
147	5OLN	Open	dbap-dR	rep3	100	Bound	✓
148	5OLN	Open	FormycinB	rep1	100	Loose	✓
149	5OLN	Open	FormycinB	rep2	100	Bound	✓
150	5OLN	Open	FormycinB	rep3	100	Bound	✓
151	5OLN	Open	TPI	rep1	100	Dissoc	✓
152	5OLN	Open	TPI	rep2	100	Bound	✓
153	5OLN	Open	TPI	rep3	100	Dissoc	✓
154	5OLN	Open	PIA	rep1	100	Bound	✓
155	5OLN	Open	PIA	rep2	100	Bound	✓
156	5OLN	Open	PIA	rep3	100	Bound	✓

State counts (replicate-averaged states from MASTER_v7):

Bound: 102 / 156

Dissoc: 22
/ 156

Loose: 32
/ 156

(Here, the two-group closed/open classification follows the statistical grouping of §2.8 [closed = 1BRW, 5EP8min; open = 5EP8orig, 5OLN] and is coarser than the four-state labels closed / closed-like / semi-open / open used elsewhere.)

The complete list of all production trajectories used in this study, organized by structure (rows are band-colored: blue for the closed structures [1BRW, 5EP8min], orange for the open structures [5EP8orig, 5OLN]). Columns: row index; structure (PDB code); conformational class (closed/open); compound; replica number; production run time (ns); binding state at the end of the run (Bound/Loose/Dissoc, color-coded green/amber/red, defined by the binding-fraction–minimum-distance landscape of main-text Figure 3); Phase C MM-PB(GB)SA completion flag (✓ or —); a note on provenance. Total: 13 compounds × 4 conformations × 3 replicas = 156 trajectories, each 100 ns (15.6 µs of cumulative sampling). The aggregate state counts are summarized as a table at the bottom of the sheet.

Figure S3. Robustness of the binding fraction to the COM–COM distance threshold.

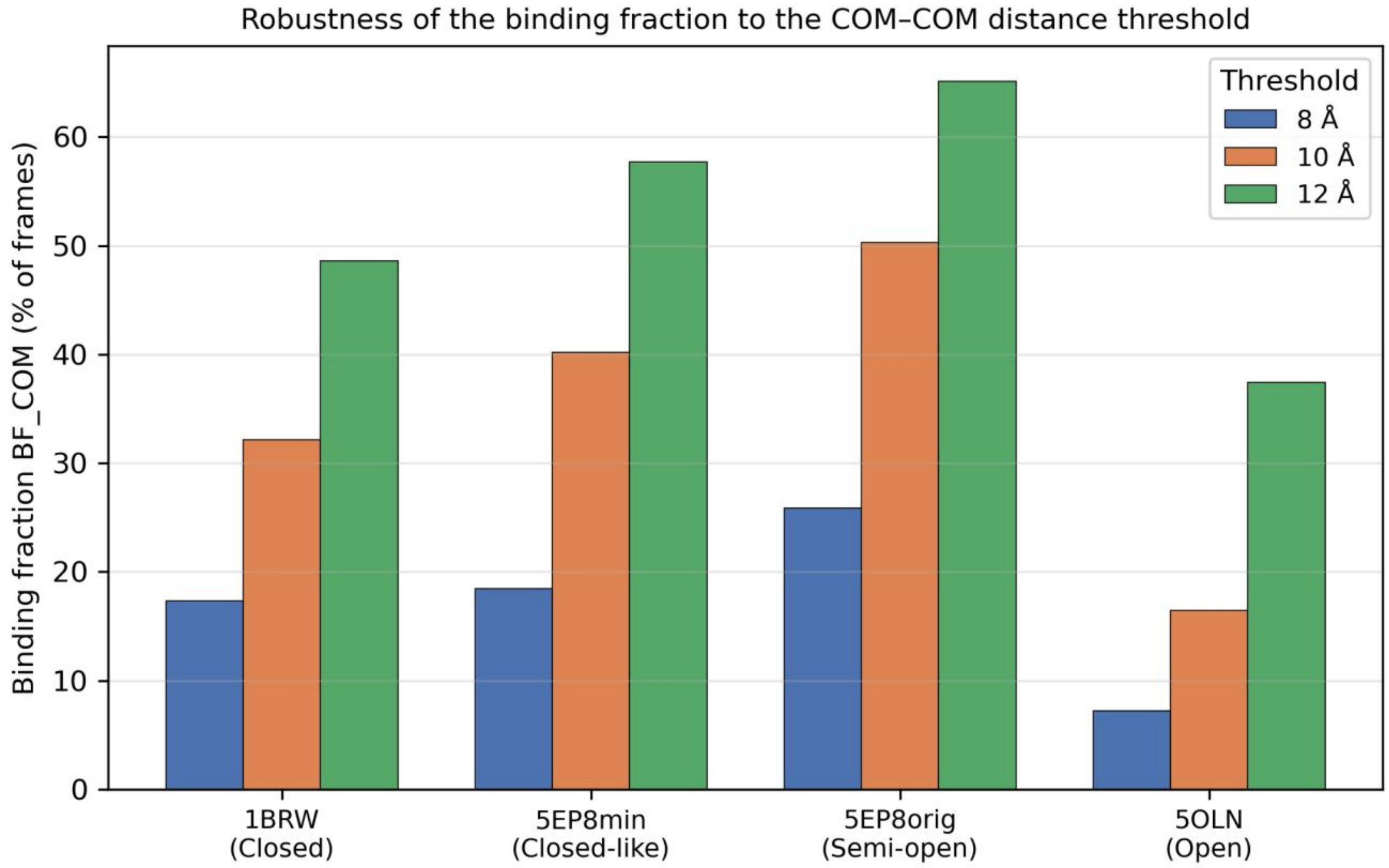

The Closed > Semi-open > Open ordering of the two open structures vs 1BRW/5EP8min is preserved at 8, 10, and 12 Å.

The structure-averaged binding fraction (BF_COM, the fraction of frames in which the ligand center of mass is within a given distance of the seven-residue active-site centroid) was computed at thresholds of 8, 10, and 12 Å for each of the four conformational states. The semi-open 5EP8orig structure showed the highest binding fraction at all thresholds, and 5OLN the lowest. The relative order of the structures was preserved at all three cutoffs, confirming that the 10 Å threshold used in the main text does not bias the conformational comparison.

Figure S4. Cross-platform reproducibility of the pattern-C binders in 5EP8orig.

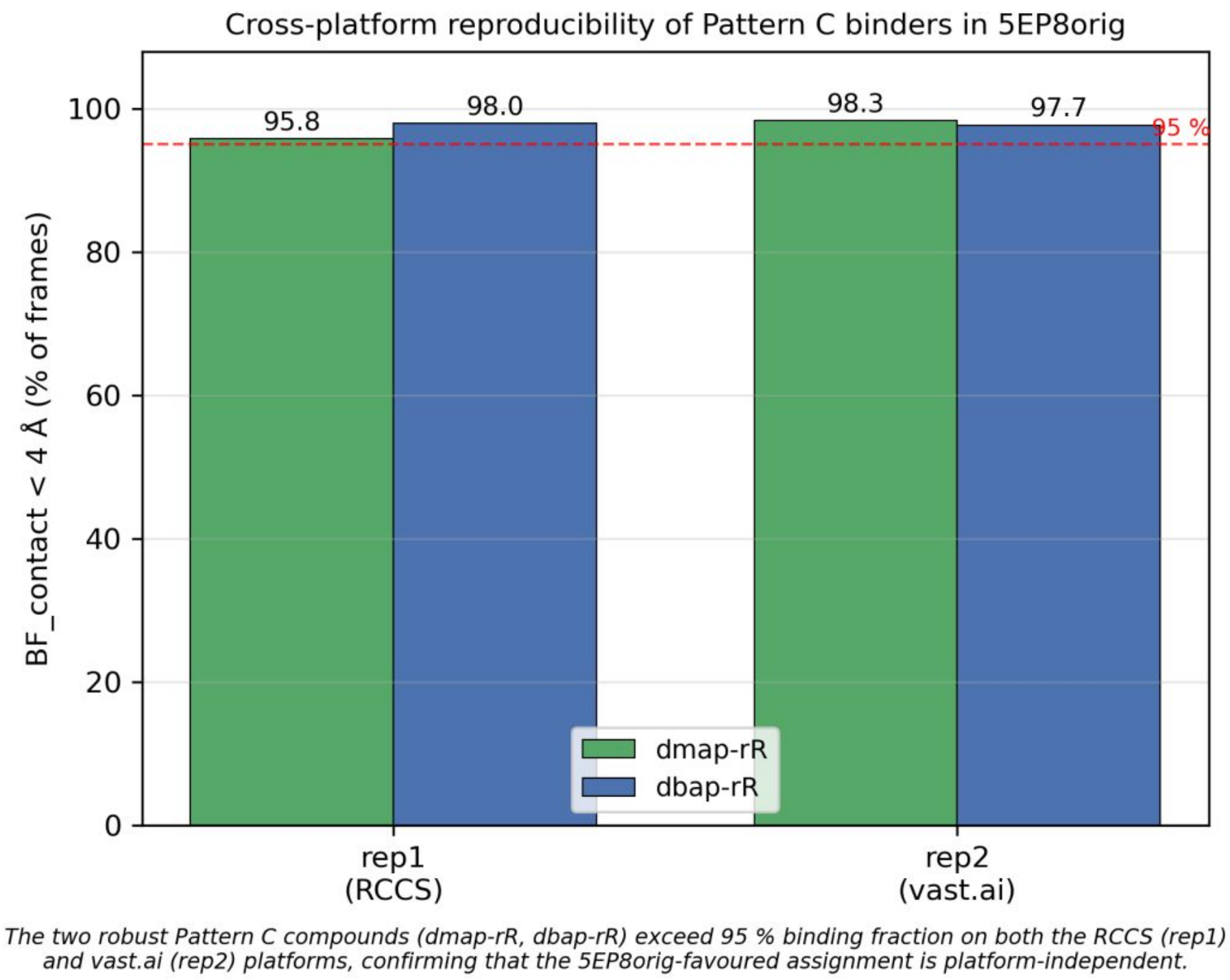

Replica-resolved atomic contact fractions (BF_contact < 4 Å, full 0–100 ns) for the two robust pattern-C compounds, dmap-rR and dbap-rR, in the 5EP8orig structure. rep1 was generated on the RCCS supercomputer, and the remaining replicas (rep2 and rep3) on vast.ai cloud GPUs. Both compounds exceeded a binding fraction of 95 % (dashed line, 95 %) on both computing platforms, confirming that the 5EP8orig-preferring (pattern-C) assignment of these strong binders is reproducible and platform-independent. Marginally or intermittently binding compounds are not expected to be reproduced at the single-trajectory level, which is why all main-text metrics are reported as replica averages.

Table S3. Later-window (50–100 ns) per-residue contact fractions: closed versus open.

Residue	Closed (mean ± SEM, %)	Open (mean ± SEM, %)	Δ (pp)	p (significance)
Asp80	2.2 ± 1.3	2.2 ± 1.1	+0.0	0.658 (ns)
Lys81	8.5 ± 2.8	9.6 ± 2.3	-1.1	0.266 (ns)
His82	30.0 ± 3.9	20.1 ± 3.3	+9.9	0.046 (*)
Lys108	10.9 ± 3.0	9.5 ± 2.7	+1.4	0.702 (ns)
Gln153	21.8 ± 3.7	19.2 ± 3.8	+2.6	0.164 (ns)
Tyr165	43.4 ± 4.3	22.6 ± 3.7	+20.9	3.5e-05 (***)
Lys188	12.5 ± 3.3	11.9 ± 2.9	+0.5	0.776 (ns)

The atomic contact fraction (BF_contact < 4 Å) of each active-site residue was averaged over the equilibrated later window (50–100 ns) and over the closed (1BRW + 5EP8min, n = 78) and open (5EP8orig + 5OLN, n = 78) trajectory groups (mean ± SEM). Δ = closed − open (percentage points); p is from the two-sided Mann–Whitney U test. Tyr165 is the only residue that retains a strong, highly significant closed-state-specific contact in the later window (Δ = +20.9 pp, $p = 3.5\times10^{-5}$), consistent with the full-window result of main-text Table 3. Significance: *** $p < 0.001$, ** $p < 0.01$, * $p < 0.05$, ns $p \geq 0.05$.

Table S4. Statistical robustness of the closed-versus-open per-residue contact difference against pseudo-replication.

Residue	(i) Per-trajectory MWU, p	(ii) Ligand-paired Wilcoxon: Δ (pp), p	(iii) Mixed effects: coefficient, p	Consistent?
Asp80	0.008 (**)	+0.1, 0.455 (ns)	-0.15, 0.920 (ns)	no
Lys81	0.074 (ns)	-4.3, 0.146 (ns)	+4.27, 0.235 (ns)	no
His82	0.011 (*)	+11.2, 0.017 (*)	-11.23, 0.016 (*)	yes
Lys108	0.430 (ns)	-1.0, 0.735 (ns)	+0.98, 0.779 (ns)	no
Gln153	0.213 (ns)	+2.1, 0.839 (ns)	-2.11, 0.648 (ns)	no
Tyr165	1.2e-05 (***)	+21.3, 1.7e-3 (**)	-21.28, 7.9e-07 (***)	yes
Lys188	0.344 (ns)	+0.1, 0.839 (ns)	-0.11, 0.979 (ns)	no

The per-residue atomic contact fraction (BF_contact < 4 Å, full 0–100 ns) was compared between the closed (1BRW + 5EP8min) and open (5EP8orig + 5OLN) groups by three methods of progressively stricter treatment of non-independence: (i) a naïve per-trajectory Mann–Whitney U test treating all 78 + 78 trajectories as independent; (ii) a ligand-level paired Wilcoxon signed-rank test over the 13 compounds, in which each compound provides one closed-group mean and one open-group mean (n = 13 truly independent units); and (iii) a linear mixed-effects model with compound as a random intercept, BF ~ group + (1 | compound). Tyr165 is the only residue strongly and significantly closed-state-specific by all three methods; His82 is significant but weaker; the remaining five residues are consistently non-significant. Significance markers: *** $p < 0.001$, ** $p < 0.01$, * $p < 0.05$, ns $p \geq 0.05$.

Table S5. Conformation-resolved MM-PB(GB)SA binding energies (ΔTOTAL_GB) for all 13 ligands.

Ligand	1BRW	5EP8min	5EP8orig	5OLN	Best (Δ vs 2nd, z)
FormycinB	-14.0 ± 1.6	-12.0 ± 2.8	-10.3 ± 0.2	-11.3 ± 3.7	1BRW (Δ1.9, z=0.60)
H-dR	-12.5 ± 1.5	-9.8 ± 1.1	-16.2 ± 2.0	-13.4 ± 4.0	5EP8orig (Δ2.8, z=0.64)
H-rR	-10.4 ± 4.7	-10.1 ± 4.6	-12.1 ± 1.6	-5.4 ± 2.5	5EP8orig (Δ1.7, z=0.35)
PIA	-27.1 ± 1.7	-17.6 ± 0.7	-16.8 ± 1.8	-13.1 ± 4.1	1BRW (Δ9.5, z=5.06) *
TPI	-8.2 ± 2.6	-5.6 ± 1.8	-4.8 ± 3.5	-9.6 ± 3.3	5OLN (Δ1.4, z=0.33)
dbap-dR	-19.0 ± 2.3	-22.4 ± 2.4	-26.1 ± 6.8	-11.6 ± 4.3	5EP8orig (Δ3.6, z=0.50)
dbap-rR	-23.3 ± 2.0	-23.0 ± 4.1	-25.3 ± 1.6	-23.8 ± 2.8	5EP8orig (Δ1.5, z=0.45)
dmap-dR	-10.7 ± 3.0	-14.0 ± 2.6	-10.8 ± 1.2	-14.0 ± 5.0	5EP8min (Δ0.0, z=0.00)
dmap-rR	-16.9 ± 6.6	-18.5 ± 2.8	-10.4 ± 0.8	-6.2 ± 2.6	5EP8min (Δ1.6, z=0.23)
ind-dR	-5.5 ± 3.2	-12.4 ± 2.4	-16.5 ± 1.0	-8.2 ± 5.9	5EP8orig (Δ4.1, z=1.56)
ind-rR	-10.4 ± 2.7	-13.6 ± 0.8	-9.1 ± 1.5	-16.7 ± 2.5	5OLN (Δ3.2, z=1.18)
mmap-rR	-12.7 ± 2.4	-10.2 ± 1.2	-13.8 ± 3.2	-7.3 ± 0.4	5EP8orig (Δ1.1, z=0.28)
mmap-dR	-12.7 ± 5.0	-5.9 ± 0.5	-11.4 ± 1.8	-12.5 ± 5.2	1BRW (Δ0.2, z=0.03)

The mean generalized-Born endpoint binding energy ΔTOTAL_GB of each ligand across the four conformational states (kcal/mol, entropy-uncorrected; n = 3 replicas per structure, except that three 5EP8orig × inhibitor systems have n = 2). The most favorable (most negative) value for each ligand is

shown in the "argmin" column. For 9 of 13 ligands, the most favorable binding energy occurred in an interior (non-extreme) conformation along the closure ladder 5EP8min < 1BRW < 5EP8orig < 5OLN, and for 6 ligands it occurred specifically in the semi-open 5EP8orig structure. This shows that the non-monotonic affinity profile highlighted for dbap-dR in the main text is not isolated but a recurring pattern, although it should be read cautiously given the entropy-uncorrected, single-trajectory endpoint nature of the GB estimate. Values are means ± SEM across replicas ($n = 3$, or $n = 2$ for the three 5EP8orig × inhibitor systems); the "Best" column gives the most favorable structure, the energy difference from its second-best structure (Δ, kcal/mol), and the standardized difference $z = \Delta / \sqrt{(SEM\_best^2 + SEM\_2nd^2)}$. Only 1 of 13 ligands (PIA) shows a most-favorable structure statistically distinguishable from its second-best ($z \geq 1.96$), and for PIA that optimal structure is the closed 1BRW rather than a semi-open state. For the remaining 12 ligands the differences between structures are within replica uncertainty, so the apparent non-monotonic/interior-optimum pattern cannot be separated from noise at the single-trajectory, entropy-uncorrected GB level and should be regarded as, at most, a weak qualitative trend.

* Most favorable in an interior (non-extreme) conformation.

Figure S5. Convergence of the cumulative binding fraction over 100 ns.

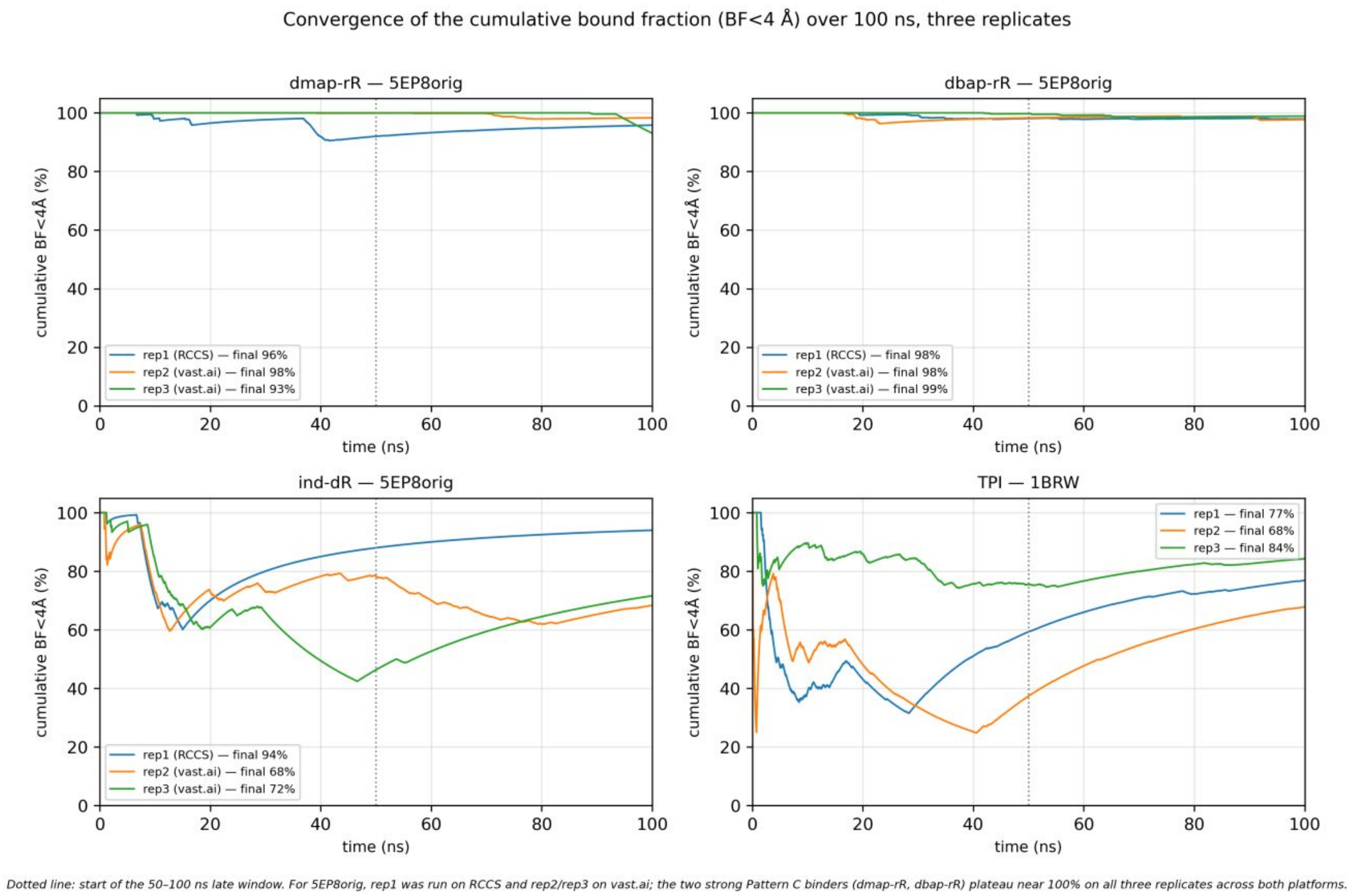

The cumulative binding fraction as a function of simulation time (the fraction of frames up to time t in which the ligand is within 4 Å of the seven-residue active site, in percent) is shown for four representative systems and their replicas. The two strong pattern-C binders (dmap-rR and dbap-rR in 5EP8orig) reach a plateau within the first few tens of nanoseconds and are well converged. In contrast, marginally or intermittently binding systems (for example, ind-dR in 5EP8orig and TPI in 1BRW) are still drifting over the 100 ns window, with pronounced inter-replica variability. For the 5EP8orig substrate systems all three replicas are available, with rep1 generated on the RCCS supercomputer and rep2/rep3 on vast.ai cloud GPUs. The two strong pattern-C binders reach a binding fraction > 90 % in all three replicas across both platforms, whereas the weakly binding systems vary between replicas. Consistent with this, the absolute difference between the early (0–50 ns) and later (50–100 ns) binding fraction across the panel has a median of 12 pp (90th percentile 52 pp), and the inter-replica standard deviation of the binding fraction has a median of 19 pp. These observations support (i) using the equilibrated 50–100 ns window for the reported metrics, (ii) taking replica averages, and (iii) interpreting the binding fraction as a measure of dynamic retention rather than an equilibrium binding constant. A trajectory length of 100 ns is sufficient to characterize the persistent retention of strong binders but not to establish the full binding equilibrium of weakly or intermittently binding ligands.

Table S6. Distance-based close-contact occupancies between ligand polar atoms and active-site residues (5EP8orig).

Ligand (5EP8orig)	Gln153 (%)	Lys108 (%)	Lys188 (%)	His82 (%)
dmap-rR	10.5	22.7	23.7	9.4
dmap-dR	0.2	1.8	1.1	6.1
ind-dR	12.3	1.4	4.0	0.5
ind-rR	0.4	0.0	0.0	0.5

The close-contact occupancy is defined as the fraction of trajectory frames in which any polar atom (N or O) of the ligand is within 3.5 Å of any polar atom (N or O) of a given residue, averaged over the three 5EP8orig replicas. This geometric criterion is a conservative proxy for a hydrogen bond (it counts close polar contacts without applying an angular cutoff); an explicit donor–acceptor hydrogen-bond analysis could not be performed because the GAFF2-parameterized ligands are not recognized by standard hydrogen-bond detectors. The occupancies are modest in absolute terms—direct, persistent hydrogen bonds are rare on this time scale—but they reproduce the selectivity mechanism inferred from the contact-frequency analysis: in the indole pair, the dR analogue contacts Gln153 far more than the rR analogue (12.3 % vs 0.4 %), consistent with a single-residue (Q153) mechanism; whereas in the dimethylaminopurine pair, the rR analogue engages the Lys108/Lys188 cluster (22.7 % / 23.7 %) far more than the dR analogue (1.8 % / 1.1 %), consistent with a cooperative multi-residue network. Values are means of the three replicas.

Table S7. AutoDock Vina docking scores and their divergence from MD retention.

Ligand	1BRW	5EP8min	5EP8orig	5OLN
H-rR	-7.0	-7.41	-7.37	-6.74
H-dR	-6.7	-7.43	-7.1	-6.56
dbap-rR	-7.3	-7.58	-6.67	-6.32
dbap-dR	-7.2	-7.34	-6.34	-6.35
dmap-rR	-7.2	-7.21	-7.08	-6.02
dmap-dR	-6.9	-7.06	-6.88	-6.31
ind-rR	-7.0	-6.92	-6.93	-5.96
ind-dR	-6.8	-7.19	-6.75	-6.48
mmap-rR	-7.1	-7.31	-7.03	-6.0
mmap-dR	-6.8	-7.06	-6.94	-6.04

AutoDock Vina docking scores (kcal/mol) for the best-scoring pose of each of the ten substrate analogues in each conformation, used as the starting pose of the corresponding MD replica (the three inhibitors are not included in this table). Across these 40 ligand–structure combinations the scores span only 1.62 kcal/mol (−7.58 to −5.96), a far narrower dynamic range than the MD-derived binding fraction (0–100 %). Across the panel, the Vina score does not positively predict MD retention; rather, it correlates weakly and non-significantly negatively with the later-window contact binding fraction (BF_contact < 4 Å) (Spearman $\rho = -0.30$, $p = 0.06$). A docking-quality artifact would instead predict a positive correlation, which argues that the conformation-dependent retention reported in the main text reflects an intrinsic property of each pocket rather than a difference in initial docking quality. Reseeding the MD from multiple top poses was identified as a useful follow-up but exceeded the computational budget of this study; the convergence of the strong binders (Supplementary Figure S5) provides complementary evidence that the reported pattern assignments are not artifacts of a single starting pose.

Table S8. Structure-resolved Tyr165 contact and the 1BRW-exclusion sensitivity analysis.

Structure	All, mean (%)	All, median (%)	Late, mean (%)	Late, median (%)
1BRW	55.4	61.6	56.1	56.7
5EP8min	29.6	17.6	30.7	12.0
5EP8orig	14.0	2.7	16.5	0.0
5OLN	28.5	14.8	28.6	4.7

The structure-resolved Tyr165 atomic contact fraction (BF_contact < 4 Å) is reported for both the full (0–100 ns) and later (50–100 ns) windows, as both the per-structure mean and median (n = 39 trajectories per structure). The high closed-group value reported in the main text is borne almost entirely by 1BRW: 5EP8min (mean 29.6 %, full window) is comparable to the open structure 5OLN (mean 28.5 %) and is sufficiently higher only than 5EP8orig (mean 14.0 %). In the sensitivity analysis that excludes 1BRW (comparing the remaining closed-like model 5EP8min with the open group 5EP8orig + 5OLN), the closed-versus-open Tyr165 difference decreases from +21.3 pp (Mann–Whitney $p = 1.2\times10^{-5}$, full panel) to +8.4 pp ($p = 0.07$) in the full window, and to +8.2 pp ($p = 0.044$) in the later window. The Tyr165 lid signal is therefore mainly a property of the 1BRW (functionally closed) state and becomes only marginal once the single cross-species structure is removed; the main-text description was revised accordingly.

Supplementary Discussion S1. The proximity–affinity relationship across the conformational ensemble.

The following discussion of the relationship between physical proximity (retention) and MM-PB(GB)SA binding-energy optimality was moved here from the main text, because once replica-level error bars are attached, the apparent divergence is statistically resolved for only 1 of 13 ligands (Supplementary Table S5) and is therefore not an established result. It is retained here for transparency.

This divergence of the two conformation-resolved rankings deserves emphasis, because it separates two phenomena that are easily conflated. Physical proximity and binding-energy optimality are not the same property of the pocket. The later-window minimum-distance metric (§3.2) ranks the conformations monotonically by pocket closure—the ligand sits closest in the most closed structure (1BRW, 2.62 Å) and moves progressively outward as the pocket expands (5OLN, 4.54 Å). In contrast, the MM-PB(GB)SA binding free energy is non-monotonic and peaks at the semi-open intermediate (the ΔTOTAL_GB of dbap-dR is most favorable in 5EP8orig). Thus the fully closed pocket holds the ligand physically closest to the catalytic residues, whereas it is the partially open pocket that maximizes the computed binding free energy. These two observations are complementary rather than contradictory: pocket closure governs retention (how long and how closely the ligand stays), and the binding-energy landscape governs affinity (how favorably it binds once there), with the latter optimum at an intermediate degree of opening. However, this apparent proximity–affinity dissociation is only weakly supported once replica uncertainty is taken into account. Although 9 of 13 ligands reach their most favorable ΔTOTAL_GB in a non-extreme conformation, the difference from the second-best structure exceeds replica error ($z \geq 1.96$) for only 1 ligand (Supplementary Table S5). We therefore present the proximity–affinity dissociation as a hypothesis suggested by the central tendency of the data but not statistically established at the entropy-uncorrected, single-trajectory GB endpoint of this study; confirming or refuting it would require longer sampling and entropy-corrected free-energy methods. Within this limitation, the data are at least consistent with the closed state acting mainly as a retention state rather than necessarily as the highest-affinity state.

Figure S6. Stability of the backbone conformational state during MD (the lid-loop coordinate).

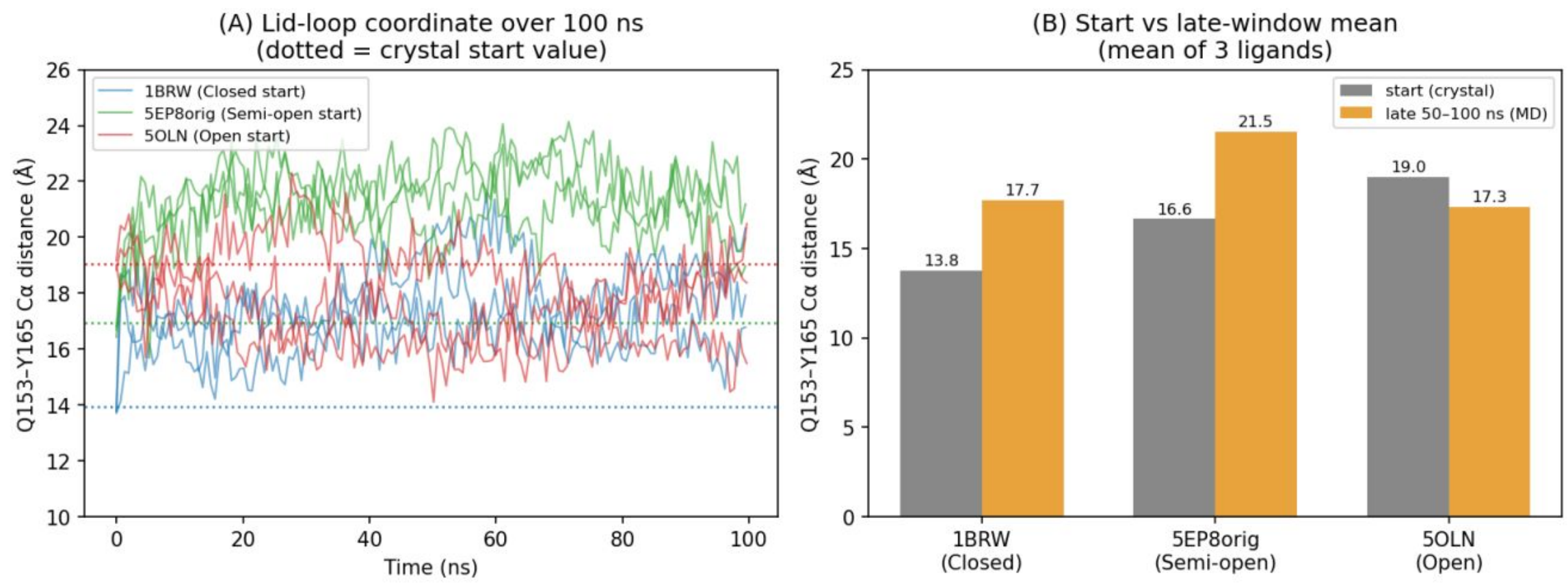

The Q153–Y165 Cα distance—the lid-loop opening coordinate that distinguishes the four crystal states (13.94, 15.23, 16.95, and 19.06 Å for 1BRW, 5EP8min, 5EP8orig, and 5OLN, respectively)—is plotted over the 100 ns production runs for three representative ligands (dmap-rR, dbap-rR, ind-dR) in each of the three crystal structures (distances were computed under the minimum-image convention and are robust to periodic-boundary effects). (A) Individual traces colored by starting structure; the dotted lines indicate the crystal starting values. (B) The per-structure averages of the three ligands compared at the start (crystal) and over the later 50–100 ns window. Each system starts from its assigned crystal value, but the coordinate relaxes during the run: 1BRW opens from about 14 to about 18 Å, 5EP8orig opens further to about 21 Å, and 5OLN contracts slightly to about 17 Å. By the later window, therefore, the four crystallographic labels no longer correspond to four clearly separated basins. We accordingly treat the closed / closed-like / semi-open / open labels as descriptors of the starting crystal conformation rather than as four maintained equilibrium states. This is consistent with our interpretation of the binding-fraction and minimum-distance metrics as measures of retention from the initial docked pose within each starting conformation rather than as equilibrium properties (Methods §2.6; §4.6). This relaxation also reinforces our cautious treatment of the closed-state conclusions, which rest mainly on the cross-species 1BRW structure.

Table S9. Composition of the simulation systems (representative system per structure: dmap-rR).

Structure	Protein residues	Total atoms	Water molecules	Na^+ / Cl^-	Cubic box (nm)
1BRW (Closed)	433	248,359	80,439	233 / 227	13.60
5EP8min (Closed-like)	433	77,857	23,694	86 / 72	9.21
5EP8orig (Semi-open)	433	79,811	24,341	89 / 75	9.28
5OLN (Open)	433	79,887	24,369	93 / 77	9.28

The representative MD system composition for each crystal structure is shown for the ligand dmap-rR (other ligands differ only by a small number of ligand atoms). All systems are the monomer (433 residues), modeled with AMBER ff99SB-ILDN (protein), GAFF2 (ligand), and TIP3P water, neutralized and adjusted to 0.15 M NaCl. All four structures were simulated with the identical protocol (AMBER ff99SB-ILDN / GAFF2 / TIP3P, the same thermostat, barostat, time step, 100 ns length, three replicas each). The 1BRW system was solvated in a larger cubic box (about 13.6 nm) than the *B. subtilis* systems (about 9.3 nm), but both satisfy the minimum-clearance ≥ 1.2 nm criterion (Methods §2.5); the larger 1BRW box only adds bulk solvent and does not affect the intramolecular contact, minimum-distance, and per-residue MM-PB(GB)SA metrics reported here.

Table S10. Sensitivity of the pattern classification to the margin threshold (5 %, 10 %, 15 %).

Ligand	Best (margin, pp)	5 % margin	10 % margin	15 % margin
H-dR	1BRW (17.1)	B	B	B
H-rR	5EP8orig (18.3)	C	C	C
dmap-rR	5EP8orig (3.2)	mixed	mixed	mixed
dmap-dR	1BRW (18.4)	B	B	B
mmap-rR	1BRW (10.2)	B	B	mixed
mmap-dR	5EP8orig (13.6)	C	C	mixed
ind-rR	1BRW (5.5)	B	mixed	mixed
ind-dR	5EP8orig (8.4)	C	mixed	mixed
dbap-rR	5EP8orig (19.1)	C	C	C
dbap-dR	1BRW (5.6)	B	mixed	mixed
Formycin B	5EP8min (8.6)	A	mixed	mixed
TPI	1BRW (49.0)	B	B	B
PIA	1BRW (7.4)	B	mixed	mixed
Classified (non-mixed)	—	12 / 13	7 / 13	5 / 13

Each ligand is assigned to the structure giving the highest replica-averaged BF_contact < 4 Å (pattern A = 5EP8min, B = 1BRW, C = 5EP8orig, D = 5OLN); a ligand is labeled with that pattern only when the best structure exceeds the second-best by at least the margin threshold, and is otherwise labeled "mixed". The "best (margin)" column gives the best structure and its margin (pp) over the second-best. Of the 13 ligands, 12 are classified at the 5 % margin, 7 at the 10 % margin used in the main text, and 5 at the 15 % margin; only dmap-rR (margin 3.2 pp) is "mixed" at all thresholds. Because the best structure itself never changes—only whether the margin exceeds the threshold—the pattern assignments of Table 2 are robust in direction, and the differences between thresholds lie only in how many of the borderline compounds are labeled and how many remain "mixed".

Table S11. Sugar-ring puckering (structure × sugar North/South occupancies).

Structure	Sugar	North % (C3′-endo)	South % (C2′-endo)	n
1BRW	rR	83.5	16.5	15
1BRW	dR	63.8	36.2	15
5EP8min	rR	54.5	45.5	15
5EP8min	dR	37.5	62.5	15
5EP8orig	rR	49.0	51.0	15
5EP8orig	dR	29.5	70.5	15
5OLN	rR	70.2	29.8	15
5OLN	dR	37.9	62.1	15
Overall	rR	64.3	35.7	60
Overall	dR	42.1	57.9	60

From the sugar-ring pseudorotation phase angle P (Altona–Sundaralingam) of each trajectory, the North (C3′-endo, $P \approx 0–36°$) / South (C2′-endo, $P \approx 144–180°$) occupancies were computed and averaged over structure × sugar ($n = 15$ trajectories each = 5 bases × 3 replicas). Overall, ribose (rR) occupied North 64.3 % and 2′-deoxyribose (dR) occupied South 57.9 %, and in the within-(structure × base)-pair comparison ΔNorth(rR − dR) was significant (Wilcoxon $p = 0.021$; trajectory level $p = 8.7\times10^{-5}$). Both puckers were adequately sampled, supporting the validity of the force field and sampling.

Figure S7. Distribution of the sugar-ring puckering (pseudorotation phase angle P).

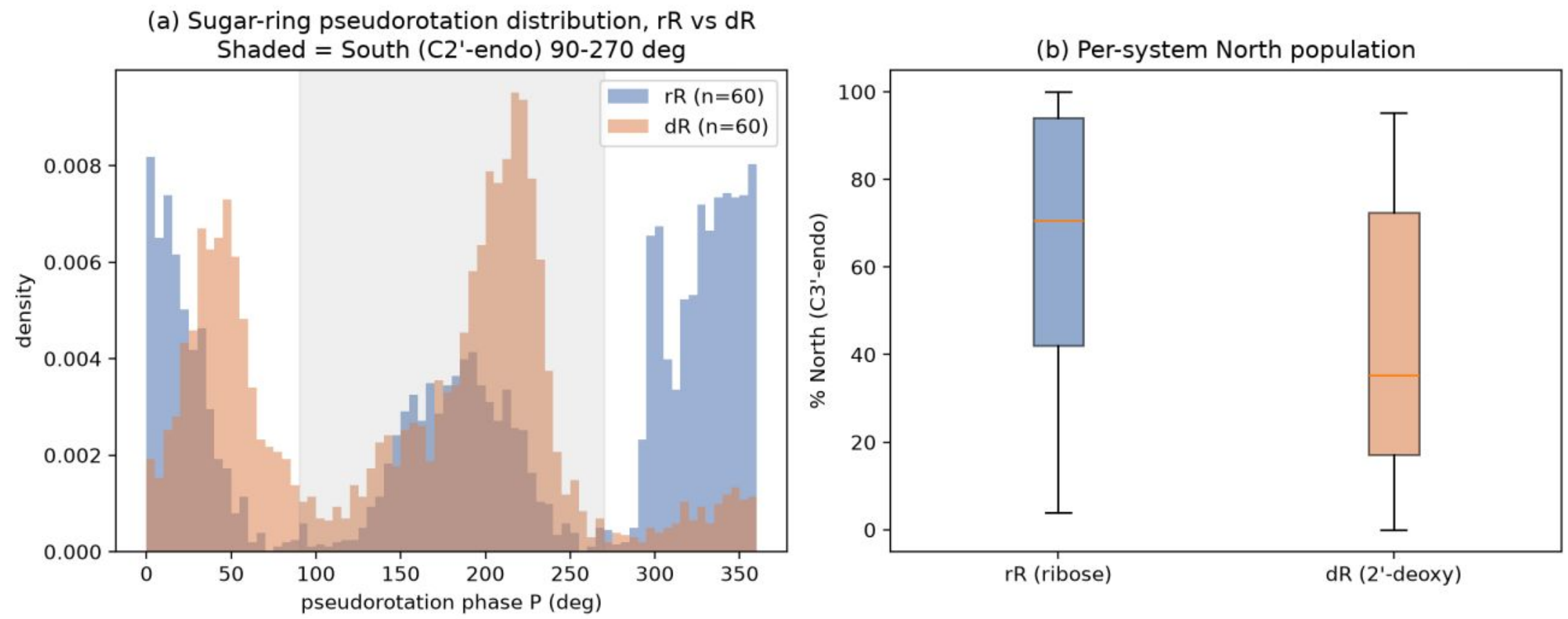

Ribose (rR) prefers North (C3′-endo) and 2′-deoxyribose (dR) prefers South (C2′-endo).

Table S12. Pose states from the periodic-boundary-corrected ligand RMSD (structure-resolved).

Structure	retained (< 5 Å)	shifted (5–15 Å)	dissociated (> 15 Å)
1BRW	6	28	5
5EP8min	6	23	10
5EP8orig	6	21	12
5OLN	3	22	14
Total	21	94	41

At each frame the ligand was minimum-image-corrected (referenced to the nearest image of the pocket centroid) and Kabsch-aligned on the Cα atoms of the seven active-site residues (periodic-boundary-independent), and the heavy-atom RMSD relative to the initial docked pose was computed for all 156 systems. Systems were classified by the later-window (50–100 ns) median RMSD as retained (< 5 Å), shifted (5–15 Å), or dissociated (> 15 Å). The median RMSD of the retained systems is 3.75 Å. The degree of dissociation follows the structural gradient and is mechanistically consistent with the higher retention of the closed states.

Figure S8. Periodic-boundary-corrected ligand RMSD.

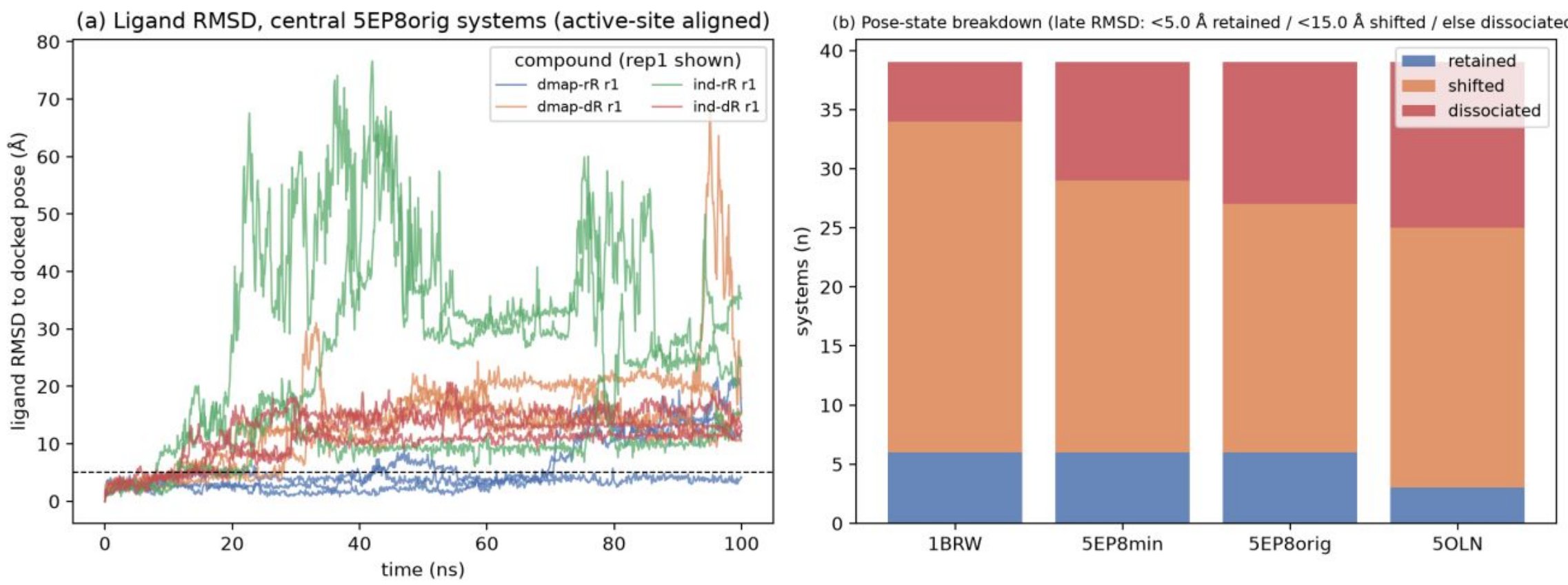

(a) Time series of the 5EP8orig central systems; (b) panel-wide pose-state breakdown (retained 21 / shifted 94 / dissociated 41).

Table S13. Geometric re-measurement of the opening (the two Tyr165-bridge cavity openings).

Structure	State	① Tyr165–Leu114 Cα (Å)	② Tyr165–Gln153 Cα (Å)	closure % (mean of two cavities)
1BRW	Closed	12.82	13.94	100
5EP8min	Closed-like	13.69	15.23	77
5EP8orig	Semi-open	15.01	16.95	44
5OLN	Open	16.98	19.06	0

As the basis for the open/closed classification, the opening widths of the "bridge" by which the Tyr165 side chain divides the active site into two cavities were re-measured on the deposited structures as the Cα–Cα distances from the Tyr165 Cα to the two neighboring residues (① Leu114, ② Gln153), and the closure % was normalized by the mean of the two openings (closure % = $(d\_5OLN - d)/(d\_5OLN - d\_1BRW) \times 100$, 1BRW = 100 %, 5OLN = 0 %). Both openings expanded monotonically (opening ① full range 4.16 Å, opening ② full range 5.12 Å). The closure % (mean of the two openings) is 100/77/44/0 (Table S13), consistent in order, monotonicity, and full range.

Supplementary Section S2. Public deposition of the raw trajectories (Zenodo).

To enable independent verification, all 156 raw trajectories were deposited in the public repository Zenodo (DOI: 10.5281/zenodo.21448920) as periodic-boundary-corrected, solvent-stripped versions (protein + ligand). For each system, md.tpr / md.gro / index.ndx (the Protein_Other group) and the stripped md.xtc (about 67 MB each, about 10.4 GB for all 156) are included; water-inclusive full versions are additionally provided for the main systems. The MD5 checksums of all files (MD5SUMS.txt), the deposition manifest (deposit_manifest.csv, 156 rows), the stripping procedure (strip_all.sh), and the index.ndx generation commands are included. This makes all analyses in this SI (contact, binding fraction, puckering, ligand RMSD, MM-PB(GB)SA) reproducible from the raw data.

Table S14. Independent cross-validation of the sugar-ring puckering (Cremer–Pople vs Altona–Sundaralingam).

Structure	CP %North rR	CP %North dR	Ref.: AS %North rR / dR (Table S11)
1BRW	74.9	53.6	83.5 / 63.8
5EP8min	53.4	44.7	54.5 / 37.5
5EP8orig	51.4	27.3	49.0 / 29.5
5OLN	69.2	35.4	70.2 / 37.9
Overall	62.2	40.2	64.3 / 42.1

To re-validate the North/South occupancies of Table S11 (from the Altona–Sundaralingam pseudorotation phase P) with an independent implementation, the ring-puckering phase P and amplitude Q were recomputed by the Cremer–Pople method for the same 120 substrate trajectories (rR 60 / dR 60). The North (C3′-endo) / South (C2′-endo) boundary was matched to Table S11 (North = $P < 90° \cup P > 270°$), and the per-system %North was compared between the two methods. At the system level ($n = 120$), the per-system %North values of the two methods were moderately correlated (Pearson $r = 0.717$, mean absolute difference MAD = 15.9 %); the absolute occupancies therefore differ between implementations, but the rank order across systems is preserved, so the North/South assignment on which the puckering conclusion rests does not depend on the choice of method. With Cremer–Pople as well, ribose preferred North and 2′-deoxyribose preferred South (structure × base paired test Wilcoxon $p = 0.044$), and the amplitude Q was comparable between sugar types (rR 0.453 Å / dR 0.459 Å), with no difference in pucker depth. The absolute values differ slightly owing to differences in the window and method definitions, but the rR > dR order and monotonicity are preserved in all four structures.

Figure S9. Cross-validation of the Cremer–Pople and the existing puckering methods.

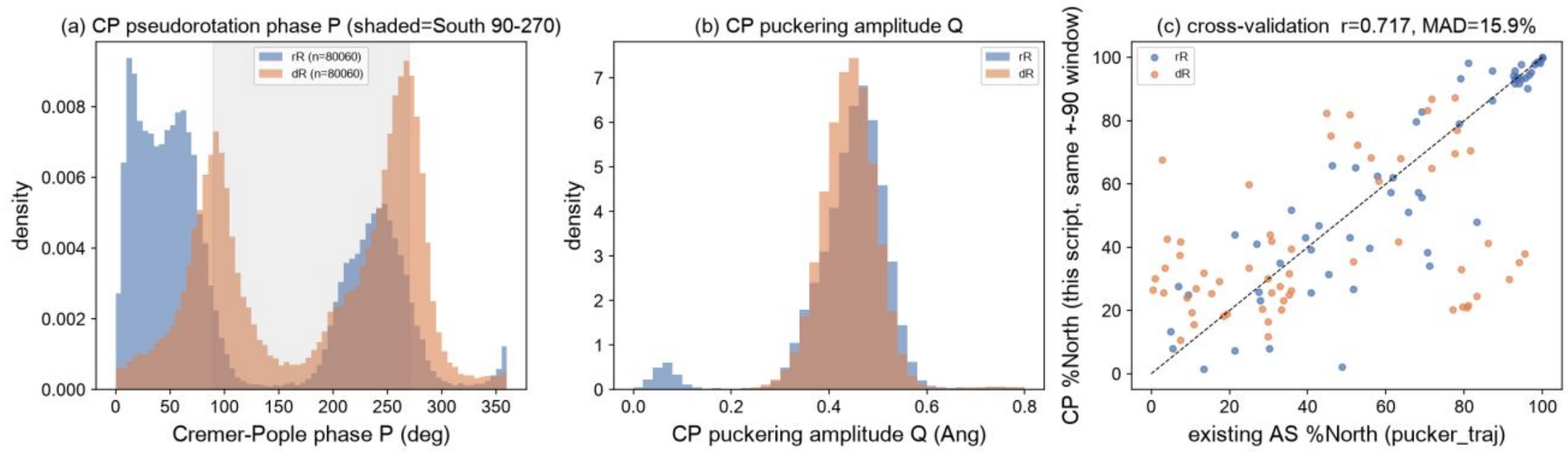

(a) Distribution of the CP pseudorotation phase P (rR North-dominant, dR South-dominant; shading = South 90–270°); (b) CP amplitude Q (comparable between sugar types); (c) per-system %North CP-vs-AS scatter plot (r = 0.717, MAD = 15.9 %, n = 120; dashed line = y = x).

In the unrestrained MD of the intact nucleoside in the absence of phosphate, neither χ (O4′–C1′–N–C) nor the sugar–base plane angle (tilt) showed a systematic difference between the sugar types (rR/dR) (see main text §3.3). The between-sugar χ difference (Δχ(rR − dR)) was a circular mean of −1.7° (≈ 0; 8 positive / 12 negative pairs), and the tilt was rR 62.9° / dR 62.3° (Mann–Whitney p = 0.33, paired test p = 0.84); the effect of the 2′-OH does not appear as a rigid-body reorientation of the base.

Figure S10. Comparison of the glycosidic torsion χ and the sugar–base plane tilt.

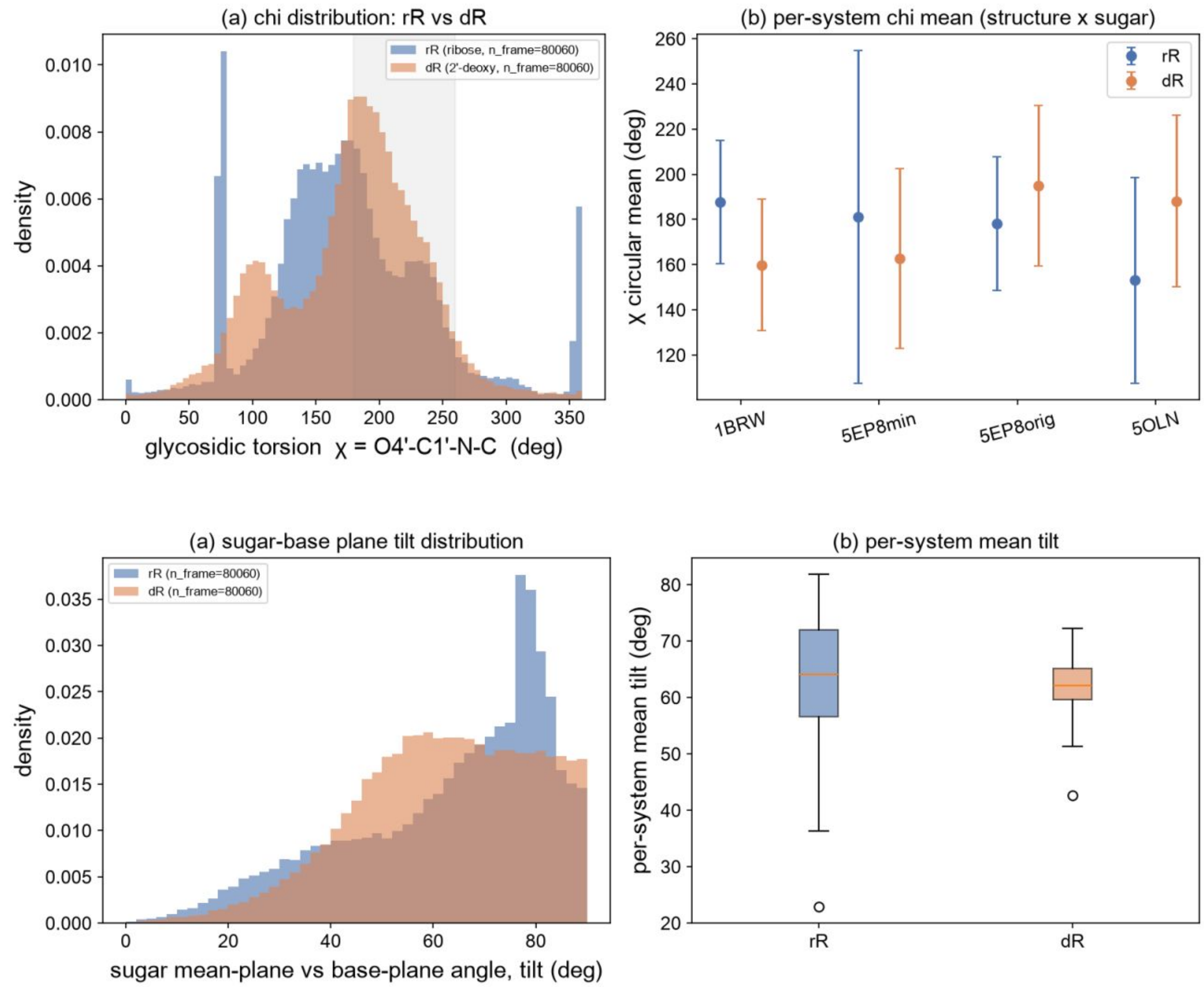

Top: distribution of the glycosidic torsion χ (left) and the per-system mean χ (right, structure × sugar). Bottom: distribution of the sugar–base plane tilt (left) and the per-system mean tilt (right). Neither shows a systematic difference between rR and dR.

Table S15. Cross-species conservation of the active-site residues (*B. subtilis* 5EP8/5OLN vs *Geobacillus stearothermophilus* 1BRW).

B. subtilis residue	5EP8/5OLN (Bs)	1BRW (Gs)	Residue no.	Conservation	Role
Asp80	D	D	80	conserved	phosphate/sugar recognition
Lys81	K	K	81	conserved	phosphate-binding cluster
His82	H	H	82	conserved	catalytic residue (general acid/base)
Lys108	K	K	108	conserved	phosphate-binding cluster
Gln153	Q	Q	153	conserved	sugar-selectivity network
Tyr165	Y	Y	165	conserved	conformational lid
Lys188	K	K	188	conserved	phosphate-binding cluster

In the global alignment of the full-length monomer (BLOSUM62, this study), the sequence identity between *B. subtilis* PyNP and *G. stearothermophilus* PyNP (1BRW) is 68.7 %, but the seven residues that constitute the active site are all strictly conserved with the same residue numbering. The complete conservation in 1BRW of the catalytic residue His82, the lid residue Tyr165, the phosphate-binding lysine cluster (Lys81/Lys108/Lys188), Gln153, and Asp80 indicates that the closed-state active-site environment captured by 1BRW is applicable to *B. subtilis*. As a sanity check, 5EP8 versus 5OLN of the same species were 99.1 % identical.

Figure S11. Cross-species conservation of the active-site residues.

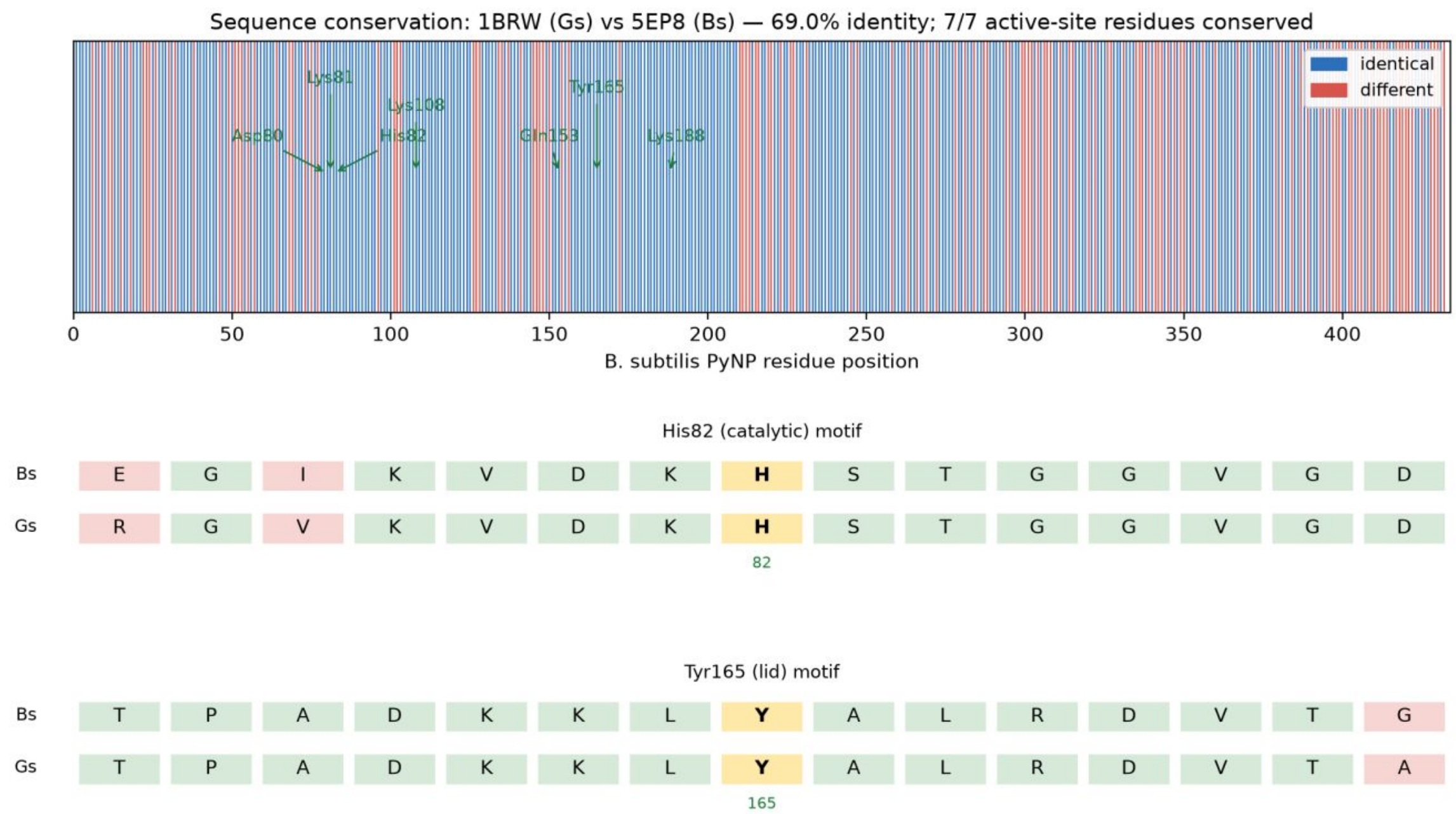

Top: position-wise identity in the full-length alignment of 1BRW (Gs) versus 5EP8 (Bs) (blue = identical, red = different). All seven active-site residues (arrows) are identical. Bottom: close-up of the His82 (catalytic) and Tyr165 (lid) motifs (Bs top / Gs bottom, yellow = the residue in question).

Table S16. Sugar-ring puckering by compound (North = C3'-endo occupancy).

6-substituent scaffold	rR North% (mean ± SD)	dR North% (mean ± SD)	ΔNorth (rR−dR)	n (traj/sugar)
H (unsubstituted)	85.5 ± 20.2	16.9 ± 11.7	+68.6	12
mmap (monomethylamino)	57.7 ± 30.2	36.8 ± 25.6	+20.9	12
dmap (dimethylamino)	38.0 ± 30.6	32.3 ± 27.8	+5.7	12
dbap (dibutylamino)	62.2 ± 21.6	63.6 ± 25.2	-1.4	12
ind (indole)	78.2 ± 24.2	61.1 ± 25.8	+17.1	12

Each cell is the mean of 4 structures × 3 replicas = 12 trajectories. Overall, ribose (rR) North 64.3 %, 2'-deoxyribose (dR) North 42.1 % (= South 57.9 %). Paired test Wilcoxon p = 0.021 (n = 20 base × structure), trajectory level p = $8.7{\times}10^{-5}$. The per-sugar difference (ΔNorth) is largest for the unsubstituted H, shrinks as the 6-position substituent becomes larger, and reverses for dbap.

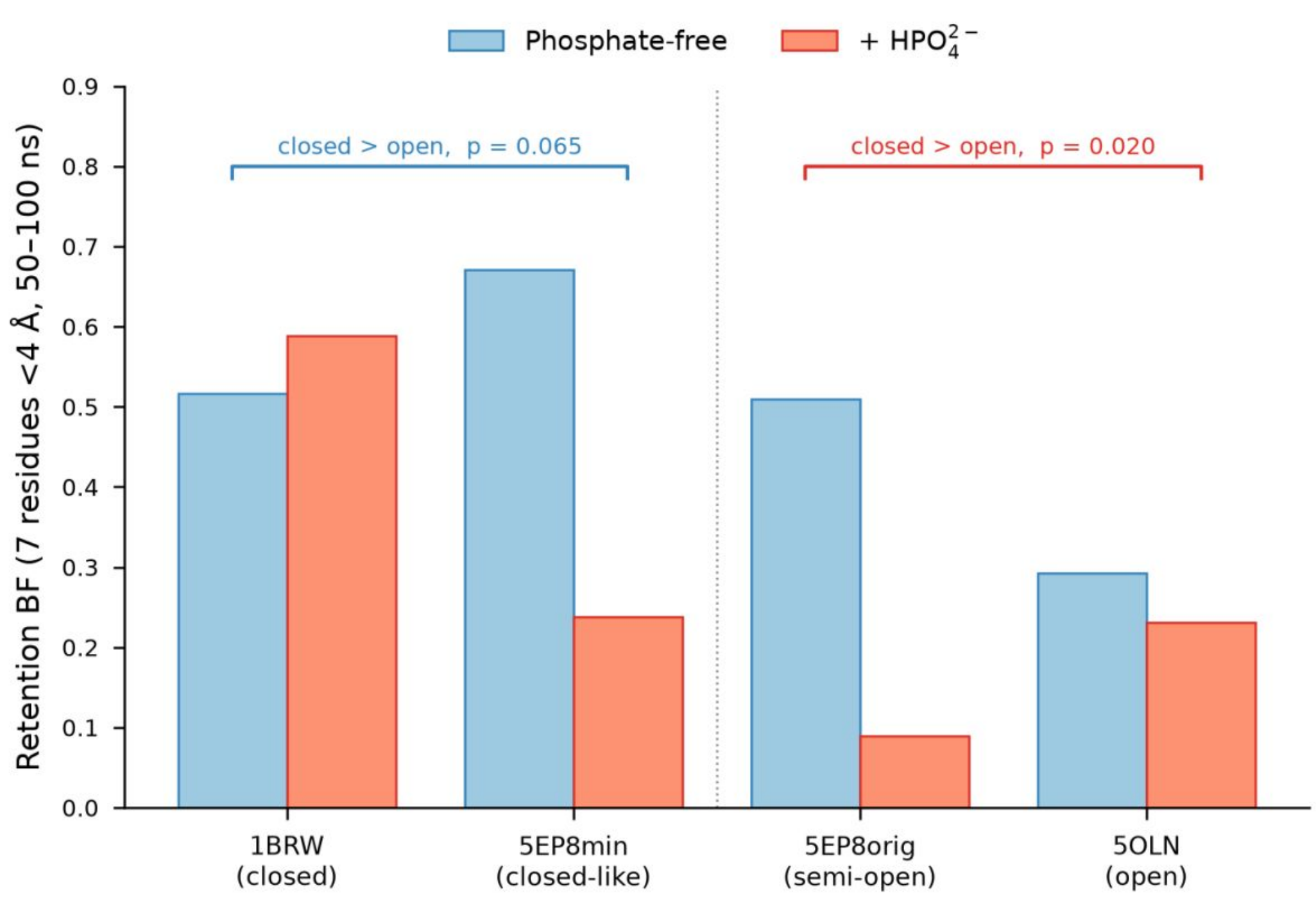

Figure S12. Effect of co-substrate phosphate on state-dependent ligand retention.

Later-window (50–100 ns) contact-based binding fraction (BF; ligand heavy atom within 4 Å of any of the seven active-site residues) for the four compounds run in both conditions (dmap and ind, each as the rR and dR analogue), averaged per conformational state, under phosphate-free (blue) and $+HPO_4^{2-}$ (red) control MD. Phosphate lowers the absolute retention most strongly for the intermediate states (5EP8min 0.67 → 0.24; 5EP8orig 0.51 → 0.09), leaves the open 5OLN nearly unchanged (0.29 → 0.23), and slightly increases the closed 1BRW (0.52 → 0.59). The state-dependent closed > open ordering is preserved and sharpens under phosphate (closed − open Δ = +0.19, p = 0.065 phosphate-free; Δ = +0.25, p = 0.020 with phosphate; one-sided Mann–Whitney).

Supplementary References

(S1) Pettersen, E. F.; Goddard, T. D.; Huang, C. C.; Meng, E. C.; Couch, G. S.; Croll, T. I.; Morris, J. H.; Ferrin, T. E. UCSF ChimeraX: Structure Visualization for Researchers, Educators, and Developers. Protein Sci. 2021, 30 (1), 70–82. DOI: 10.1002/pro.3943.
